# A Chromosome Inversion Creates a Supergene for Sex and Colour in Lake Malawi Cichlids

**DOI:** 10.1111/mec.17821

**Published:** 2025-06-10

**Authors:** Kristen A. Behrens, Soumya Jailwala, Frances E. Clark, Anne C. Meyer, Nikesh M. Kumar, Adrianus F. Konings, Reade B. Roberts, Matthew A. Conte, Jeffrey T. Streelman, Patrick T. McGrath, Thomas D. Kocher

**Affiliations:** ^1^ Department of Biology University of Maryland College Park Maryland USA; ^2^ School of Biological Sciences Georgia Institute of Technology Atlanta Georgia USA; ^3^ Cichlid Press El Paso Texas USA; ^4^ Department of Biological Sciences North Carolina State University Raleigh North Carolina USA

**Keywords:** pigmentation, sex chromosome, structural variant, supergene

## Abstract

Cichlid fishes have the highest rates of evolutionary turnover of sex chromosomes among vertebrates. Many large structural polymorphisms in the radiation of cichlids in Lake Malawi are associated with sex chromosomes and may also carry adaptive variation. Here, we investigate the structure and evolutionary history of an inversion polymorphism that includes both a ZW sex locus and an orange‐blotch colour polymorphism in the rock‐dwelling cichlid fishes of Lake Malawi. We use long‐read sequencing to characterise the sequence and breakpoints of the inversion. We quantify allele frequency differences across the inversion in population samples of the genera *Metriaclima* and *Labeotropheus*. We also examine expression differences of genes in the inversion. The simple inversion spans 7 Mb and is flanked by CACTA transposons that may have catalysed the rearrangement. The region includes ~600 genes, several of which show large differences in expression. Some of these genes are candidates for the sex and colour phenotypes. This inversion is an accessible model system for studying the role of structural polymorphisms and sex chromosome turnover in the adaptive radiation of cichlids in the lakes of East Africa.

## 1 Introduction

Supergenes are clusters of two or more linked genes affecting multiple phenotype traits. Supergenes are often located in regions of little or no recombination, and thus particular allelic combinations segregate as haplotype blocks. Thus, supergenes allow complex phenotypes to be maintained as stable polymorphisms in a local population (Thompson and Jiggins 2014; Schwander et al. 2014; Black and Shuker 2019). The mechanism of recombination suppression is often a structural polymorphism, such as a chromosome inversion, which captures locally adapted alleles (Dobzhansky 1971; Kirkpatrick and Barton 2006). Supergenes have been identified as adaptive polymorphisms in many organisms, including *Heliconius* butterflies (Jay et al. 2018), fire ants (Wang et al. 2013) and fish (Matschiner et al. 2022; Jamsandekar et al. 2024). Many of these inversions persist through multiple speciation events, likely because they carry adaptive variation maintained by balancing selection (Knief et al. 2024). Even distantly related species may develop convergent inversion polymorphisms, perhaps because these genomic regions include genes that affect life‐history traits (MacGuigan et al. 2023).

Sex chromosomes are a special type of supergene and may be affected by the same factors that determine the evolution of supergenes (Thompson and Jiggins 2014). Systems in which a single genetic locus has a major effect on sexual development are defined as XY in species with male heterogamety and ZW in species with female heterogamety. The size and degree of degeneration of the sex‐linked region are highly variable. Sex chromosomes may differ by a single SNP (Kamiya et al. 2012), but they often accumulate a large number of sequence and structural differences that allow them to be distinguished with a light microscope (Cortez et al. 2014; Zhou et al. 2014).

Teleost fish have an incredible diversity of genetic mechanisms for determining sex (Kitano et al. 2024; Wang et al. 2024). Transitions between sex chromosome systems occur at a high rate in many fish lineages, including sticklebacks (Yoshida et al. 2014; Yi et al. 2024), ricefish (Myosho et al. 2015) and poeciliids (Schultheis et al. 2009). African cichlid fishes have the highest rate of sex chromosome turnover known in vertebrates, with approximately 0.25 turnovers per million years of evolution (El Taher et al. 2021; Behrens, Zimmermann, et al. 2024). Of the usual 22 haploid chromosome pairs in African cichlids, only two have not yet been revealed to be sex chromosomes in at least one species (Behrens, Koblmüller, and Kocher 2024).

What are the evolutionary forces responsible for creating this diversity of sex chromosome systems? One idea is that new sex chromosomes arise in response to sexually antagonistic selection (Van Doorn and Kirkpatrick 2007; Werren and Beukeboom 1998). Sexual genetic conflict is pervasive in most species (Rice 1992). Whenever the optimal morphology, behaviour or physiology differs between males and females, selection will favour different phenotypes in the two sexes. This sexually antagonistic selection acts at loci across the genome. Occasionally, novel sex determiners may arise in linkage with a particular set of sexually antagonistic variation, allowing resolution of the sexual antagonism via sex‐limited alleles. For example, an allele that improves female fitness, but which decreases the fitness of males, will favour the invasion of a dominant female sex determiner tightly linked to the allele, improving female fitness (Van Doorn and Kirkpatrick 2010).

**FIGURE 1:**
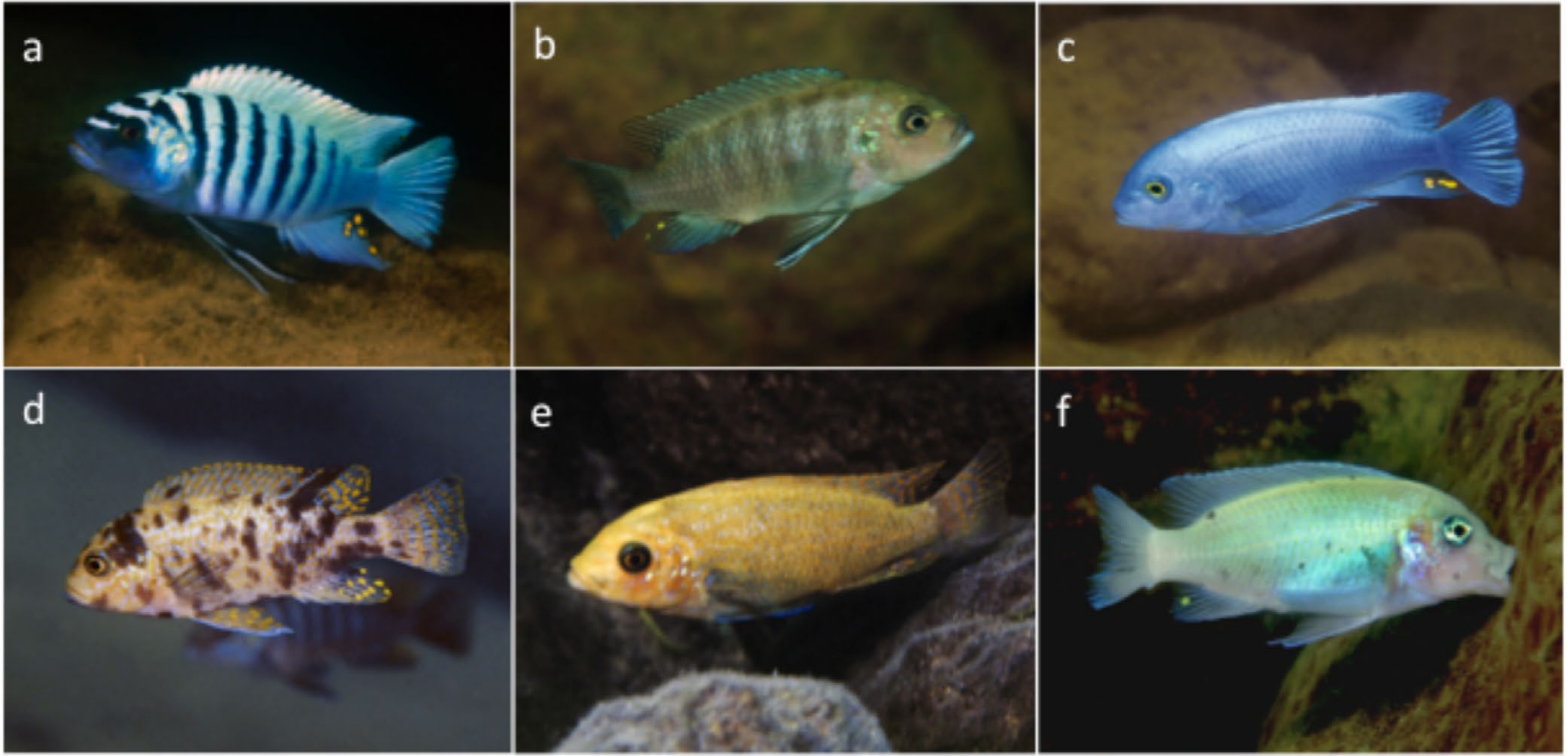
Colour morphs of rock‐dwelling ‘mbuna’ cichlids from Lake Malawi. (a) *Metriaclima zebra* BB male, (b) *M. zebra* BB female, (c) *M. callainos* blue male, (d) *M. zebra* OB female, (e) *M. zebra* O female, (f) *M. callainos* white female. Blue males and blue females are nearly indistinguishable, as are white males and white females. Four examples of the OB male phenotype are presented in Figure 3. Photos by Ad Konings.

Cichlid fishes are an interesting system in which to investigate these questions. In East Africa, this group underwent several rapid radiations, generating more than 1000 species which differ in behaviour, pigmentation and other morphological characteristics (Burress 2015; Santos et al. 2023). The males of many rock‐dwelling ‘mbuna’ cichlids in Lake Malawi have highly specific nuptial colour patterns, many of which are based on a bright blue background interrupted by a series of black vertical bars (BB morphs). The phenotype of the corresponding BB morph females is a typically drab brown background with faint vertical bars, a more cryptic pattern that may decrease predation. However, some individuals sport a novel orange‐blotch (OB) colour pattern (Figure 1). OB morph fish are typically female, although OB males do occur at low frequencies in several populations.

The OB colour morph appears in various forms in dozens of species in four genera of Lake Malawi cichlids, ranging from a solid orange or white colouration to morphs with various levels of blotching (Konigs 2001; Roberts et al. 2009). Depending on the base colour and the degree of blotching, several additional descriptive categories of ‘OB’ morph have been distinguished, including a relatively unblotched orange morph (O), a white morph (W) and a blotched white morph (WB). All of these variants which disrupt the normal BB colour pattern map to the same genomic location, although it is not clear whether all of the differences in background colour or degree of blotching are due to variation in the OB haplotype or might also involve variation at background loci elsewhere in the genome.

Analysis of segregation in laboratory crosses demonstrated that OB morphs are due to a dominant allele that is tightly linked to a ZW sex determiner (Streelman et al. 2003; Roberts et al. 2009). The ZW locus on linkage group (LG) 5 is epistatically dominant to an XY locus on LG7 (Ser et al. 2010). Using a combination of genetic mapping in laboratory crosses and association mapping in natural populations, we mapped the OB variant to a conserved haplotype on LG5, and suggested that the colour polymorphism is due to mutations that alter expression of the *pax7a* gene (Roberts et al. 2009). Much of the variation in blotching among OB individuals appears to be due to differences in *pax7a* gene expression among OB alleles, and some populations segregate several discrete OB phenotypes that correspond to unique *pax7a* haplotypes (Roberts et al. 2017). OB alleles have also been associated with significant differences in gut length, as well as craniofacial and body morphology, suggesting that this locus has phenotypic impacts beyond sex and colour (Moore et al. 2022). However, the gene responsible for the heterogametic female (ZW) mode of sex determination has not been identified.

We have suggested that the evolution of the ZW sex determination system resolved an intersexual genetic conflict over the OB colour patterns, which appear to be favoured in females but selected against in males (Roberts et al. 2009). OB patterns are thought to reduce predation on females by providing an alternative form of crypsis. On the other hand, the blotched pattern breaks up the nuptial colouration of OB males, which may decrease their mating success. A reduction of recombination between the loci controlling sex and colour would thus preserve the selectively favoured allelic combinations (OB females and BB males).

Inversions are known to suppress recombination on sex chromosomes (Bergero and Charlesworth 2009; Wang et al. 2012), and have long been understood to play a role in evolution and speciation (Ayala et al. 2013; Kirkpatrick 2010; Lowry and Willis 2010). However, the impact of multifarious selection forces acting on these rearrangements makes their persistence challenging to predict. Inversions have been grouped into four general types defined by the type of selective pressure they experience (neutral, overdominant, directly beneficial and indirectly beneficial), and evolve differently based on the initial inversion size (Connallon and Olito 2022). Association with adaptive traits, which could be considered directly or indirectly beneficial, is one way an inversion may persist long term (Berdan et al. 2023). Another proposed mechanism for prolonging segregation of an inversion is the accumulation of deleterious mutations that are private to each arrangement, resulting in a selective advantage for heterozygotes (Berdan et al. 2021). Sex‐linked inversions face the same challenges as autosomal inversions; however, the length of sex‐linked inversions is likely important in the context of selective processes (Charlesworth and Harkess 2024; Olito et al. 2024). Modelling has shown that intermediate to large inversions may persist under overdominant or local adaptation conditions (Connallon and Olito 2022). The role of these local adaptation scenarios leads us to consider sex‐determining inversions from a supergene perspective.

In this study, we aimed to characterise the structure and sequence of the OB allele, compare wild populations of Lake Malawi cichlids with OB phenotypes, and identify candidate genes for the OB phenotype and sex.

## 2 Materials and Methods

### 2.1 Genome Sequencing

High‐molecular weight DNA was prepared from the blood of a single *M. zebra* (Masinje) blue male with blotching and a single *L. trewavasae* (Thumbi West) female that had the solid orange variant of the OB phenotype (Figure S1). Both were obtained from a commercial source in the United States. Samples were collected according to procedures approved by the Georgia Institute of Technology (IACUC #A100029 and A100569) and all experiments were conducted in accordance with the Guide for Care and Use of Laboratory Animals (Institute for Laboratory Animal Research 2011). DNA was extracted from blood using the Nanobind Tissue RT kit (Pacific Biosciences, Menlo Park CA). The Short‐Read Eliminator (> 25 kb enrichment) kit (Pacific Biosciences) was used to remove shorter reads from the sample. DNA concentrations were quantified by fluorescence spectroscopy using a Quant‐iT PicoGreen assay (ThermoFisher, Waltham MA, USA). At Maryland Genomics (University of Maryland, Institute for Genome Sciences), samples were size selected on a BluePippin pulse‐field gel system (Sage Science, Beverly MA, USA), sequencing libraries were constructed and extra‐long PacBio HiFi sequencing was conducted on a Revio sequencer (Pacific Bioscience, Menlo Park CA, USA).

### 2.2 Genome Assembly

Genome assemblies were constructed using hifiasm v0.19.8 (Cheng et al. 2021; Wang 2022) with the default purge_dups setting on, which generates a primary assembly as well as two haplotig assemblies. Bandage (Wick et al. 2015) was then used to evaluate support for merges of contigs. Contigs for each assembly were then aligned against the *M. zebra* genome from a normal blue‐barred phenotype individual (UMD2a, GCF_000238955.4) (Conte et al. 2019) using D‐Genies (Cabanettes and Klopp 2018) to determine the placement of contigs into LGs. This initial alignment was used to inform preliminary manual merges of contigs into scaffolds, where a string of 50 N was used between two contigs to indicate a gap. When necessary, contigs were reverse complemented with seqtk (https://github.com/lh3/seqtk). The Telomere Identification Toolkit (Tidk, v0.2.31) (https://github.com/tolkit/telomeric‐identifier) was used to detect repeats with a pattern consistent with a telomeric identity. Once the telomeric repeat was detected (AACCCT and its reverse complement), its location was used to guide the correct orientation of contigs when scaffolding.

To characterise whether the inversion was in one or both haplotypes, we used D‐Genies to align both haplotigs assembled by hifiasm to the UMD2a reference genome and to each other for both species. Additional alignments of the *L. trewavasae* inversion haplotype against the UMD2a *M. zebra* genome and the MezeOBm *M. zebra* genome were conducted using minimap2 v2.28 (Li 2018) with the ‐asm20 flag. To characterise the genes in the inversion region, the genomes were softmasked using RepeatMasker v4.1.5 (DFam, curated only) (Smit et al. 2013), annotated using Braker3 v3.0.8 with default settings and no informative sequences (Gabriel et al. 2024) and annotations were functionally characterised using eggNOG v2.1.8 (Cantalapiedra et al. 2021). Transposable elements (TEs) were also annotated for both genomes using edta v2.1.0 (Ou et al. 2019). These analyses utilised the Galaxy server (The Galaxy Platform for Accessible, Reproducible, and Collaborative Data Analyses 2024). The fraction of bases annotated as TEs by edta per 200 kb window was calculated using a custom R script (https://github.com/ZexuanZhao/Pegoscapus‐hoffmeyeri‐sp.A‐genome‐paper). Proteins for genes of interest were characterised using coordinates gleaned from aligning the gene of interest to the LatrZW genome, which features the inverted LG5 haplotype. From there, the genes of interest were extracted from the genome assembly with Samtools v1.17 faidx, and the protein sequence predicted with Genewise (Madeira et al. 2022) using the corresponding gene annotated in the *M. zebra* (UMD2a) reference for guidance.

Synteny was calculated using MUMmer4 v4.0.0 (Marçais et al. 2018), first using the nucmer sub‐program at default, then delta‐filter (‐1 ‐l50 ‐i 50 ‐u 50), and finally show‐coords (‐l 50 ‐L 50 ‐l ‐T ‐H). Synteny plots were generated using SyntenyPlotteR (Quigley et al. 2023).

### 2.3 Quality Assessment

Genome assembly quality was assessed using the Genome Evaluation Pipeline (GEP) (https://git.imp.fu‐berlin.de/cmazzoni/GEP), which contains the following programs: GenomeScope2 (https://github.com/tbenavi1/genomescope2.0), meryl and merqury (Rhie et al. 2020), BUSCOv5 (Manni et al. 2021) and a modified version of assembly_stats (*v0.1.4)* (http://doi.org/10.5281/zenodo.3968775). The *M. zebra* (UMD2a) assembly (Conte et al. 2019) was used as a guide for expected chromosome size. Coverage was calculated using samtools depth (Danecek et al. 2021).

### 2.4 Population Samples

Samples were collected using SCUBA and sexed by dissection and visual inspection of gonads in the field. For this study, the following populations were (Table S1) considered (Table 1). One population of *Metriaclima (Maylandia) zebra* (Nkhata Bay), three populations of *M. callainos* (Nkhata Bay, Luwino, Lupingu), one population of *M*. sp. ‘*zebra gold*’ (Nkhata Bay), two populations of *Labeotropheus trewavasae* (Chilumba—Maison Reef, Thumbi West Island) and one population of *L. fuelleborni* (Thumbi West Island). Animal care and use were approved under University of Maryland animal care protocol R‐11‐77, and fish collections in Malawi were made in 2012 in accordance with the Memorandum of Understanding between the Malawi Department of National Parks and Wildlife and the Department of Biology of Chancellor College (University of Malawi). Fin clips were preserved in DMSO‐salt buffer (Seutin et al. 1991). DNA was extracted from the fin clips using standard phenol‐chloroform extraction and ethanol precipitation (Kocher et al. 1989). DNA concentrations were quantified by fluorescence (Picogreen, Thermo Fisher Scientific, Waltham, MA USA). Sample DNA concentrations were then normalised and pooled for sequencing. We constructed separate pools for each sex/morph for each population. For example, for *M. zebra* from Nkhata Bay, we constructed separate pools for BB males, BB females, OB females and O females.

**TABLE 1:** Samples analysed by pooled sequencing.

Locality	Species	Sex	Morph	Sample size
Nkhata Bay GPS −11°36.370S, 34°18.207E	*Metriaclima zebra*	Male	BB	20
	*M. zebra*	Female	BB	20
	*M. zebra*	Female	OB	20
	*M. zebra*	Female	O	20
	*M. ‘zebra gold’*	Male	BB	20
	*M. ‘zebra gold’*	Female	OB	20
	*M. callainos*	Male	Blue	20
	*M. callainos*	Female	Blue	20
	*M. callainos*	Female	White	20
Chilumba—Luwino Reef GPS −10°26.836S, 34°16.907E	*M*. ‘*callainos* Luwino’	Male	White	20
	*M*. ‘*callainos* Luwino’	Female	White	20
Lupingu GPS −10°05.671S, 4°32.160E	*M. ‘callainos* Lupingu’	Male	White	10
	*M. ‘callainos* Lupingu’	Female	White	12
Chilumba—Maison Reef GPS −10°28.636S, 34°17.731E	*Labeotropheus trewavasae*	Male	BB	13
	*L. trewavasae*	Female	BB	18
	*L. trewavasae*	Female	OB	17
Thumbi West Island GPS −14°1.457S, 34°49.438E	*L. fuelleborni*	Male	BB	12
	*L. fuelleborni*	Female	BB	10
	*L. trewavasae*	Male	BB	12
	*L. trewavasae*	Female	BB	11

### 2.5 Pooled Sequencing

DNA was prepared for sequencing using the TruSeq DNA Sample Prep kit v2.0 (Illumina, San Diego CA, USA). In brief, the DNA was sheared to less than 800 bp using an S220 focused ultrasonicator (Covaris, Woburn MA, USA), and the ends repaired with polymerase and polynucleotide kinase. The ends of the fragments were adenylated to prepare for the ligation of bar‐coded adapters unique to each DNA pool. Fragments of 300–500 bp were gel‐purified prior to PCR enrichment of fragments with adapters on both ends, followed by cluster generation and sequencing on the HiSeq1500 platform (Illumina, San Diego CA, USA). For each population, we obtained one lane of 2 × 100 bp Illumina HiSeq data from each sex/morph (Table 1), corresponding to ~1× coverage of the cichlid genomes in each pool (2 Gb/individual × 20 individuals = 40 Gb of sequence).

### 2.6 Sex‐Specific SNP Analysis

Sex‐specific SNPs were identified following our methods described previously (Behrens et al. 2022) using the sex‐SNP‐finder pipeline (Gammerdinger et al. 2018). Previously reported code from that study is available (https://github.com/Gammerdinger/sex‐SNP‐finder). Briefly, the sequence reads were aligned with BWA v0.7.12 using the default parameters along with read group labels to the new high‐quality reference assemblies, *L. trewavasae* (SUB14832768) and *M. zebra* (SUB14832768). At each variable nucleotide site, we calculated the *F*_ST_ statistic (the *G*_ST_ of Nei 1973) between the populations of male and female sequence reads using Popoolation2 (Kofler et al. 2011). The resulting *F*_ST_ plots provide a first indication of the differentiation between the morph/sexes compared in each population. We further identified XY‐ and ZW‐patterned SNPs as SNPs that were fixed in one sex and polymorphic in the other sex. We established a cutoff frequency of greater than 0.9 to indicate fixation, to allow for sequencing errors. Likewise, while we expect a Y allele frequency of 0.5 in males, we used cutoffs of 0.3 < *x* < 0.7 to account for sampling variation. Separate plots of the allele frequency of XY‐ and ZW‐patterned SNPs suggested the type of heterogametic system segregating (XY or ZW). We used Bedtools v2.30.0 to *make windows* and *coverage* to calculate the density of sex‐patterned SNPs in 100 kbp windows across the genome to better visualise the region.

### 2.7 Individual Genotyping

To evaluate the genetic diversity of the inversion among morphs and populations, we genotyped five populations ( *M. zebra* Nkhata Bay, *M*. ‘*zebra gold*’ Nkhata Bay, *M. callainos* Nkhata Bay, *M. callainos* Luwino and *L. trewavasae* Maison Reef) for three microsatellites within the inversion. We genotyped approximately 20 individuals for each sex/morph in each population. The primers used were: gata2F 5′ACAATTTGTATGGTGACTGGGA3′, gata2R 5′GCGCTGACCTACTTCTCTGTGA3′; Pax7F 5′TGGGATTTGGGAGGAATGGG3′, Pax7R 5′GCATAGGGAGGAGGGCATTGT3′; FibroleukinF 5′CATCTCTGTGTGTGGCTGTG3′, FibroleukinR 5′GCTCCTGCTAGTGAGGTTGCT3′. We also genotyped two populations of *M. callainos* for OB‐specific SNPs in a putative sox10 enhancer of the *pax7a* gene. The nested primers used were Pax7OuterF 5′TGGCTTAAACAACTGGAGCA3′, Pax7OuterR 5′ACACAGTTAGGCAGCAAACA3′; Pax7InnerF 5′GCAGACTCCTCTTGCTTTCT3′, Pax7InnerR 5′GGTGAATCGGCAATGCATGA3′. PCR products for each individual were amplified with an annealing temperature of 55°C and then sized/sequenced on an ABI 3730 DNA sequencer (Thermo Fisher Scientific, Waltham Massachusetts).

### 2.8 Transcriptome Analysis

Gonads were dissected from 40 individual larvae from lab stocks of three species at the earliest practical stage: *Labeotropheus trewavasae* (14 days post fertilisation), *Aulonocara baenschi* (14 dpf) and *Oreochromis niloticus* (28 dpf). Each larva was genotyped for sex‐specific microsatellite markers to allow pooling of male and female gonads for RNA extraction. mRNA was reverse transcribed and prepared for sequencing using the TruSeq DNA Sample Prep kit v2.0 (Illumina, San Diego CA, USA). Approximately 392 million 100 bp paired end reads were obtained for each sex in each species (a total of 6 lanes of Illumina HiSeq1000 sequencing). Reads were aligned to the new *L. trewavasae* genome to identify differentially expressed genes and calculate FPKM values for male and female samples. In short, reads were aligned with Hisat2 v3 (Kim et al. 2019), and analysed with the Cufflinks v2.2.1 pipeline (Trapnell et al. 2012) to identify differentially expressed transcripts.

We further investigated the inverted region for coding variants with potential effects on genes of interest. Variant calling was conducted from *L. trewavasae* raw PacBio HiFi reads aligned against UMD2a using minimap2. GATK v4.2.6.1, a version compatible with minimap2, was used to call variants from the alignment using default settings (Van der Auwera and O'Connor 2020) and svim v2.0.0 (Heller and Vingron 2019) was used to call larger structural variants from the long reads. The functional effect of SNPs from both outputs was evaluated using snpEff v.4.5 (Cingolani et al. 2012).

## 3 Results

### 3.1 Genome Assemblies

Here, we present new high‐quality genome assemblies for a *L. trewavasae* O female (LatrZW) and a *M. zebra* OB male (MezeOBm). Contig N50 in both assemblies was roughly equal to chromosome size in cichlids, and BUSCO single copy scores indicate a high level of completeness (Table 2). The metric for log‐scaled probability of error for the consensus base calls, consensus quality (QV), was 65.37 in the male and 61.91 in the female, indicating a very accurate consensus. K‐mer completeness was 95.27% in the male and 94.59% in the female. Overall, these metrics indicate high‐quality genome assemblies. Additionally, a B chromosome was identified in each genome that will be addressed in a separate study. We found that each genome was heterozygous for an inversion on LG5, which meant we were able to characterise two haplotypes for LG5 in both genomes owing to the large amount of divergence between them resulting from the inversion. As the LG5 inversion haplotig was more complete in the LatrZW assembly, we use this genome for our primary analyses.

**TABLE 2:** Genome assembly statistics.

	*M. zebra* (OB male) (ASM4771593v1)	*L. trewavasae* (OB female) (ASM4766397v1)
Genome size	945,806,322 bp	937,277,439 bp
Genome coverage	103×	102×
Number of chromosomes/scaffolds	22 + 1 unpaired	22 + 1 unpaired
Total number of contigs	170	344
GC %	41.24%	41.2%
Scaffold N50	36,798,361 bp	39,702,102 bp
Contig N50	31,105,378 bp	37,729,116 bp
Complete BUSCOs	98.7%	98.2%
Complete and single‐copy BUSCOs	97.4%	96.9%
Complete and duplicated BUSCOs	1.3%	1.3%
Fragmented BUSCOs	0.4%	0.4%
Missing BUSCOs	0.9%	1.4%

### 3.2 Characterisation of the Inversion

**FIGURE 2:**
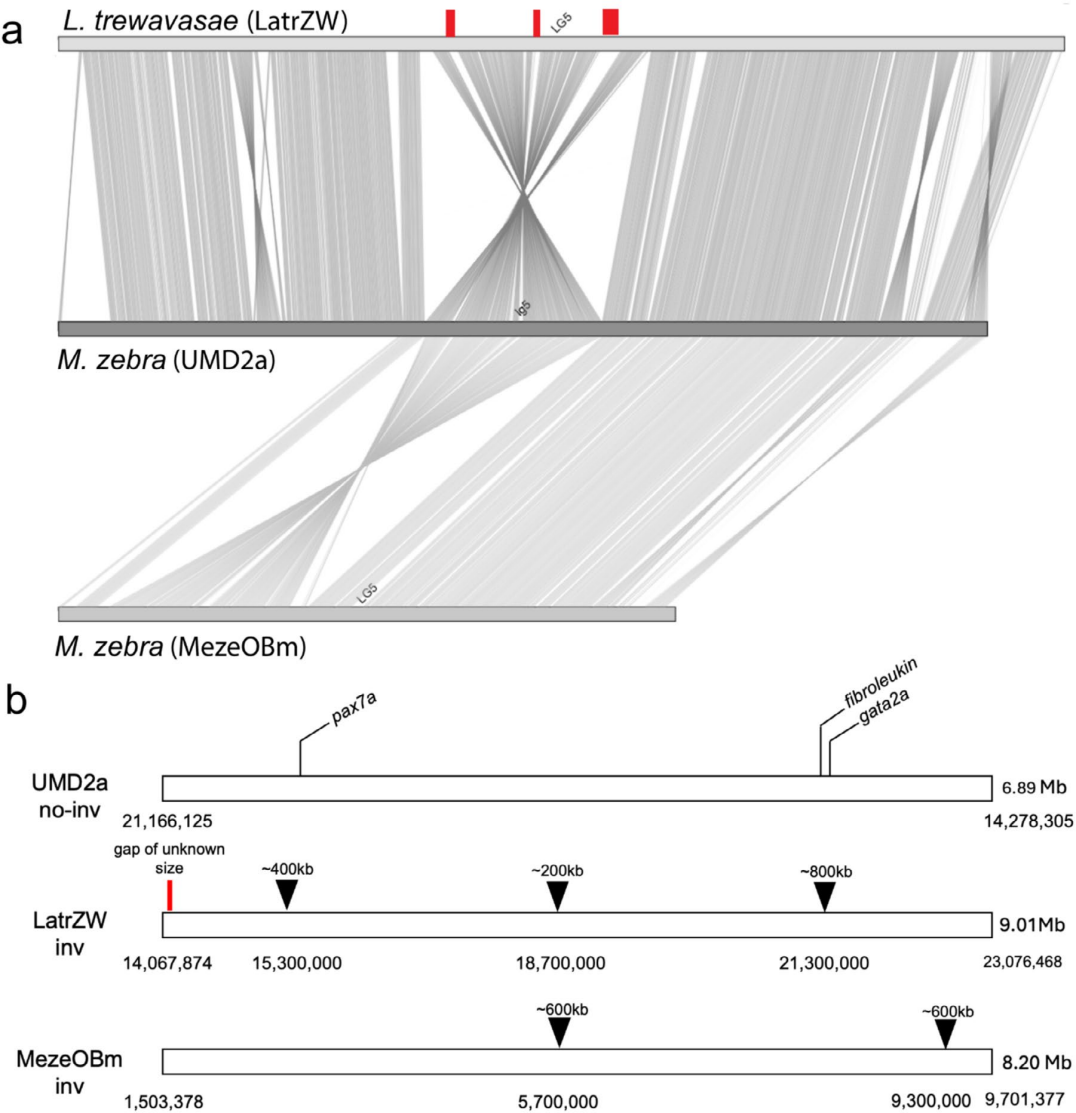
Comparison of the normal and inverted regions on LG5. (a) Plot of the relative gene locations between the *M. zebra* UMD2a reference and the inverted haplotype of *L. trewavasae* and *M. zebra* . Regions of poor alignment of UMD2a against *L. trewavasae* are indicated by red bars. (b) Detailed coordinates in the UMD2a reference and the inverted haplotypes from the LatrZW and MezeOBm assemblies. Inverted triangles denote regions of high transposable element density in the inverted sequences. A gap of unknown size in LatrZW indicates where two contigs were joined based on the reference.

The inversion spans 6.89 Mb in the UMD2a reference assembly, or about 19% of LG5. The inversion is located in the middle of the chromosome, a region of relatively high recombination far from the centromere or telomeres (Conte et al. 2019). Alignment of the inversion haplotypes from each species showed that they share the same breakpoints, and that the overall structure is a simple inversion. Figure S2 shows the pairwise comparisons of the two new genomes to *M. zebra* UMD2a and to each other, including a comparison of the inverted and non‐inverted haplotypes for each new genome. The inverted (OB) haplotype of the MezeOBm assembly spans 8.20 Mb, about 1.3 Mb longer than the corresponding region of UMD2a. The difference is largely explained by the expansion of repetitive sequence at two locations within the inversion. The inversion is contained within a single haplotig. The remaining portions of LG5 are not represented by large contigs in this assembly. The inverted haplotype of the LatrZW assembly spans 9.01 Mb, or about 2.1 Mb longer than the corresponding sequence in UMD2a. Only about half of the difference in length is explained by the expansion of repetitive sequence at three locations within the inversion. This haplotype consists of two haplotigs scaffolded into a single chromosome, and the join falls close to one end of the inversion at approximately 14,111,000 bp. The location of only one of the repetitive sequence expansions is shared between the LatrZW and MezeOBm assemblies (Figure 2).

TEs have accumulated in the inverted haplotypes. The mean fraction of base pairs that were TEs across the whole LatrZW genome was 0.26. In regions of LG5 outside the inversion, this fraction was 0.27, but inside the inversion, the fraction was 0.31. These differences were significant in a one‐tailed *T*‐test (*p* = 0.028 comparing the inversion to the whole genome, and *p* = 0.022 compared to regions of LG5 outside the inversion). The fraction of bases annotated as TEs per 200 kb window in the LatrZW inversion peaked at the three locations marked in Figure 2 (Figure S3a). The MezeOBm inversion haplotype genome also showed peaks of TE density corresponding to the two locations marked in Figure 2 (Figure S3b). No such peaks of high TE density are present in the non‐inverted haplotypes. Examination of the TE families at the breakpoints found CACTA TIR transposons in the vicinity of both breakpoints (Table S1). CACTA TIR transposons were also the most frequent TEs in the inversion at 33% (Figure S4).

### 3.3 Analysis of Pooled Sequencing

**FIGURE 3:**
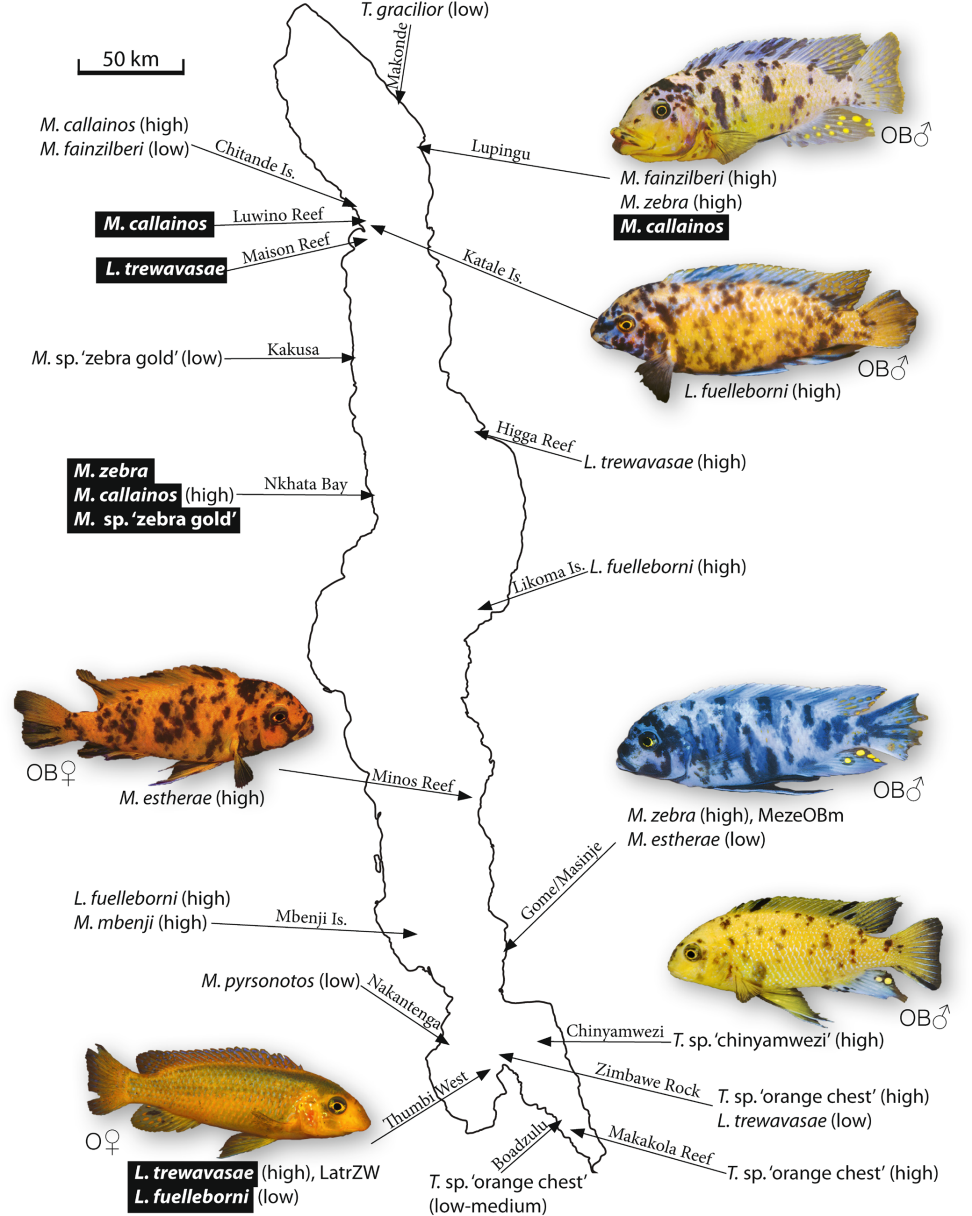
An assessment of the frequency of OB males in populations around Lake Malawi. High frequencies are defined as populations with greater than 1% OB males, while low frequencies are defined as populations with less than 1% OB males. These frequencies were estimated through informal surveys by Ad Konings. Not indicated are many other populations segregating OB that do not have notable frequencies of OB males. Species/populations with OB poolseq data are indicated in bold.

**FIGURE 4:**
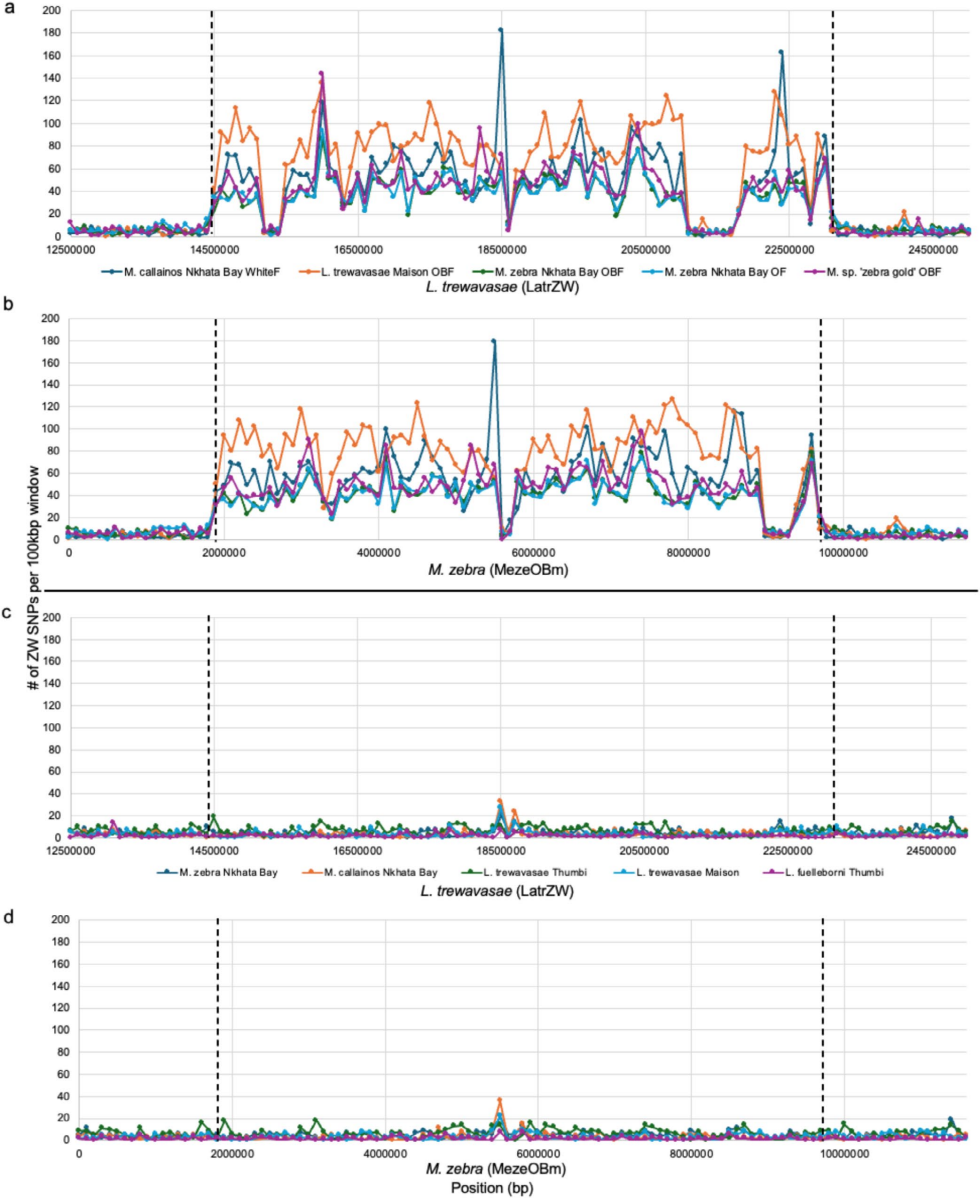
Plots of ZW sex‐specific SNP density across the inverted region for comparisons of males and females analysed by pool‐seq. Comparisons of BB males to various OB females plotted against (a) *L. trewavasae* (LatrZW) and (b) *M. zebra* (MezeOBm). Comparisons of BB males to BB females (i.e., not including an inversion difference) plotted against (c) *L. trewavasae* (LatrZW) and (d) *M. zebra* (MezeOBm). Dashed lines indicate approximate inversion boundaries.

To better understand the origin and evolution of the inversion, we sequenced 8 populations (6 nominal species) of two genera from five localities around the lake (Figure 3). We aligned pool‐seq data from wild‐caught males and females of the different phenotypes from each population to both new genomes. As expected, comparison of BB or B males to BB or B females showed no signal of sex‐specific SNPs on LG5, indicating the absence of a sex‐linked inversion (Figure 4).

**FIGURE 5:**
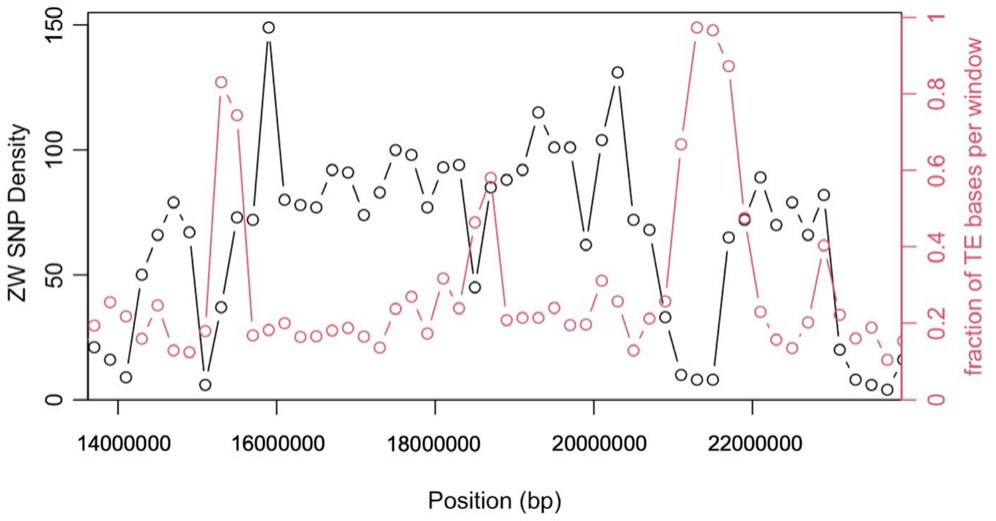
Decreases in ZW SNP density within the inversion correspond to regions of high transposable element density. The fraction of bases annotated as transposable elements is plotted along with the ZW sex‐specific density in 200 kb windows across the inverted region of LG5 in the LatrZW assembly.

Comparisons of BB or B males to OB, O or W morph females revealed elevated sex‐specific SNP density in the same chromosome interval across populations, suggesting a single evolutionary origin of the inversion. Some variation in sex‐specific SNP density within the inversion was observed among species. *M. callainos* Nkhata Bay showed a peak at 18.5 Mb that contrasted with average sex‐specific SNP density in the other two species at that location (Figure 4). Against LatrZW, the inverted region was from ~14.5 to 23.1 Mb with drops in sex‐specific SNP density from 15.1–15.7 Mb and 21.1–21.8 Mb (Figure 4). Closer inspection revealed that these decreases in sex‐specific SNP density were due to reduced alignment of pool‐seq reads against the LatrZW reference, likely because they corresponded with elevated TE density (Figure 5).

The populations of *M. callainos* at Luwino and Lupingu are mostly composed of white males and white females. The *F*_ST_ plots shows no detectable differentiation between white males and white females genome‐wide (Figure S5). Comparisons of these populations with BB individuals from other populations show overall high levels of Fst because they are interspecific comparisons, but there do not appear to be blocks of increased *F*_ST_ on LG5 that would suggest these white fish carry the inversion (Figure S5). To further investigate these populations, we genotyped mutations in a putative sox10 enhancer adjacent to *pax7a* which are characteristic of the OB alleles in other species/populations. The population of *M. callainos* at Luwino showed ~70% heterozygotes and ~30% homozygotes for the characteristic OB allele. The frequency of the OB allele is ~0.65 in both sexes (Table S2). We therefore expect a small number of BB homozygotes in this population. Although we did not collect or analyse any blue fish from Luwino, they are present at a low frequency. The population of *M. callainos* ‘Lupingu’ is composed entirely of white males and white females. Genotyping of the sox10 enhancer showed that both the males and females were homozygous for the characteristic OB mutation (Table S2). Thus, these two *M. callainos* populations have the pigment phenotype and the *pax7a* allele expected for OB individuals, but they do not appear to have the inversion or the associated sex locus. Further studies of these populations are needed to determine whether they represent an ancestral or derived state of the OB allele on LG5.

### 3.4 Allelic Diversity of Microsatellites

We calculated allele frequency distributions for three microsatellites within the inversion (Figures [Link], [Link]). The effective number of alleles (*N*_e_ = 1/∑ *p*_*i*_^2^) for *fibroleukin* ranged from 5.2 to 18.2, for *gata2* from 2.0 to 7.0, and for *pax7a* from 1.3 to 5.8. The highest diversity was observed for *M. callainos* Luwino, followed by *M. zebra* Nkhata Bay. The BB morphs typically showed the highest allelic diversity, reflecting their generally higher frequency in the population. The OB and O morphs frequently showed a high frequency of private alleles. For example, the OB and O individuals of *M. zebra* at Nkhata Bay have a high frequency of a 233 bp allele for *gata2* that is not observed in the BB individuals (Figure S7). This suggests the O alleles may have been recently derived from the OB alleles in this population. The 188 bp allele of pax7a is generally rare in BB morphs but is the most common allele in OB, O and W morphs, except for the *M. callainos* population at Luwino Reef, in which white males and females have a high frequency of 178 and 184 bp alleles (Figure S8).

### 3.5 Gene Expression in Gonads

In order to evaluate candidates for the sex locus within the inversion, we quantified gene expression in male and female gonads at the earliest developmental stage that dissection was practical (14–28 dpf). When we aligned RNAseq data from pooled OB female gonads and BB male gonads of *L. trewavasae* to the LatrZW inversion haplotype, we identified differential expression at four loci (Table 3; Table S3). One gene was more highly expressed in males and was similar to peroxisomal succinyl‐coenzyme A thioesterase‐like (*acot4‐like*). Three genes had elevated expression in the female gonad: Rho guanine nucleotide exchange factor 19 (*arhgef19*), Protein B4‐like *(b4‐like)* and GATA‐binding protein 2a (*gata2a*). Upregulation of *gata2a* was due to expression from the W allele (165 W reads vs. 15 Z reads). Examination of the inversion alleles for these genes found no amino acid changes in *gata2a*, a single conservative change in *b4‐like* and two non‐conservative changes and four deletions in *arhgef19*. We additionally examined *pax7a*, *mint* and *errfI1*, all candidate genes for colour, and found no changes to the amino acid sequence or differential expression in either, although gene expression differences impacting pigmentation may not be expected in gonadal tissue.

**TABLE 3:** Differential gene expression in gonads 14 days post fertilisation of *L. trewavasae* BB males versus OB females.

Gene name	Location (LG5) (CM105746.1)	ID	Male expression	Female expression	Log_2_ fold change	Amino acid substitutions
Peroxisomal succinyl‐coenzyme A thioesterase (*acot4*)	14,123,072–14,135,146	LOC102784311	6.6499	0.624551	−3.41244	No changes
Rho guanine nucleotide exchange factor 19 (*arhgef19*)	14,797,536–14,802,294	LOC101465733	0.512374	13.1674	4.68363	Two non‐conservative changes and 4 deletions
Protein B4‐like (*b4‐like*)	18,795,604–18,798,463	LOC101467966	0.896135	95.5427	6.73601	One conservative change
GATA binding protein 2 *(gata2a)*	20,574,659–20,583,240	LOC101470667	0.602127	11.8217	4.29523	No changes

To test for genes that may have more general sex‐associated expression differences, we also examined sex differences in expression across the inversion region in two cichlid species that do not segregate the inversion on LG5, and where sex is determined by loci on other chromosomes (Tables S4 and S5). The comparison of pooled expression data between *Aulonocara baenschi* male and female gonads showed differential expression in two genes within the inversion region: *b4‐like* (log_2_ fold change = 8.18496), and a locus that has partial identity to *poly(U)‐specific endoribonuclease like* (*endou*) (log_2_ fold change = −4.95394) (Table S4). The same comparison in *Oreochromis niloticus* featured no differentially expressed genes in the inverted region (Table S5).

The SNPeff analysis using GATK‐called variants identified heterozygous sequence differences of high effect in 12 genes (Table S6). These included frameshift variants in retinoic acid receptor gamma b (*rargb)*, inositol 1,4,5‐trisphosphate receptor type 1a (*itpr1a*), rheb‐like protein 1(*rhebl1*), protein kinase C delta (*prkcda*), coiled‐coil‐helix‐coiled‐coil‐helix domain containing 4b (*chchd4b*), Fanconi anaemia complementation group D2 (*fancd2*), cadherin EGF LAG seven‐pass G‐type receptor 2 (*celsr2*), myosin heavy chain b (*myhb*), potassium voltage‐gated channel subfamily A regulatory beta subunit 2a (*kcnab2a*) and taste 1 receptor member 3 (*tas1r3*). A frameshift was also called in a questionable annotation (ENSMZEG00005012252) which lies within an intron of the WNK lysine deficient protein kinase 2 (*wnk2*). An exon loss was called in one of several copies of interferon regulatory factor 6 (*irf6*). The long‐read variant caller svim identified five differences of high effect within the inversion (Table S7), but they were all homozygous, indicating they were not OB‐related but rather phylogenetic differences from the reference sequence.

## 4 Discussion

Our data demonstrate that alleles for a dominant female (W) sex locus and the dominant OB pigmentation phenotypes are linked within an ~7 Mb inversion on LG5 in several Lake Malawi cichlids. Here, we discuss some of the remaining questions about the origin and evolution of this supergene in the adaptive radiation in Lake Malawi.

### 4.1 Origin of the OB Inversion

Establishment of the inversion likely involved TEs, which are frequently implicated in genomic structural rearrangements including inversions (Bracewell et al. 2019; Gray 2000; Richards et al. 2005; Serrato‐Capuchina and Matute 2018). We found tandemly repeated copies of a CACTA transposon at both ends of the inversion. CACTA transposons are a Type II DNA element functioning via a cut‐and‐paste mechanism, which have been found in many vertebrates (Feschotte and Pritham 2007; Wells and Feschotte 2020). CACTA has previously been found at inversion breakpoints in rice, alongside TY3‐Gypsy, Ty1‐Copia and Mutator (Zhou et al. 2023). TEs may also have played a passive role in the inversion by providing templates for illegitimate recombination (Cáceres et al. 1999; Balachandran et al. 2022; Gozashti et al. 2024).

The mutational spectrum for inversions is likely skewed towards smaller inversions (Van Valen and Levins 1968; Gozashti et al. 2024). However, large inversions are more easily detected by many current techniques, and they may also be favoured by particular forms of selection, including sexually antagonistic selection acting on several adaptively favourable mutations linked to a sex locus (Connallon and Olito 2022). Evaluation of the patterns of selection on inversions and the importance of different sizes of inversions in the adaptive radiation await more unbiased surveys of inversion polymorphism.

### 4.2 Evolutionary History of the Inversion

The breakpoints of the inversion are consistent across species, which suggests a single origin for the inversion, although recurrent toggling of the inversion is possible (Porubsky et al. 2020). Within the inversion, gene order is conserved between the inverted and non‐inverted alleles, suggesting it was a simple inversion and not a series of overlapping inversions.

Several lines of evidence suggest that the inversion appeared early in the radiation of the rock‐dwelling ‘mbuna’ clade in Lake Malawi, which arose about 600,000 YBP (Schedel et al. 2019). First, OB phenotypes are found in multiple genera of Lake Malawi mbuna, including *Metriaclima* (*Maylandia*), *Labeotropheus*, *Tropheops* and *Genyochromis* (Roberts et al. 2009). Second, it is distributed in populations of many species throughout the lake (Figure 3). Third, differences in the structure of the inversion among populations also suggest a long period of independent evolution. The inversions in *L. trewavasae* and *M. zebra* show significant differences in overall length and in the pattern of TE accumulation (Figure 2). In particular, the differing peaks of TE density among these populations argues against a recent spread of the inversion between these two genera. However, distinguishing incomplete lineage sorting from introgression among genera will require characterisation of the OB haplotypes in additional genera.

The phenotypic appearance of OB varies within and among populations. Some individuals are heavily blotched, while others have few or no melanic spots. While some of this variation is undoubtedly due to differences in the genetic background, previous work has shown that the level of blotching is heritable within populations. The three distinct OB phenotypes of *L. trewavasae* at Thumbi West Island are maternally inherited (Roberts et al. 2017). This heritable variance in blotching suggests that multiple haplotypes of the inversion are segregating in this population.

There are two models for the order in which the OB mutation became associated with the W allele. In the first model, the novel sex locus appears first, and then the sexually antagonistic OB allele arises in close genetic linkage, enhancing the fitness of the new sex determiner. The alternative model is that the OB allele arose first, creating conditions that favoured the invasion of a new sex‐determining mutation. We have not yet found a population segregating a sex locus on LG5 in the absence of the OB allele. The high frequency of OB SNPs without sex‐linkage or an inversion in the *M. callainos* Luwino and Lupingu populations might be taken as evidence that the colour polymorphism arose first. However, taxonomically, this is a derived lineage which might have lost the sex locus and the inversion. High‐quality genome assemblies are needed for these populations to definitively determine whether the inversion is present, and genetic crosses should be performed to identify the sex loci segregating in each population.

### 4.3 Candidate Gene for Sex

The complete sequence of the inverted and non‐inverted haplotypes allows us to reconsider candidate genes for sex determination. We first note that the region does not contain any of the ‘usual suspects’ like *amh*, *dmrt* or *gsdf* (Bertho et al. 2021). Second, there are no obvious large deletions within this region. Third, there are no functionally significant amino acid replacements that appear likely to be responsible for sex.

Of the five differentially expressed genes in the inversion, only the transcription factor *gata2a* is thought to have a role in gonadal differentiation (Alexander et al. 2024). *gata2a* expression is approximately 13 times greater in ZW *L. trewavasae* females than in ZZ males (LOG_2_ FPKM ratio of 6.74). Expression of *gata2a* is low and equal in the male and female transcriptomes of 14 dpf *Aulonocara baenschi* and 28 dpf *Oreochromis niloticus* that have XY sex determination systems on chromosomes unlinked to the ZW locus on LG5 (Tables S4 and S5). Thus, it appears that high expression of the W‐linked *gata2a* allele in *L. trewavasae* is a derived trait. *Gata2a* has been studied mostly for its role in haematopoiesis (Vicente et al. 2012) and relatively little is known about its role in sex determination and gonad development. However, *gata2a* is strongly expressed in mouse ovaries, but not testes, during the critical period of sex determination from 11.5 to 15.5 days post copulation (Siggers et al. 2002). *Gata2* is also upregulated in the ovaries of the dragon lizard (Whiteley et al. 2022).

There are several mechanisms by which *gata2a* might exert a sex‐determining effect. The GATA motif is a conserved element of the proximal promoter for *gsdf* in *M. zebra* , as in other fishes (Gautier et al. 2011). Gata2 is a negative regulator of the TGFß pathway in mice (Dong et al. 2014; Billing et al. 2016). It is also possible that excess gata2a competes for autoregulatory sites in the *gata4* gene, thereby inhibiting the pathway of male development normally activated by Gata4/Fog2 (Lourenço et al. 2011; Mazaud‐Guittot et al. 2014). We could not identify sequence changes that might affect the expression of *gata2a*, but it is possible that its expression has been impacted by position effects of the inversion (Sharakhov and Sharakhova 2024). *Gata2* knockouts have been created in tilapia using CRISPR, resulting in hyperpigmentation and microphthalmia (Wang et al. 2021; Liu et al. 2023). XY‐*gata2a* ^−/−^ have a reduced gonadosomatic index and elevated estradiol levels consistent with a feminising effect. Normal folliculogenesis was observed in the XX *gata2a* ^−/−^, but they appear infertile, perhaps due to elevated levels of 11‐ketotestosterone (X. Liu, pers. comm.).

Finally, frameshift mutations in *rargb, prkcda* and *celsr2* might affect sexual development. *Rargb* and *Prkcda* are likely critical for spermatogenesis (Yang et al. 2021; Gely‐Pernot et al. 2012). *Celsr2* is critical for the adhesion of germ cells and Sertoli cells (Beall et al. 2005).

### 4.4 Candidate Genes for Colour

*Pax7a* has been suggested previously to be responsible for the OB phenotype (Roberts et al. 2009, 2017). Several lines of functional work have been conducted on *pax7*. In zebrafish, *pax7* is necessary to create the xanthophore lineage, and knockout of both *pax7a* and *pax7b* has developmental stage‐dependent impacts on melanophore number (Nord et al. 2016). In tilapia, it has been found that *pax7b* but not *pax7a* knockdown results in a loss of pigment (Wang et al. 2021). Finally, in goldfish, *pax7b* but not *pax7a* was shown to reduce the number of leucophores in scales (Mouri et al. 2024). Here, we found that *pax7a* has no coding changes that would result in loss of function. Previously, we found that the OB allele of Pax7 shows two‐fold higher expression than the BB allele (Roberts et al. 2009). Upregulation of Pax3/7 expression is correlated with a decrease in melanophore number in zebrafish (Minchin and Hughes 2008; Kubic et al. 2008; Lacosta et al. 2007) and an increase in xanthophore colouration in Lake Malawi cichlids (Albertson et al. 2014).

We previously showed that the density of blotching is inherited matrilineally as discrete morphs in *Labeotropheus trewavasae* , suggesting that variation among inversion haplotypes is responsible for this variation (Roberts et al. 2017). It is possible that more than one gene is influencing the colour phenotype, and that allelic variation at more than one gene within the inversion haplotype influences pigmentation. CRISPR knockouts of *gata2a* in tilapia cause a hyperpigmentation phenotype (Wang et al. 2021; Liu et al. 2023). We observed an upregulation of *gata2a* from the OB/W haplotype in gonads of *L. trewavasae* . Changes in *gata2a* expression could be responsible for hypopigmentation of melanophores in OB fish. Another candidate for colour is *msx2‐interacting protein* (*mint*). *Mint* represses transcription in the Notch signalling pathway, which is responsible for cell fate decisions (VanderWielen et al. 2011). Notch signalling has been previously shown to impact stripe formation in zebrafish, as melanophores die in the absence of Delta/Notch signalling (Hamada et al. 2014). However, we found no coding or gonadal expression differences among the cichlid colour morphs. Finally, *Erbb receptor feedback inhibitor 1* (*errfi1*) might also contribute to the OB phenotype. *Errfi1* acts as an inhibitor of the epidermal growth factor/erbB receptor family which has been implicated in pigmentation in mammals and fish (Fitch et al. 2003; Budi et al. 2008). *Errfi1* has been directly implicated in hair colour variation in humans (Morgan et al. 2018). Notably, genetic and pharmaceutical inhibition of erbB receptors in Danio zebrafish results in local breakdown of regularly patterned melanophore stripes into disorganised spots (Dooley et al. 2013), phenotypically analogous to melanophore clustering following breakdown of barred patterning in OB cichlids (Roberts et al. 2017). Studies in zebrafish also demonstrate that larval melanophores are erbB‐independent, but juvenile and adult melanophore differentiation is perturbed by erbB inhibition (Hultman and Johnson 2010). Similarly, most OB cichlid morph pigmentation differences arise during larval to juvenile development, though one particular OB morph was also shown to lack larval melanophores (Roberts et al. 2017). However, we found no coding or gonadal expression changes in *errfi1*.

### 4.5 Male OB

The OB phenotype has previously been considered to be rare in wild males, as the blotching disrupts male nuptial patterning and thus likely reduces mating success (Roberts et al. 2009). However, informal surveys have revealed high frequencies (> 1%) of OB males at a few localities around the lake (Figure 3). The OB male sequenced in this study is derived from Masinje, a population on the southeastern shore of Lake Malawi that has a high frequency of OB males. It is likely that in these populations there are at least some matings between heterozygous OB males and heterozygous OB females. OB homozygotes produced in the lab are viable, so it is likely that OB homozygotes also exist in the wild. Thus, there may be a limited opportunity for recombination among inverted alleles in some populations.

There are several evolutionary/genetic pathways that could produce OB males. The first possibility is that OB arose first, and the ZW system has arisen more recently in only some populations. In this scenario, the population at Masinje would not carry the ZW locus in the inversion, and OB would not be sex‐linked. The second possibility is that the ancestral state was an inversion that included both the OB and W alleles, but there have been multiple losses of the ZW system. A third possibility is that the OB variant spread to the non‐inverted haplotype through inter‐karyotypic introgression (Grau‐Bové et al. 2020). We prefer the idea that populations with a high frequency of OB males are segregating an unknown male sex determiner that is epistatically dominant to the W linked to OB. *Metriaclima* populations are already known to be segregating at least three sex loci. The ZW locus located in the inversion on LG5 is epistatically dominant to an XY system on LG7 (Ser et al. 2010). A B chromosome carrying a dominant female (W) determiner is also epistatically dominant to the XY system on LG7 (Clark and Kocher 2019). The presence of multiple dominant female systems in a population might select for the invasion of a new, epistatically dominant male determiner to restore an even sex ratio. In a previous study, we obtained broods sired by two OB male M. ‘kompakt’ males. Their OB alleles remained tightly associated with female sex in their offspring (Roberts et al. 2009), suggesting that genetic or environmental factors can override the LG5 ZW system in some individuals. Experiments to map sex determiners in the Masinje population are in progress.

### 4.6 Structural Variants in Adaptive Radiation

The development of relatively inexpensive technologies for assembling highly contiguous genome sequences has renewed interest in the role of structural variants in adaptive evolution (Mérot et al. 2020). Recent work has identified several large structural variants associated with the radiation of cichlids in Lake Malawi. One study identified five inversions on chromosomes 2, 9, 10, 11 and 13, segregating in the benthic sub‐radiation (Blumer et al. 2024). A second study confirmed these five inversions and identified a sixth inversion on chromosome 20 using a combination of long‐read sequencing and Bionano physical maps (Kumar et al. 2024). These inversions are large, ranging from 10 to 23 Mb in length and representing 25%–55% of the corresponding chromosomes. Both studies presented evidence that the inversions on Chr9, Chr10 and Chr11 are sex‐linked in extant populations. They further suggest that the inversions carry variants that have been important in sensory and behavioural adaptations during the radiation.

The inversion on LG5 that we have characterised here links a dominant female sex determiner (W) with orange/white pigment variants. OB pigmentation likely provides benefits of crypsis to individuals on the mottled background of algae‐covered rocks, while white pigmentation may provide crypsis against the background space light when fish are feeding in the water column. Linkage to the female (W) sex determiner allows females to benefit from this crypsis, while allowing ZZ males to retain their species‐characteristic nuptial colours. Matrilineal inheritance of OB morphs within populations suggests that multiple mutations, possibly in several different genes, may have accumulated within the inversion. Likewise, sexually antagonistic selection may have acted to alter the sequence or expression of additional genes in the inversion. The assembly of the complete haplotype required a minimum of three mutations: one affecting pigmentation (*pax7a*), one affecting sex determination and the structural mutation that created the inversion. We have not yet discovered a population that would allow us to determine the order in which these mutations arose.

The spectacular radiations of cichlid fishes in the lakes of East Africa represent both an enduring evolutionary mystery and an opportunity to understand the mechanisms of adaptation and speciation. The unexpected diversity of sex chromosomes in these fishes has introduced new perspectives on these questions. Turnover of sex chromosome systems may facilitate the invasion of large inversions carrying both a sex locus and other adaptive variants. Fixation of such sex‐linked supergenes may be associated with many of the significant evolutionary transitions in these radiations. We suggest the OB inversion could be a useful model system for understanding the role of structural variants in adaptive radiation. The ecologically important pigmentation phenotype is easily accessible for field studies in shallow water. The many replicate populations, with varying frequencies of OB and OB males, offer an opportunity to study the evolution of this polygenic sex determining system. Turnover of sex chromosomes may play an important role in this adaptive radiation.

## Author Contributions

T.D.K. conceived and designed the study. T.D.K., R.B.R., N.M.K. and A.F.K. performed field and/or laboratory work. K.A.B., S.J., M.A.C. and A.C.M. analysed the sequence data. K.A.B. and T.D.K. interpreted the data and wrote the paper. N.M.K., J.T.S., R.B.R. and P.T.M. critically reviewed and edited the manuscript. All the authors approved the final version of the manuscript.

## Conflicts of Interest

The authors declare no conflicts of interest.

## Supporting information


Figure S1.

Figure S2.

Figure S3.

Figure S4.

Figure S5.

Figure S6.

Figure S7.

Figure S8.

Table S1.

Table S2.

Table S3.

Table S4.

Table S5.

Table S6.

Table S7.

## Data Availability

Raw sequence reads and genome assemblies have been deposited in GenBank under Bioproject PRJNA1181016. Sequence reads from pooled whole‐genome analyses have been deposited in the Sequence Read Archive (https://www.ncbi.nlm.nih.gov/sra) under Bioproject PRJNA808791. RNAseq data has been deposited in GenBank under Bioproject PRJNA1196390.

## References

[mec17821-bib-0001] Albertson, R. C. , K. E. Powder , Y. Hu , K. P. Coyle , R. B. Roberts , and K. J. Parsons . 2014. “Genetic Basis of Continuous Variation in the Levels and Modular Inheritance of Pigmentation in Cichlid Fishes.” Molecular Ecology 23: 5135–5150. 25156298 10.1111/mec.12900 PMC4238941

[mec17821-bib-0002] Alexander, A. K. , K. F. Rodriguez , Y.‐Y. Chen , et al. 2024. “Single‐Nucleus Multiomics Reveals the Gene‐Regulatory Networks Underlying Sex Determination of Murine Primordial Germ Cells.” eLife 13: RP96591. 10.7554/eLife.96591.1. PMC11893106 40063068

[mec17821-bib-0003] Ayala, D. , R. F. Guerrero , and M. Kirkpatrick . 2013. “Reproductive Isolation and Local Adaptation Quantified for a Chromosome Inversion in a Malaria Mosquito.” Evolution; International Journal of Organic Evolution 67, no. 4: 946–958. 10.1111/j.1558-5646.2012.01836.x. 23550747

[mec17821-bib-0004] Balachandran, P. , I. A. Walawalkar , J. I. Flores , J. N. Dayton , P. A. Audano , and C. R. Beck . 2022. “Transposable Element‐Mediated Rearrangements Are Prevalent in Human Genomes.” Nature Communications 13, no. 1: 7115. 10.1038/s41467-022-34810-8. PMC9675761 36402840

[mec17821-bib-0005] Beall, S. A. , K. Boekelheide , and K. J. Johnson . 2005. “Hybrid GPCR/Cadherin (Celsr) Proteins in Rat Testis Are Expressed With Cell Type Specificity and Exhibit Differential Sertoli Cell‐Germ Cell Adhesion Activity.” Journal of Andrology 26, no. 4: 529–538. 10.2164/jandrol.05003. 15955893

[mec17821-bib-0006] Behrens, K. A. , S. Koblmüller , and T. D. Kocher . 2022. “Sex Chromosomes in the Tribe Cyprichromini (Teleostei: Cichlidae) of Lake Tanganyika.” Scientific Reports 12, no. 1: 1–13. 10.1038/s41598-022-23017-y. 36289404 PMC9606112

[mec17821-bib-0007] Behrens, K. A. , S. Koblmüller , and T. D. Kocher . 2024. “Diversity of Sex Chromosomes in Vertebrates: Six Novel Sex Chromosomes in Basal Haplochromines (Teleostei: Cichlidae).” Genome Biology and Evolution 16, no. 7: evae152. 10.1093/gbe/evae152. 39073759 PMC11285159

[mec17821-bib-0008] Behrens, K. A. , H. Zimmermann , R. Blažek , M. Reichard , S. Koblmüller , and T. D. Kocher . 2024. “Turnover of Sex Chromosomes in the Lake Tanganyika Cichlid Tribe Tropheini (Teleostei: Cichlidae).” Scientific Reports 14, no. 1: 2471. 10.1038/s41598-024-53021-3. 38291228 PMC10828463

[mec17821-bib-0009] Berdan, E. L. , N. H. Barton , R. Butlin , et al. 2023. “How Chromosomal Inversions Reorient the Evolutionary Process.” Journal of Evolutionary Biology 36, no. 12: 1761–1782. 10.1111/jeb.14242. 37942504

[mec17821-bib-0010] Berdan, E. L. , A. Blanckaert , R. K. Butlin , and C. Bank . 2021. “Deleterious Mutation Accumulation and the Long‐Term Fate of Chromosomal Inversions.” PLoS Genetics 17, no. 3: e1009411. 10.1371/journal.pgen.1009411. 33661924 PMC7963061

[mec17821-bib-0011] Bergero, R. , and D. Charlesworth . 2009. “The Evolution of Restricted Recombination in Sex Chromosomes.” Trends in Ecology & Evolution 24, no. 2: 94–102. 10.1016/j.tree.2008.09.010. 19100654

[mec17821-bib-0012] Bertho, S. , A. Herpin , M. Schartl , and Y. Guiguen . 2021. “Lessons From an Unusual Vertebrate Sex‐Determining Gene.” Philosophical Transactions of the Royal Society of London. Series B, Biological Sciences 376, no. 1832: 20200092. 10.1098/rstb.2020.0092. 34247499 PMC8273500

[mec17821-bib-0013] Billing, M. , E. Rörby , G. May , et al. 2016. “A Network Including TGFβ/Smad4, Gata2, and p57 Regulates Proliferation of Mouse Hematopoietic Progenitor Cells.” Experimental Hematology 44, no. 5: 399–409.e5. 10.1016/j.exphem.2016.02.001. 26876150

[mec17821-bib-0014] Black, D. , and D. M. Shuker . 2019. “Supergenes.” Current Biology 29, no. 13: R615–R617. 10.1016/j.cub.2019.05.024. 31287973

[mec17821-bib-0015] Blumer, L. M. , V. Burskaia , I. Artiushin , et al. 2024. “Introgression Dynamics of Sex‐Linked Chromosomal Inversions Shape the Malawi Cichlid Adaptive Radiation.” *bioRxiv*. 10.1101/2024.07.28.605452. PMC7617772 40504893

[mec17821-bib-0016] Bracewell, R. , K. Chatla , M. J. Nalley , and D. Bachtrog . 2019. “Dynamic Turnover of Centromeres Drives Karyotype Evolution in Drosophila.” eLife 8: e49002. 10.7554/eLife.49002. 31524597 PMC6795482

[mec17821-bib-0017] Budi, E. H. , L. B. Patterson , and D. M. Parichy . 2008. “Embryonic Requirements for ErbB Signaling in Neural Crest Development and Adult Pigment Pattern Formation.” Development 135, no. 15: 2603–2614. 10.1242/dev.019299. 18508863 PMC2704560

[mec17821-bib-0018] Burress, E. D. 2015. “Cichlid Fishes as Models of Ecological Diversification: Patterns, Mechanisms, and Consequences.” Hydrobiologia 748, no. 1: 7–27. 10.1007/s10750-014-1960-z.

[mec17821-bib-0019] Cabanettes, F. , and C. Klopp . 2018. “D‐GENIES: Dot Plot Large Genomes in an Interactive, Efficient and Simple Way.” PeerJ 6: e4958. 10.7717/peerj.4958. 29888139 PMC5991294

[mec17821-bib-0020] Cáceres, M. , J. M. Ranz , A. Barbadilla , M. Long , and A. Ruiz . 1999. “Generation of a Widespread Drosophila Inversion by a Transposable Element.” Science 285, no. 5426: 415–418. 10.1126/science.285.5426.415. 10411506

[mec17821-bib-0021] Cantalapiedra, C. P. , A. Hernández‐Plaza , I. Letunic , P. Bork , and J. Huerta‐Cepas . 2021. “eggNOG‐Mapper v2: Functional Annotation, Orthology Assignments, and Domain Prediction at the Metagenomic Scale.” Molecular Biology and Evolution 38, no. 12: 5825–5829. 10.1093/molbev/msab293. 34597405 PMC8662613

[mec17821-bib-0022] Charlesworth, D. , and A. Harkess . 2024. “Why Should We Study Plant Sex Chromosomes?” Plant Cell 36, no. 5: 1242–1256. 10.1093/plcell/koad278. 38163640 PMC11062472

[mec17821-bib-0023] Cheng, H. , G. T. Concepcion , X. Feng , H. Zhang , and H. Li . 2021. “Haplotype‐Resolved de Novo Assembly Using Phased Assembly Graphs With Hifiasm.” Nature Methods 18, no. 2: 170–175. 10.1038/s41592-020-01056-5. 33526886 PMC7961889

[mec17821-bib-0024] Cingolani, P. , A. Platts , L. L. Wang , et al. 2012. “A Program for Annotating and Predicting the Effects of Single Nucleotide Polymorphisms, SnpEff: SNPs in the Genome of *Drosophila melanogaster* Strain w1118; Iso‐2; Iso‐3.” Fly 6, no. 2: 80–92. 10.4161/fly.19695. 22728672 PMC3679285

[mec17821-bib-0025] Clark, F. E. , and T. D. Kocher . 2019. “Changing Sex for Selfish Gain: B Chromosomes of Lake Malawi Cichlid Fish.” Scientific Reports 9, no. 1: 20213. 10.1038/s41598-019-55774-8. 31882583 PMC6934658

[mec17821-bib-0026] Connallon, T. , and C. Olito . 2022. “Natural Selection and the Distribution of Chromosomal Inversion Lengths.” Molecular Ecology 31, no. 13: 3627–3641. 10.1111/mec.16091. 34297880

[mec17821-bib-0027] Conte, M. A. , R. Joshi , E. C. Moore , et al. 2019. “Chromosome‐Scale Assemblies Reveal the Structural Evolution of African Cichlid Genomes.” GigaScience 8, no. 4: giz030. 10.1093/gigascience/giz030. 30942871 PMC6447674

[mec17821-bib-0028] Cortez, D. , R. Marin , D. Toledo‐Flores , et al. 2014. “Origins and Functional Evolution of y Chromosomes Across Mammals.” Nature 508, no. 7497: 488–493. 10.1038/nature13151. 24759410

[mec17821-bib-0029] Danecek, P. , J. K. Bonfield , J. Liddle , et al. 2021. “Twelve Years of SAMtools and BCFtools.” GigaScience 10, no. 2: giab008. 10.1093/gigascience/giab008. 33590861 PMC7931819

[mec17821-bib-0030] Dobzhansky, T. 1971. “Evolutionary Oscillations in *Drosophila pseudoobscura* .” In Ecological Genetics and Evolution, edited by R. Creed , 109–133. Blackwell Scientific Publications.

[mec17821-bib-0031] Dong, L. , W. W. Zheng , L. J. Tang , et al. 2014. “GATA‐2 Inhibits Transforming Growth Factor‐β Signaling Pathway Through Interaction With Smad4.” Cellular Signalling 26, no. 5: 1089–1097. 10.1016/j.cellsig.2014.01.028. 24509415

[mec17821-bib-0032] Dooley, C. M. , A. Mongera , B. Walderich , and C. Nüsslein‐Volhard . 2013. “On the Embryonic Origin of Adult Melanophores: The Role of ErbB and Kit Signalling in Establishing Melanophore Stem Cells in Zebrafish.” Development 140, no. 5: 1003–1013. 10.1242/dev.087007. 23364329

[mec17821-bib-0033] El Taher, A. , F. Ronco , M. Matschiner , W. Salzburger , and A. Böhne . 2021. “Dynamics of Sex Chromosome Evolution in a Rapid Radiation of Cichlid Fishes.” Science Advances 7, no. 36: eabe8215. 10.1126/sciadv.abe8215. 34516923 PMC8442896

[mec17821-bib-0034] Feschotte, C. , and E. J. Pritham . 2007. “DNA Transposons and the Evolution of Eukaryotic Genomes.” Annual Review of Genetics 41: 331–368. 10.1146/annurev.genet.40.110405.090448. PMC2167627 18076328

[mec17821-bib-0035] Fitch, K. R. , K. A. McGowan , C. D. van Raamsdonk , et al. 2003. “Genetics of Dark Skin in Mice.” Genes & Development 17, no. 2: 214–228. 10.1101/gad.1023703. 12533510 PMC195979

[mec17821-bib-0036] Gabriel, L. , T. Brůna , K. J. Hoff , et al. 2024. “BRAKER3: Fully Automated Genome Annotation Using RNA‐Seq and Protein Evidence With GeneMark‐ETP, AUGUSTUS, and TSEBRA.” Genome Research 34, no. 5: 769–777. 10.1101/gr.278090.123. 38866550 PMC11216308

[mec17821-bib-0037] Gammerdinger, W. J. , M. A. Conte , B. A. Sandkam , A. Ziegelbecker , S. Koblmüller , and T. D. Kocher . 2018. “Novel Sex Chromosomes in 3 Cichlid Fishes From Lake Tanganyika.” Journal of Heredity 109, no. 5: 489–500. 10.1093/jhered/esy003. 29444291

[mec17821-bib-0038] Gautier, A. , F. Sohm , J. S. Joly , F. Le Gac , and J. J. Lareyre . 2011. “The Proximal Promoter Region of the Zebrafish Gsdf Gene Is Sufficient to Mimic the Spatio‐Temporal Expression Pattern of the Endogenous Gene in Sertoli and Granulosa Cells.” Biology of Reproduction 85, no. 6: 1240–1251. 10.1095/biolreprod.111.091892. 21816849

[mec17821-bib-0039] Gely‐Pernot, A. , M. Raverdeau , C. Célébi , et al. 2012. “Spermatogonia Differentiation Requires Retinoic Acid Receptor γ.” Endocrinology 153, no. 1: 438–449. 10.1210/en.2011-1102. 22045663

[mec17821-bib-0040] Gozashti, L. , O. S. Harringmeyer , and H. E. Hoekstra . 2024. “How Repeats Rearrange Chromosomes in Deer Mice.” *bioRiv*. 10.1101/2024.05.29.596518. 40327505

[mec17821-bib-0041] Grau‐Bové, X. , S. Tomlinson , A. O. O'Reilly , et al. 2020. “Evolution of the Insecticide Target Rdl in African Anopheles Is Driven by Interspecific and Interkaryotypic Introgression.” Molecular Biology and Evolution 37, no. 10: 2900–2917. 10.1093/molbev/msaa128. 32449755 PMC7530614

[mec17821-bib-0042] Gray, Y. H. M. 2000. “It Takes Two Transposons to Tango:Transposable‐Element‐Mediated Chromosomal Rearrangements.” Trends in Genetics 16, no. 10: 461–468. 10.1016/S0168-9525(00)02104-1. 11050333

[mec17821-bib-0043] Hamada, H. , M. Watanabe , H. E. Lau , et al. 2014. “Involvement of Delta/Notch Signaling in Zebrafish Adult Pigment Stripe Patterning.” Development 141, no. 2: 318–324. 10.1242/dev.099804. 24306107 PMC3879813

[mec17821-bib-0044] Heller, D. , and M. Vingron . 2019. “SVIM: Structural Variant Identification Using Mapped Long Reads.” Bioinformatics 35, no. 17: 2907–2915. 10.1093/bioinformatics/btz041. 30668829 PMC6735718

[mec17821-bib-0045] Hultman, K. A. , and S. L. Johnson . 2010. “Differential Contribution of Direct‐Developing and Stem Cell‐Derived Melanocytes to the Zebrafish Larval Pigment Pattern.” Developmental Biology 337, no. 2: 425–431. 10.1016/j.ydbio.2009.11.019. 19931238 PMC2812685

[mec17821-bib-0046] Institute for Laboratory Animal Research , et al. 2011. Guide for the Care and Use of Laboratory Animals. 8th ed. National Academies Press.

[mec17821-bib-0047] Jamsandekar, M. , M. S. Ferreira , M. E. Pettersson , E. D. Farrell , B. W. Davis , and L. Andersson . 2024. “The Origin and Maintenance of Supergenes Contributing to Ecological Adaptation in Atlantic Herring.” Nature Communications 15, no. 1: 9136. 10.1038/s41467-024-53079-7. PMC11499932 39443489

[mec17821-bib-0048] Jay, P. , A. Whibley , L. Frézal , et al. 2018. “Supergene Evolution Triggered by the Introgression of a Chromosomal Inversion.” Current Biology 28, no. 11: 1839–1845.e3. 10.1016/j.cub.2018.04.072. 29804810

[mec17821-bib-0049] Kamiya, T. , W. Kai , S. Tasumi , et al. 2012. “A Trans‐Species Missense SNP in Amhr2 Is Associated With Sex Determination in the Tiger Pufferfish, *Takifugu rubripes* (Fugu).” PLoS Genetics 8, no. 7: e1002798. 10.1371/journal.pgen.1002798. 22807687 PMC3395601

[mec17821-bib-0050] Kim, D. , J. M. Paggi , C. Park , C. Bennett , and S. L. Salzberg . 2019. “Graph‐Based Genome Alignment and Genotyping With HISAT2 and HISAT‐Genotype.” Nature Biotechnology 37, no. 8: 907–915. 10.1038/s41587-019-0201-4. PMC7605509 31375807

[mec17821-bib-0051] Kirkpatrick, M. 2010. “How and Why Chromosome Inversions Evolve.” PLoS Biology 8, no. 9: e1000501. 10.1371/journal.pbio.1000501. 20927412 PMC2946949

[mec17821-bib-0052] Kirkpatrick, M. , and N. Barton . 2006. “Chromosome Inversions, Local Adaptation and Speciation.” Genetics 173, no. 1: 419–434. 10.1534/genetics.105.047985. 16204214 PMC1461441

[mec17821-bib-0053] Kitano, J. , S. Ansai , Y. Takehana , and Y. Yamamoto . 2024. “Diversity and Convergence of Sex‐Determination Mechanisms in Teleost Fish.” Annual Review of Animal Biosciences 12: 233–259. 10.1146/annurev-animal-021122-113935. 37863090

[mec17821-bib-0054] Knief, U. , I. A. Müller , K. F. Stryjewski , D. Metzler , M. D. Sorenson , and J. B. W. Wolf . 2024. “Evolution of Chromosomal Inversions Across an Avian Radiation.” Molecular Biology and Evolution 41, no. 6: msae092. 10.1093/molbev/msae092. 38743589 PMC11152452

[mec17821-bib-0055] Kocher, T. D. , W. K. Thomas , A. Meyer , et al. 1989. “Dynamics of Mitochondrial DNA Evolution in Animals: Amplification and Sequencing With Conserved Primers.” Proceedings of the National Academy of Sciences of the United States of America 86, no. 16: 6196–6200. 10.1073/pnas.86.16.6196. 2762322 PMC297804

[mec17821-bib-0056] Kofler, R. , R. V. Pandey , and C. Schloetterer . 2011. “PoPoolation2: Identifying Differentiation Between Populations Using Sequencing of Pooled DNA Samples (Pool‐Seq).” Bioinformatics 27: 3435–3436. 10.1093/bioinformatics/btr589. 22025480 PMC3232374

[mec17821-bib-0057] Konigs, A. 2001. Malawi Cichlids in Their Natural Habitat. Cichlid Press.

[mec17821-bib-0058] Kubic, J. D. , K. P. Young , R. S. Plummer , A. E. Ludvik , and D. Lang . 2008. “Pigmentation PAX‐Ways: The Role of Pax3 in Melanogenesis, Melanocyte Stem Cell Maintenance, and Disease.” Pigment Cell & Melanoma Research 21: 627–645. 18983540 10.1111/j.1755-148X.2008.00514.x PMC2979299

[mec17821-bib-0059] Kumar, N. K. , T. L. Cooper , T. D. Kocher , J. T. Streelman , and P. T. McGrath . 2024. “Large Inversions in Lake Malawi Cichlids Are Associated With Habitat Preference, Lineage, and Sex Determination.” *bioRxiv*. 10.1101/2024.10.28.620687.

[mec17821-bib-0060] Lacosta, A. M. , J. Canudas , C. Gonzalez , P. Muniesa , M. Sarasa , and L. Dominguez . 2007. “Pax7 Identifies Neural Crest, Chromatophore Lineages and Pigment Stem Cells During Zebrafish Development.” International Journal of Developmental Biology 51: 327–331. 17554685 10.1387/ijdb.062217al

[mec17821-bib-0061] Li, H. 2018. “Minimap2: Pairwise Alignment for Nucleotide Sequences.” Bioinformatics 34, no. 18: 3094–3100. 10.1093/bioinformatics/bty191. 29750242 PMC6137996

[mec17821-bib-0062] Liu, X. , L. Zhou , W. Li , J. Wu , and D. Wang . 2023. “Gata2a Mutation Causes Progressive Microphthalmia and Blindness in Nile Tilapia.” International Journal of Molecular Sciences 24, no. 4: 3567. 10.3390/ijms24043567. 36834978 PMC9958714

[mec17821-bib-0063] Lourenço, D. , R. Brauner , M. Rybczyńska , C. Nihoul‐Fékété , K. McElreavey , and A. Bashamboo . 2011. “Loss‐Of‐Function Mutation in GATA4 Causes Anomalies of Human Testicular Development.” Proceedings of the National Academy of Sciences 108, no. 4: 1597–1602. 10.1073/pnas.1010257108. PMC3029689 21220346

[mec17821-bib-0064] Lowry, D. B. , and J. H. Willis . 2010. “A Widespread Chromosomal Inversion Polymorphism Contributes to a Major Life‐History Transition, Local Adaptation, and Reproductive Isolation.” PLoS Biology 8, no. 9: e1000500. 10.1371/journal.pbio.1000500. 20927411 PMC2946948

[mec17821-bib-0065] MacGuigan, D. J. , T. J. Krabbenhoft , R. C. Harrington , D. K. Wainwright , N. J. C. Backenstose , and T. J. Near . 2023. “Lacustrine Speciation Associated With Chromosomal Inversion in a Lineage of Riverine Fishes.” Evolution 77, no. 7: 1505–1521. 10.1093/evolut/qpad067. 37094800 PMC10309973

[mec17821-bib-0066] Madeira, F. , M. Pearce , A. R. N. Tivey , et al. 2022. “Search and Sequence Analysis Tools Services From EMBL‐EBI in 2022.” Nucleic Acids Research 50, no. W1: W276–W279. 10.1093/nar/gkac240. 35412617 PMC9252731

[mec17821-bib-0067] Manni, M. , M. R. Berkeley , M. Seppey , F. A. Simão , and E. M. Zdobnov . 2021. “BUSCO Update: Novel and Streamlined Workflows Along With Broader and Deeper Phylogenetic Coverage for Scoring of Eukaryotic, Prokaryotic, and Viral Genomes.” Molecular Biology and Evolution 38, no. 10: 4647–4654. 10.1093/molbev/msab199. 34320186 PMC8476166

[mec17821-bib-0068] Marçais, G. , A. L. Delcher , A. M. Phillippy , R. Coston , S. L. Salzberg , and A. Zimin . 2018. “MUMmer4: A Fast and Versatile Genome Alignment System.” PLoS Computational Biology 14, no. 1: e1005944. 10.1371/journal.pcbi.1005944. 29373581 PMC5802927

[mec17821-bib-0069] Matschiner, M. , J. M. I. Barth , O. K. Tørresen , et al. 2022. “Supergene Origin and Maintenance in Atlantic Cod.” Nature Ecology & Evolution 6, no. 4: 469–481. 10.1038/s41559-022-01661-x. 35177802 PMC8986531

[mec17821-bib-0070] Mazaud‐Guittot, S. , B. Prud'homme , M. F. Bouchard , et al. 2014. “GATA4 Autoregulates Its Own Expression in Mouse Gonadal Cells via Its Distal 1b Promoter.” Biology of Reproduction 90, no. 2: 25. 10.1095/biolreprod.113.113290. 24352556

[mec17821-bib-0071] Mérot, C. , V. Llaurens , E. Normandeau , L. Bernatchez , and M. Wellenreuther . 2020. “Balancing Selection via Life‐History Trade‐Offs Maintains an Inversion Polymorphism in a Seaweed Fly.” Nature Communications 11, no. 1: 670. 10.1038/s41467-020-14479-7. PMC6997199 32015341

[mec17821-bib-0072] Minchin, J. E. , and S. M. Hughes . 2008. “Sequential Actions of Pax3 and Pax7 Drive Xanthophore Development in Zebrafish Neural Crest.” Developmental Biology 317: 508–522. 18417109 10.1016/j.ydbio.2008.02.058 PMC3005709

[mec17821-bib-0073] Moore, E. C. , P. J. Ciccotto , E. N. Peterson , M. S. Lamm , R. C. Albertson , and R. B. Roberts . 2022. “Polygenic Sex Determination Produces Modular Sex Polymorphism in an African Cichlid Fish.” Proceedings of the National Academy of Sciences of the United States of America 119, no. 14: e2118574119. 10.1073/pnas.2118574119. 35357968 PMC9168840

[mec17821-bib-0074] Morgan, M. D. , E. Pairo‐Castineira , K. Rawlik , et al. 2018. “Genome‐Wide Study of Hair Colour in UK Biobank Explains Most of the SNP Heritability.” Nature Communications 9, no. 1: 5271. 10.1038/s41467-018-07691-z. PMC6288091 30531825

[mec17821-bib-0075] Mouri, T. , S. Usa , and T. Tokumoto . 2024. “Pax7 Is Involved in Leucophore Formation in Goldfish and Gene Knockout Improves the Transparency of Transparent Goldfish.” Fish Physiology and Biochemistry 50, no. 4: 1701–1710. 10.1007/s10695-024-01364-z. 38819758

[mec17821-bib-0076] Myosho, T. , Y. Takehana , S. Hamaguchi , and M. Sakaizumi . 2015. “Turnover of Sex Chromosomes in Celebensis Group Medaka Fishes.” G3 (Bethesda, Md.) 5, no. 12: 2685–2691. 10.1534/g3.115.021543. 26497145 PMC4683641

[mec17821-bib-0077] Nei, M. 1973. “Analysis of Gene Diversity in Subdivided Populations.” Proceedings of the National Academy of Sciences of the United States of America 70, no. 12: 3321–3323. 10.1073/pnas.70.12.3321. 4519626 PMC427228

[mec17821-bib-0078] Nord, H. , N. Dennhag , J. Muck , and J. von Hofsten . 2016. “Pax7 Is Required for Establishment of the Xanthophore Lineage in Zebrafish Embryos.” Molecular Biology of the Cell 27, no. 11: 1853–1862. 10.1091/mbc.e15-12-0821. 27053658 PMC4884075

[mec17821-bib-0079] Olito, C. , S. Ponnikas , B. Hansson , and J. K. Abbott . 2024. “Consequences of Partially Recessive Deleterious Genetic Variation for the Evolution of Inversions Suppressing Recombination Between Sex Chromosomes.” Evolution 76, no. 6: 1320–1330. 10.1093/evolut/qpae060. PMC9324078 35482933

[mec17821-bib-0080] Ou, S. , W. Su , Y. Liao , et al. 2019. “Benchmarking Transposable Element Annotation Methods for Creation of a Streamlined, Comprehensive Pipeline.” Genome Biology 20, no. 1: 275. 10.1186/s13059-019-1905-y. 31843001 PMC6913007

[mec17821-bib-0081] Porubsky, D. , A. D. Sanders , W. Höps , et al. 2020. “Recurrent Inversion Toggling and Great Ape Genome Evolution.” Nature Genetics 52, no. 8: 849–858. 10.1038/s41588-020-0646-x. 32541924 PMC7415573

[mec17821-bib-0082] Quigley, S. , J. Damas , D. M. Larkin , and M. Farré . 2023. “syntenyPlotteR: A User‐Friendly R Package to Visualize Genome Synteny, Ideal for Both Experienced and Novice Bioinformaticians.” Bioinformatics Advances 3, no. 1: vbad161. 10.1093/bioadv/vbad161. 38023328 PMC10660287

[mec17821-bib-0083] Rhie, A. , B. P. Walenz , S. Koren , and A. M. Phillippy . 2020. “Merqury: Reference‐Free Quality, Completeness, and Phasing Assessment for Genome Assemblies.” Genome Biology 21, no. 1: 245. 10.1186/s13059-020-02134-9. 32928274 PMC7488777

[mec17821-bib-0084] Rice, W. R. 1992. “Sexually Antagonistic Genes: Experimental Evidence.” Science 256, no. 5062: 1436–1439. 10.1126/science.1604317. 1604317

[mec17821-bib-0085] Richards, S. , Y. Liu , B. R. Bettencourt , et al. 2005. “Comparative Genome Sequencing of *Drosophila pseudoobscura*: Chromosomal, Gene, and Cis‐Element Evolution.” Genome Research 15, no. 1: 1–18. 10.1101/gr.3059305. 15632085 PMC540289

[mec17821-bib-0086] Roberts, R. B. , E. C. Moore , and T. D. Kocher . 2017. “An Allelic Series at pax7a Is Associated With Colour Polymorphism Diversity in Lake Malawi Cichlid Fish.” Molecular Ecology 26, no. 10: 2625–2639. 10.1111/mec.13975. 28027432 PMC5425315

[mec17821-bib-0087] Roberts, R. B. , J. R. Ser , and T. D. Kocher . 2009. “Sexual Conflict Resolved by Invasion of a Novel Sex Determiner in Lake Malawi Cichlid Fishes.” Science 326, no. 5955: 998–1001. 10.1126/science.1174705. 19797625 PMC3174268

[mec17821-bib-0088] Santos, M. E. , J. F. Lopes , and C. F. Kratochwil . 2023. “East African Cichlid Fishes.” EvoDevo 14, no. 1: 1. 10.1186/s13227-022-00205-5. 36604760 PMC9814215

[mec17821-bib-0089] Schedel, F. D. B. , Z. Musilova , and U. K. Schliewen . 2019. “East African Cichlid Lineages (Teleostei: Cichlidae) Might Be Older Than Their Ancient Host Lakes: New Divergence Estimates for the East African Cichlid Radiation.” BMC Evolutionary Biology 19, no. 1: 94. 10.1186/s12862-019-1417-0. 31023223 PMC6482553

[mec17821-bib-0090] Schultheis, C. , A. Böhne , M. Schartl , J. N. Volff , and D. Galiana‐Arnoux . 2009. “Sex Determination Diversity and Sex Chromosome Evolution in Poeciliid Fish.” Sexual Development 3, no. 2–3: 68–77. 10.1159/000223072. 19684452

[mec17821-bib-0091] Schwander, T. , R. Libbrecht , and L. Keller . 2014. “Supergenes and Complex Phenotypes.” Current Biology 24, no. 7: R288–R294. 10.1016/j.cub.2014.01.056. 24698381

[mec17821-bib-0092] Ser, J. R. , R. B. Roberts , and T. D. Kocher . 2010. “Multiple Interacting Loci Control Sex Determination in Lake Malawi Cichlid Fish.” Evolution 64, no. 2: 486–501. 10.1111/j.1558-5646.2009.00871.x. 19863587 PMC3176681

[mec17821-bib-0093] Serrato‐Capuchina, A. , and D. R. Matute . 2018. “The Role of Transposable Elements in Speciation.” Genes 9, no. 5: 254. 10.3390/genes9050254. 29762547 PMC5977194

[mec17821-bib-0094] Seutin, G. , B. N. White , and P. T. Boag . 1991. “Preservation of Avian Blood and Tissue Samples for DNA Analyses.” Canadian Journal of Zoology 69, no. 1: 82–90. 10.1139/z91-013.

[mec17821-bib-0095] Sharakhov, I. V. , and M. V. Sharakhova . 2024. “Chromosomal Inversions and Their Impact on Insect Evolution.” Current Opinion in Insect Science 66: 101280. 10.1016/j.cois.2024.101280. 39374869 PMC11611660

[mec17821-bib-0096] Siggers, P. , L. Smith , and A. Greenfield . 2002. “Sexually Dimorphic Expression of Gata‐2 During Mouse Gonad Development.” Mechanisms of Development 111, no. 1–2: 159–162. 10.1016/s0925-4773(01)00602-5. 11804789

[mec17821-bib-0097] Smit, A. , R. Hubley , and P. Green . 2013. “RepeatMasker Open‐4.0 [Computer Software].” www.repeatmasker.org.

[mec17821-bib-0098] Streelman, J. T. , R. C. Albertson , and T. D. Kocher . 2003. “Genome Mapping of the Orange Blotch Colour Pattern in Cichlid Fishes.” Molecular Ecology 12, no. 9: 2465–2471. 10.1046/j.1365-294X.2003.01920.x. 12919484

[mec17821-bib-0099] The Galaxy Platform for Accessible, Reproducible, and Collaborative Data Analyses . 2024. 10.1093/nar/gkae410. PMC11223835 38769056

[mec17821-bib-0100] Thompson, M. J. , and C. D. Jiggins . 2014. “Supergenes and Their Role in Evolution.” Heredity (Edinb) 113, no. 1: 1–8. 10.1038/hdy.2014.20. 24642887 PMC4815649

[mec17821-bib-0101] Trapnell, C. , A. Roberts , L. Goff , et al. 2012. “Differential Gene and Transcript Expression Analysis of RNA‐Seq Experiments With TopHat and Cufflinks.” Nature Protocols 7, no. 3: 562–578. 10.1038/nprot.2012.016. 22383036 PMC3334321

[mec17821-bib-0102] Van der Auwera, G. A. , and B. D. O'Connor . 2020. Genomics in the Cloud: Using Docker, GATK, and WDL in Terra (1st Edition). O'Reilly Media.

[mec17821-bib-0103] Van Doorn, G. S. , and M. Kirkpatrick . 2007. “Turnover of Sex Chromosomes Induced by Sexual Conflict.” Nature 449, no. 7164: 909–912. 10.1038/nature06178. 17943130

[mec17821-bib-0104] Van Doorn, G. S. , and M. Kirkpatrick . 2010. “Transitions Between Male and Female Heterogamety Caused by Sex‐Antagonistic Selection.” Genetics 186, no. 2: 629–645. 10.1534/genetics.110.118596. 20628036 PMC2954476

[mec17821-bib-0105] Van Valen, L. , and R. Levins . 1968. “The Origins of Inversion Polymorphisms.” American Naturalist 102, no. 923: 5–24. 10.1086/282520.

[mec17821-bib-0106] VanderWielen, B. D. , Z. Yuan , D. R. Friedmann , and R. A. Kovall . 2011. “Transcriptional Repression in the Notch Pathway: Thermodynamic Characterization of CSL‐MINT (Msx2‐Interacting Nuclear Target Protein) Complexes.” Journal of Biological Chemistry 286, no. 17: 14892–14902. 10.1074/jbc.M110.181156. 21372128 PMC3083192

[mec17821-bib-0107] Vicente, C. , A. Conchillo , M. A. García‐Sánchez , and M. D. Odero . 2012. “The Role of the GATA2 Transcription Factor in Normal and Malignant Hematopoiesis.” Critical Reviews in Oncology/Hematology 82, no. 1: 1–17. 10.1016/j.critrevonc.2011.04.007. 21605981

[mec17821-bib-0108] Wang, C. , B. Lu , T. Li , et al. 2021. “Nile Tilapia: A Model for Studying Teleost Color Patterns.” Journal of Heredity 112, no. 5: 469–484. 10.1093/jhered/esab018. 34027978

[mec17821-bib-0109] Wang, J. , J.‐K. Na , Q. Yu , et al. 2012. “Sequencing Papaya X and Yh Chromosomes Reveals Molecular Basis of Incipient Sex Chromosome Evolution.” Proceedings of the National Academy of Sciences of the United States of America 109, no. 34: 13710–13715. 10.1073/pnas.1207833109. 22869747 PMC3427123

[mec17821-bib-0110] Wang, J. , W. Tao , T. D. Kocher , and D. Wang . 2024. “Sex Chromosome Turnover and Biodiversity in Fishes.” Journal of Genetics and Genomics 51, no. 12: 1351–1360. 10.1016/j.jgg.2024.08.008. 39233051

[mec17821-bib-0111] Wang, J. , Y. Wurm , M. Nipitwattanaphon , et al. 2013. “A Y‐Like Social Chromosome Causes Alternative Colony Organization in Fire Ants.” Nature 493, no. 7434: 664–668. 10.1038/nature11832. 23334415

[mec17821-bib-0112] Wang, Y. 2022. “A Comparative Study of HiCanu and Hifiasm.” In ICoMS'22: Proceedings of the 2022 5th International Conference on Mathematics and Statistics, 100–104. Association for Computing Machinery. 10.1145/3545839.3545855.

[mec17821-bib-0113] Wells, J. N. , and C. Feschotte . 2020. “A Field Guide to Eukaryotic Transposable Elements.” Annual Review of Genetics 54: 539–561. 10.1146/annurev-genet-040620-022145. PMC8293684 32955944

[mec17821-bib-0114] Werren, J. H. , and L. W. Beukeboom . 1998. “Sex Determination, Sex Ratios, and Genetic Conflict.” Annual Review of Ecology and Systematics 29, no. 1: 233–261. 10.1146/annurev.ecolsys.29.1.233.

[mec17821-bib-0115] Whiteley, S. L. , C. E. Holleley , and A. Georges . 2022. “Developmental Dynamics of Sex Reprogramming by High Incubation Temperatures in a Dragon Lizard.” BMC Genomics 23, no. 1: 322. 10.1186/s12864-022-08544-2. 35459109 PMC9034607

[mec17821-bib-0116] Wick, R. R. , M. B. Schultz , J. Zobel , and K. E. Holt . 2015. “Bandage: Interactive Visualization of de Novo Genome Assemblies.” Bioinformatics 31, no. 20: 3350–3352. 10.1093/bioinformatics/btv383. 26099265 PMC4595904

[mec17821-bib-0117] Yang, H. , J. Ma , Z. Wan , et al. 2021. “Characterization of Sheep Spermatogenesis Through Single‐Cell RNA Sequencing.” FASEB Journal 35, no. 2: e21187. 10.1096/fj.202001035RRR. 33197070

[mec17821-bib-0118] Yi, X. , D. Wang , K. Reid , X. Feng , A. Löytynoja , and J. Merilä . 2024. “Sex Chromosome Turnover in Hybridizing Stickleback Lineages.” Evolution Letters 8, no. 5: 658–668. 10.1093/evlett/qrae019. 39328282 PMC11424075

[mec17821-bib-0119] Yoshida, K. , T. Makino , K. Yamaguchi , et al. 2014. “Sex Chromosome Turnover Contributes to Genomic Divergence Between Incipient Stickleback Species.” PLoS Genetics 10, no. 3: e1004223. 10.1371/journal.pgen.1004223. 24625862 PMC3953013

[mec17821-bib-0120] Zhou, Q. , J. Zhang , D. Bachtrog , et al. 2014. “Complex Evolutionary Trajectories of Sex Chromosomes Across Bird Taxa.” Science 346, no. 6215: 1246338. 10.1126/science.1246338. 25504727 PMC6445272

[mec17821-bib-0121] Zhou, Y. , Z. Yu , D. Chebotarov , et al. 2023. “Pan‐Genome Inversion Index Reveals Evolutionary Insights Into the Subpopulation Structure of Asian Rice.” Nature Communications 14, no. 1: 1567. 10.1038/s41467-023-37004-y. PMC10030860 36944612

