# Supplementary material for: A Chromosome Inversion Creates a Supergene for Sex and Colour in Lake Malawi Cichlids
Source: Mol Ecol. 2025 Jun 10;34(20):e17821. doi: 10.1111/mec.17821 (PMC12530302; doi:10.1111/mec.17821)
Supplement: Supplementary file 1 — Figure S1. [file MEC-34-e17821-s007.docx]

**Supplemental Figure 1.** Images of the two specimens used for genome sequencing. a) *M. zebra* orange-blotched (OB morph) male, b) *L. trewavasae* orange (O morph) female.

**
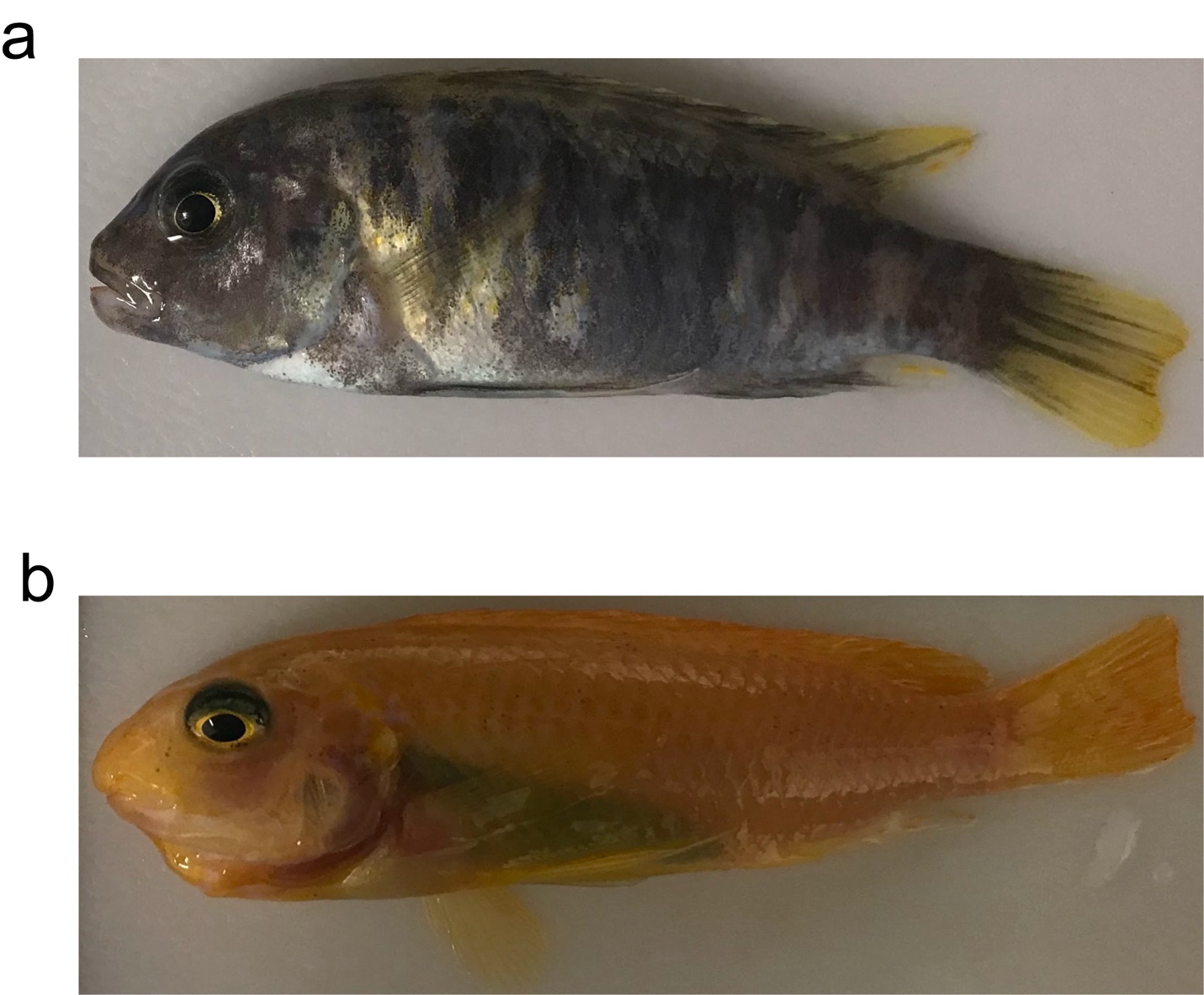
**
