# Supplementary material for: A Chromosome Inversion Creates a Supergene for Sex and Colour in Lake Malawi Cichlids
Source: Mol Ecol. 2025 Jun 10;34(20):e17821. doi: 10.1111/mec.17821 (PMC12530302; doi:10.1111/mec.17821)
Supplement: Supplementary file 2 — Figure S2. [file MEC-34-e17821-s001.docx]

**Supplemental Figure 2**. Pairwise dot plots of the genomes of interest.

a) Whole genome comparison of *M. zebra* UMD2a against *L. trewavasae* LatrZW. LatrZW features a possible B chromosome that aligns against the unanchored scaffolds from UMD2a, seen at the topmost part of the plot.

b) *M. zebra* UMD2a against *L. trewavasae* LatrZW zoom on LG5 shows one large inversion at the middle of the chromosome and several smaller inversions.

c) Whole genome comparison of *M. zebra* UMD2a against *M. zebra* MezeOBm. MezeOBm features a possible B chromosome that aligns against the unanchored scaffolds from UMD2a, seen at the topmost part of the plot.

d) *M. zebra* UMD2a against *L. trewavasae* LatrZW zoom on LG5 shows one large inversion at the middle of the chromosome and several smaller inversions. MezeOBm’s LG5 is notably shorter than that of UMD2a due to assembly challenges.

e) Whole genome comparison of *M. zebra* MezeOBm against *L. trewavasae* LatrZW. These two genomes feature B chromosomes that are somewhat similar to each other (see top right of plot).

f) *M. zebra* MezeOBm against *L. trewavasae* LatrZW zoom on LG5 shows that the two inversion haplotypes are very similar other than the missing portion of MezeOBm’s LG5, which does not correspond to the inversion.

g) Comparison of the two haplotigs of LG5 in *L. trewavasae* LatrZW. In this assembly, the non-inverted haplotig is missing 11.8Mb of sequence encompassing the inverted region.

h) Comparison of the two haplotigs of LG5 in *M. zebra* MezeOBm, where the inverted haplotig is missing sequence outside the inversion.

a) Whole genome comparison of *M. zebra* UMD2a against *L. trewavasae* LatrZW. LatrZW features a possible B chromosome that aligns against the unanchored scaffolds from UMD2a, seen at the topmost part of the plot.

**a**


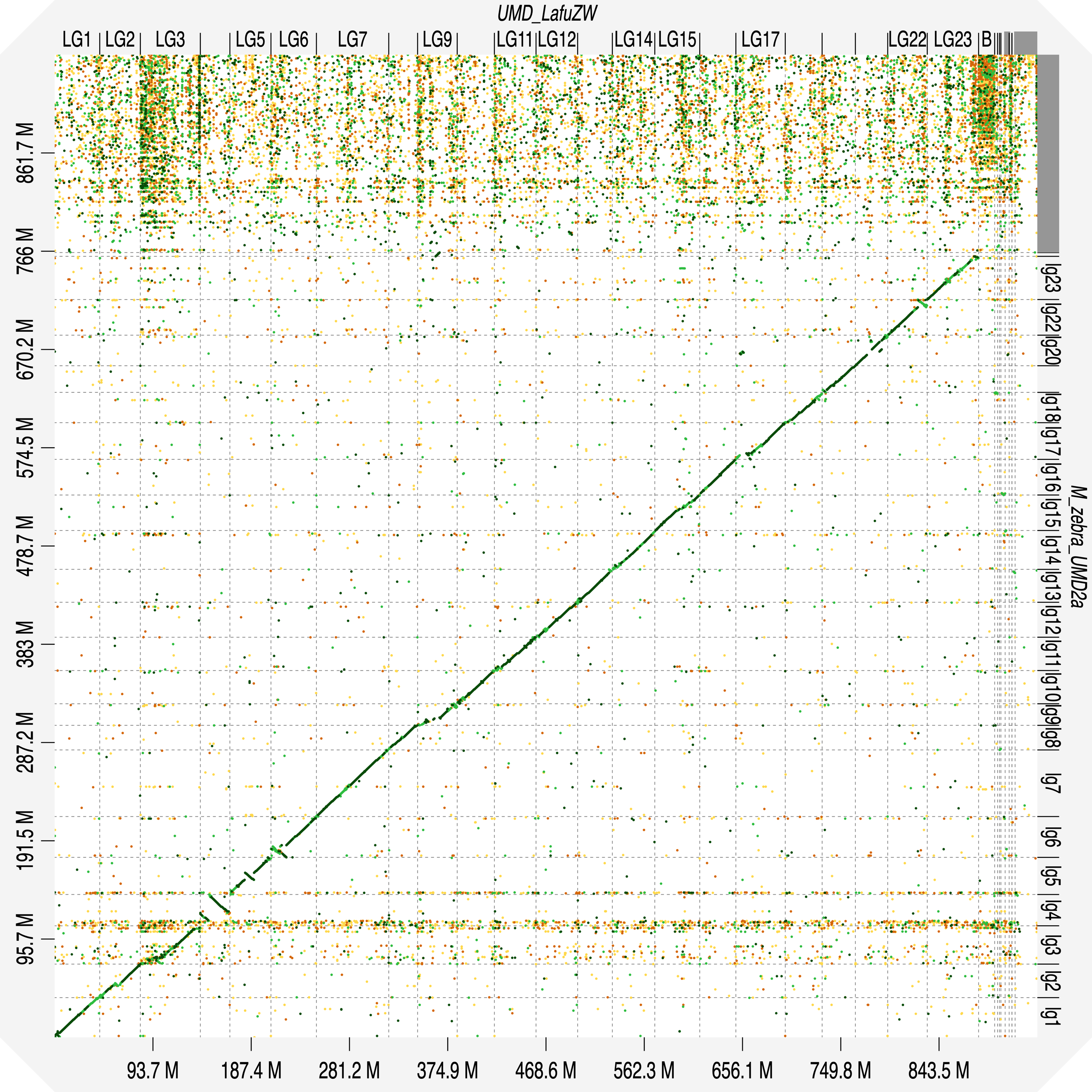


*UMD_LatrZW*

b) *M. zebra* UMD2a against *L. trewavasae* LatrZW zoom on LG5 shows one large inversion at the middle of the chromosome and several smaller inversions.


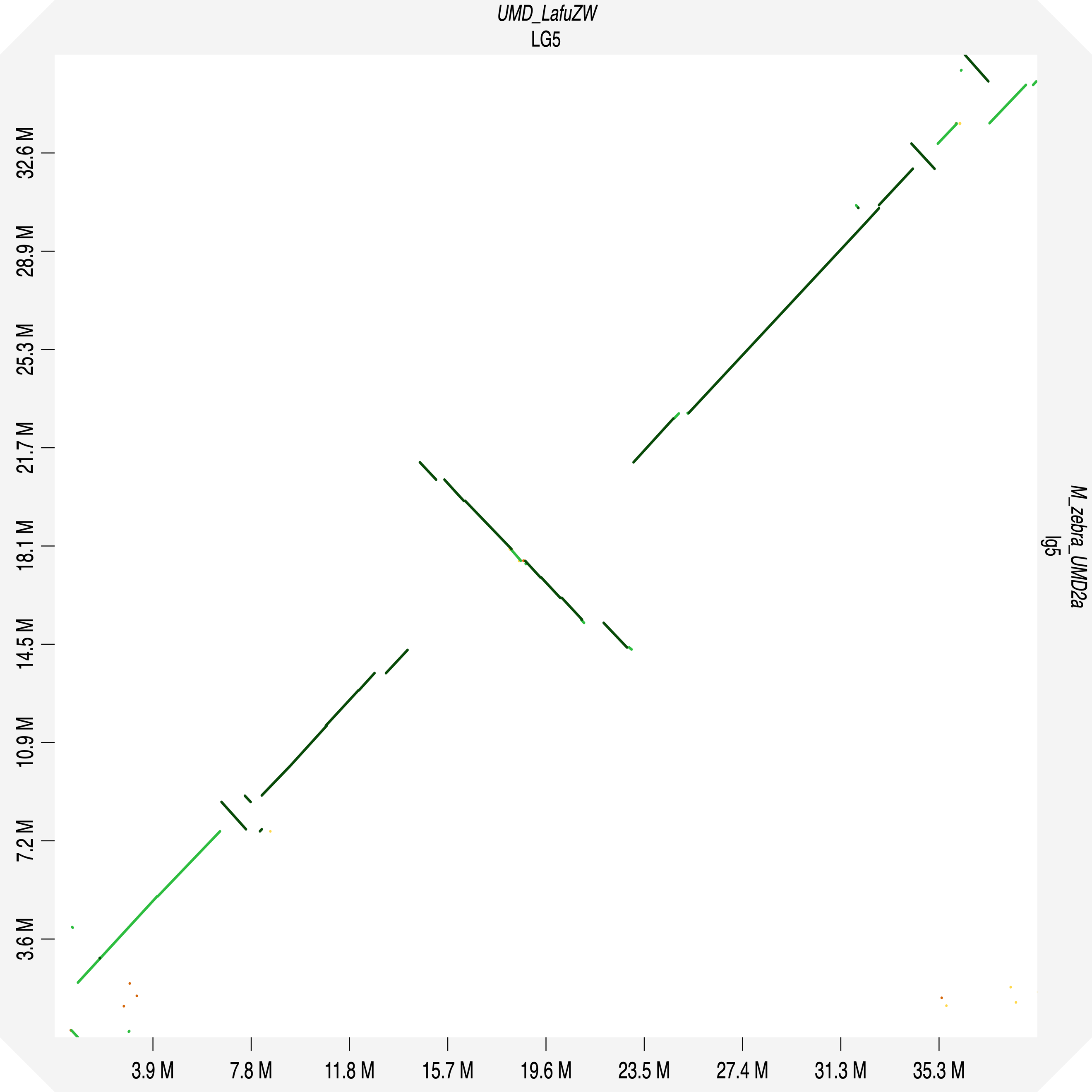


**b**

*UMD_LatrZW*

c) Whole genome comparison of *M. zebra* UMD2a against *M. zebra* MezeOBm. MezeOBm features a possible B chromosome that aligns against the unanchored scaffolds from UMD2a, seen at the topmost part of the plot.


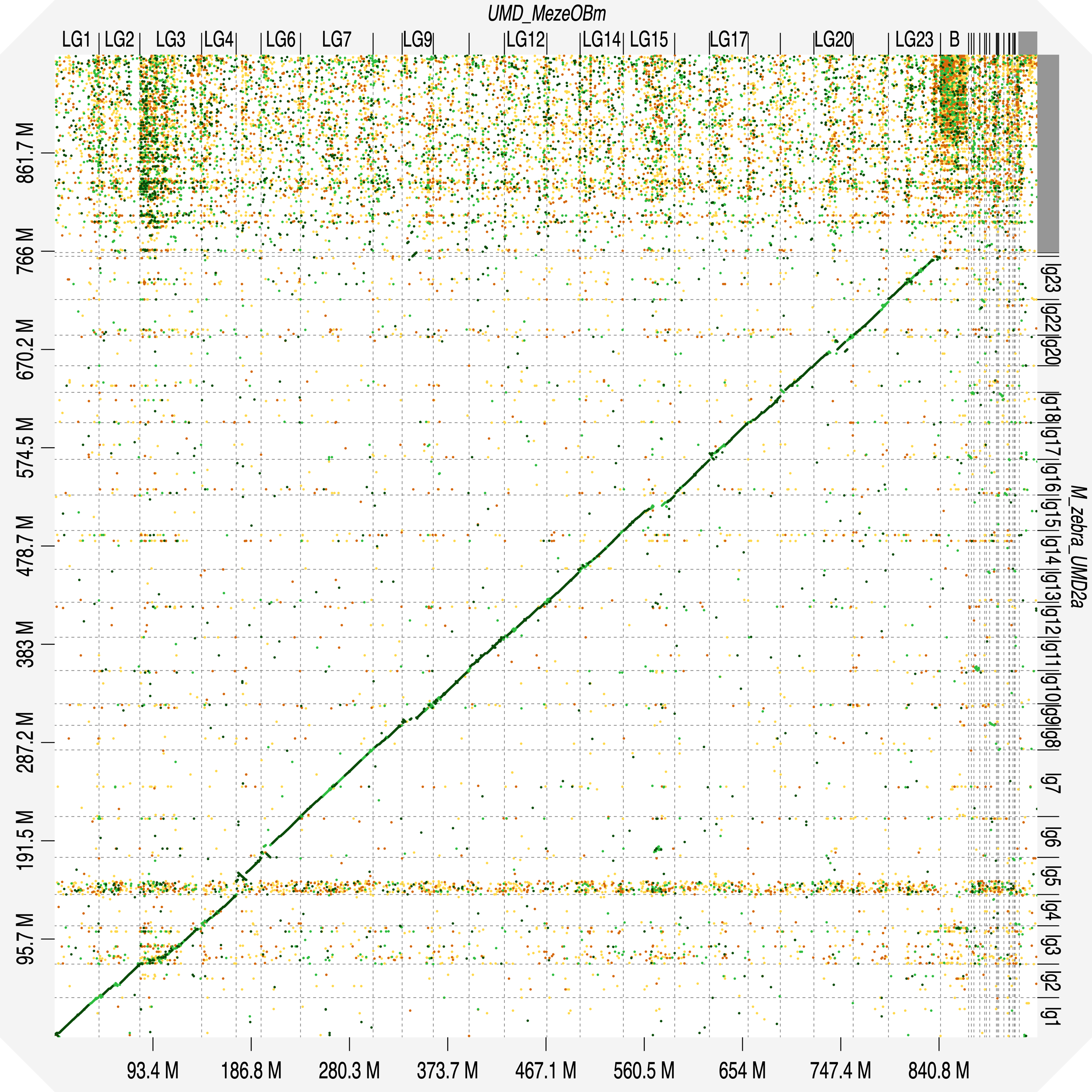


**c**

d) *M. zebra* UMD2a against *L. trewavasae* LatrZW zoom on LG5 shows one large inversion at the middle of the chromosome and several smaller inversions. MezeOBm’s LG5 is notably shorter than that of UMD2a due to assembly challenges.


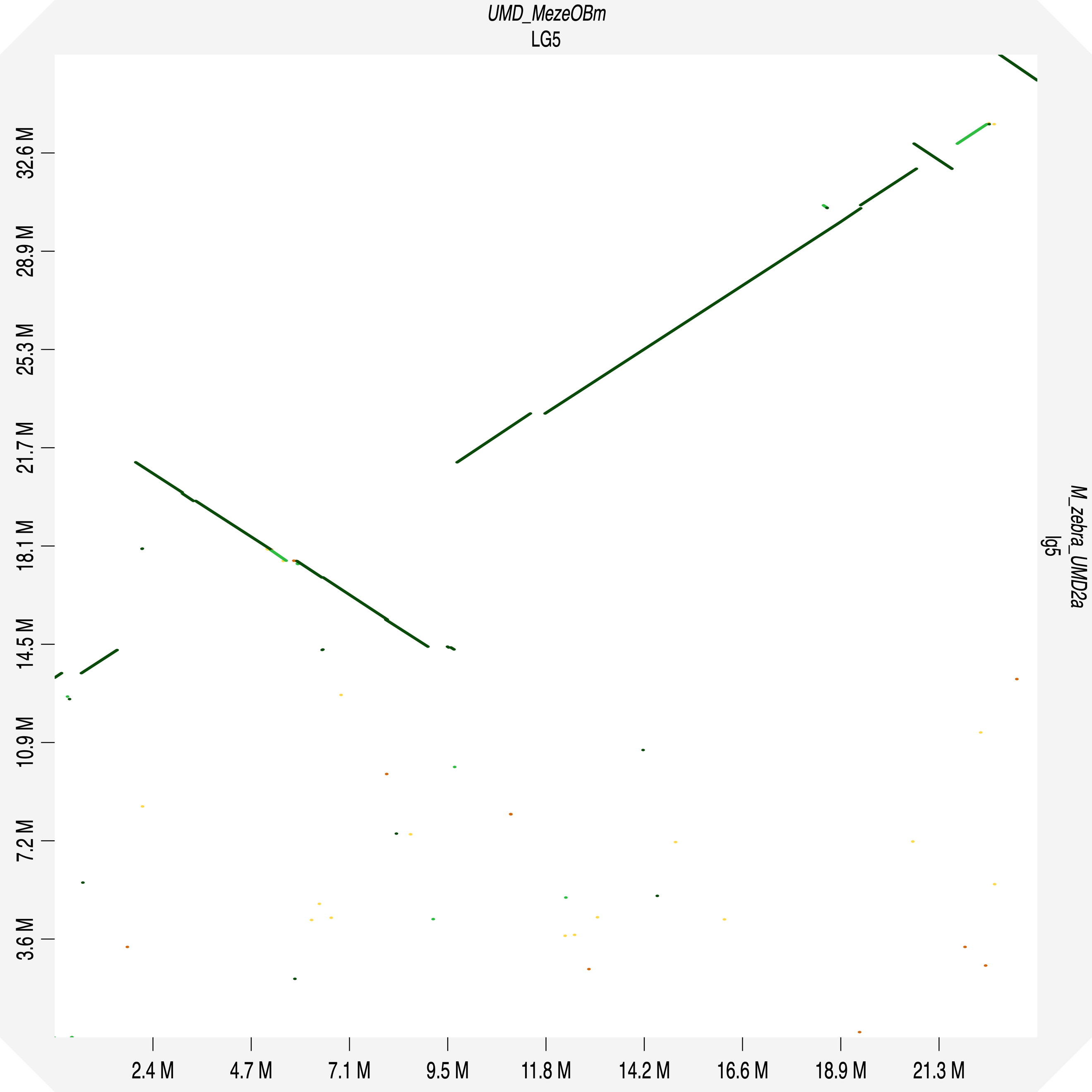


**d**

e) Whole genome comparison of *M. zebra* MezeOBm against *L. trewavasae* LatrZW. These two genomes feature B chromosomes that are somewhat similar to each other (see top right of plot).


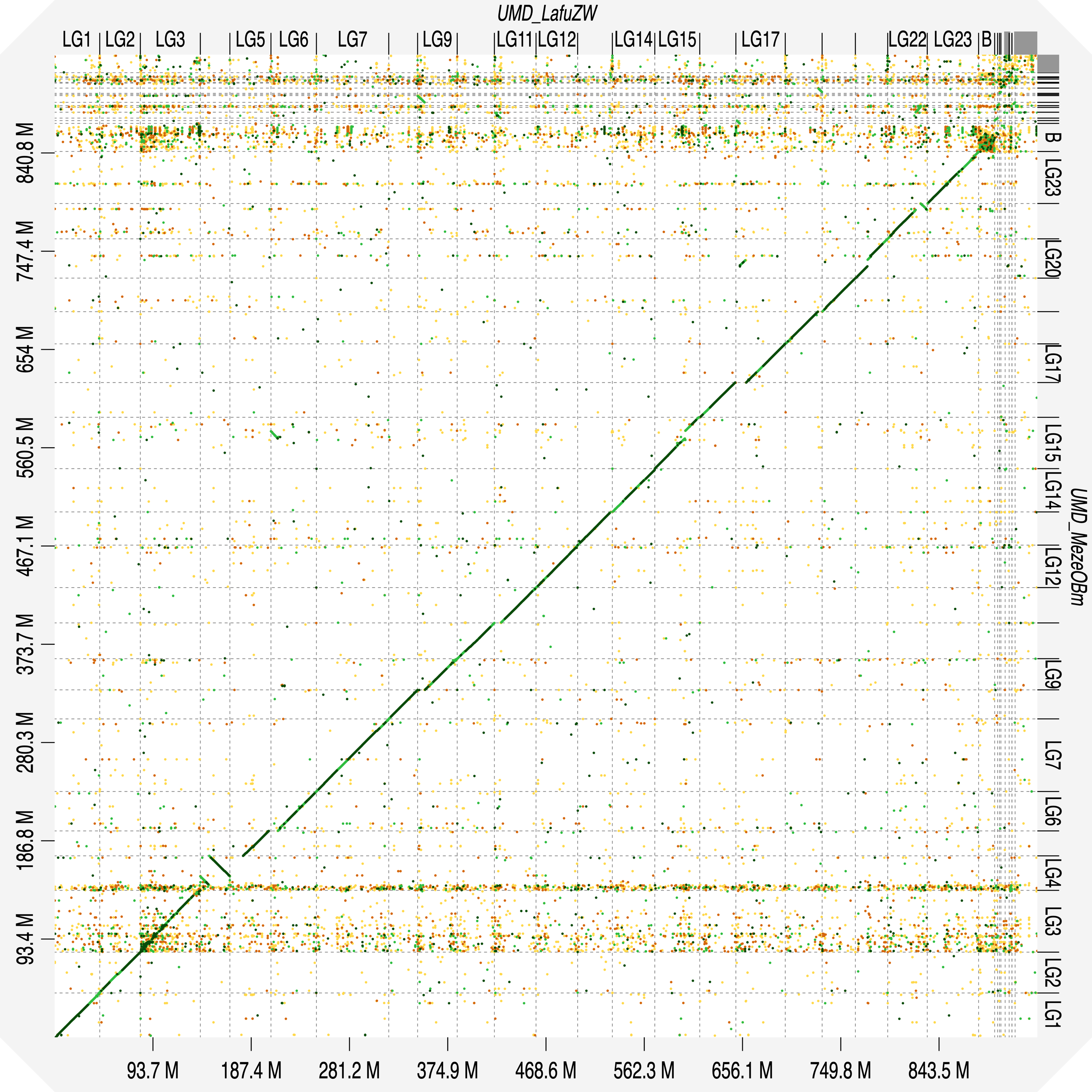


**e**

*UMD_LatrZW*

f) *M. zebra* MezeOBm against *L. trewavasae* LatrZW zoom on LG5 shows that the two inversion haplotypes are very similar other than the missing portion of MezeOBm’s LG5, which does not correspond to the inversion.


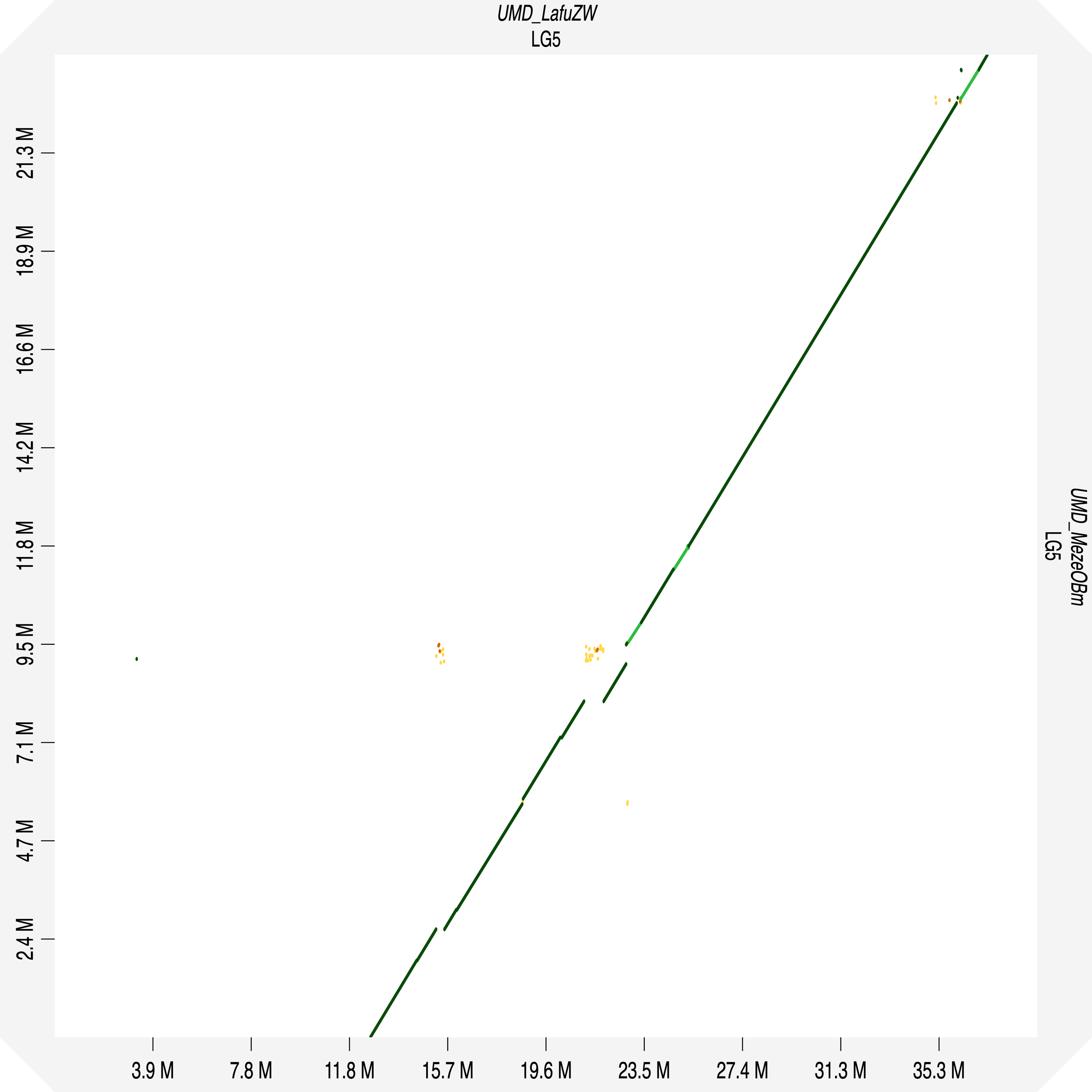


**f**

*UMD_LatrZW*

g) Comparison of the two haplotigs of LG5 in *L. trewavasae* LatrZW. In this assembly, the non-inverted haplotig is missing 11.8Mb of sequence encompassing the inverted region.


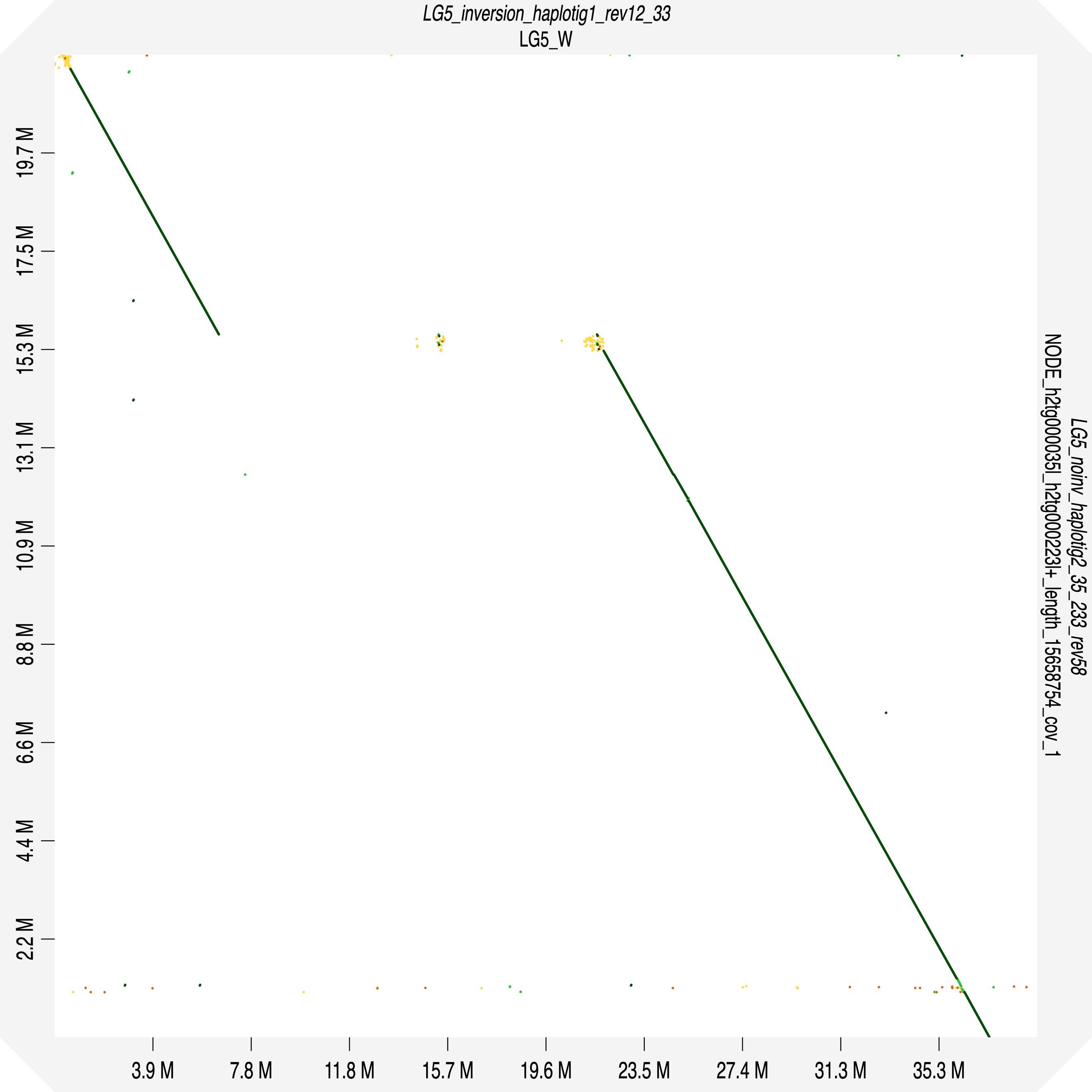


**g**

h) Comparison of the two haplotypes of LG5 in *M. zebra* MezeOBm, where the inverted haplotype is missing sequence outside the inversion.


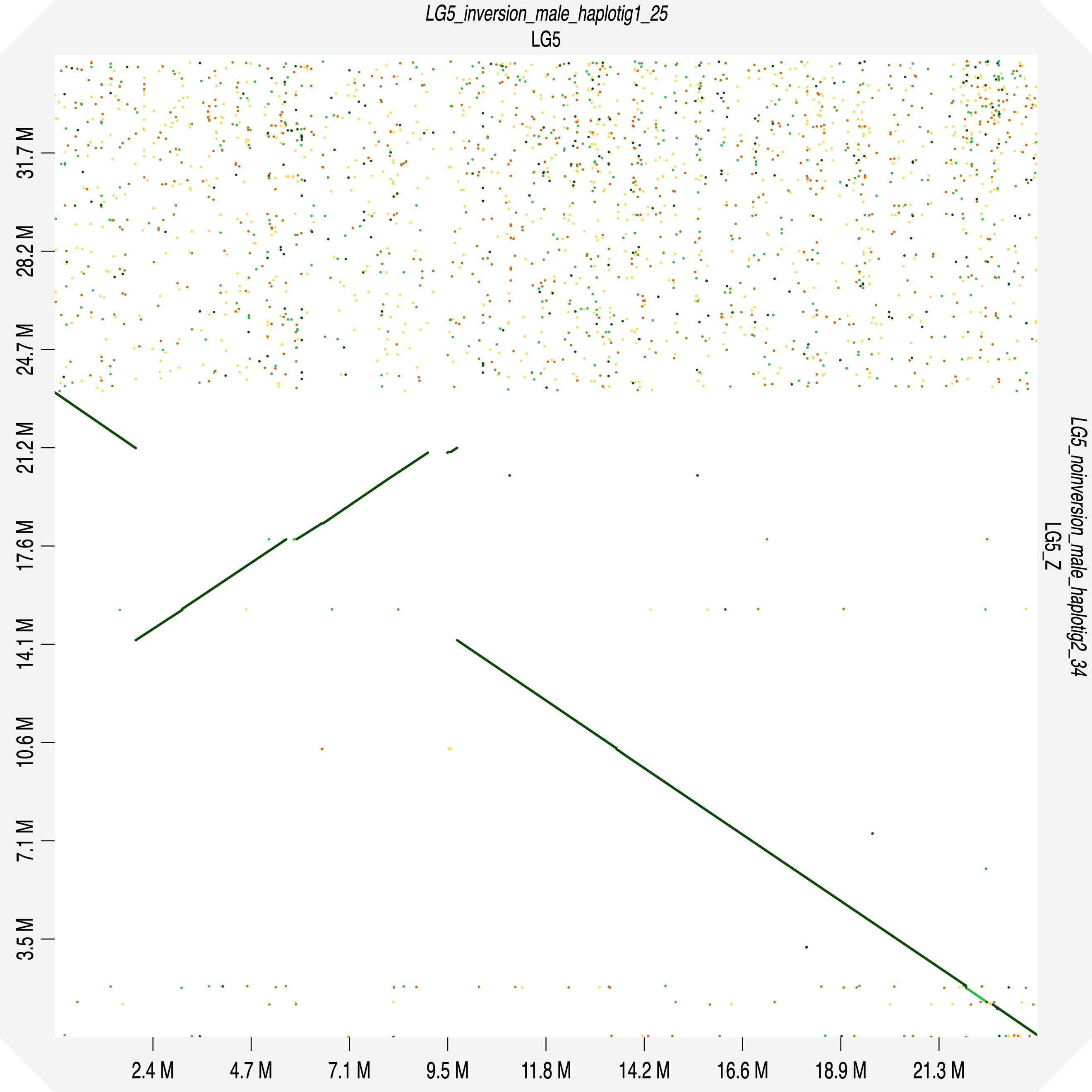


**h**
