# Supplementary material for: A Chromosome Inversion Creates a Supergene for Sex and Colour in Lake Malawi Cichlids
Source: Mol Ecol. 2025 Jun 10;34(20):e17821. doi: 10.1111/mec.17821 (PMC12530302; doi:10.1111/mec.17821)
Supplement: Supplementary file 3 — Figure S3. [file MEC-34-e17821-s011.docx]

**Supplemental Figure 3**. Fraction of transposable element bases in 200kb windows for contigs on LG5. a) *L. trewavasae*, b) *M. zebra*. Dashed lines indicate approximate inversion boundaries.

a

b
