# Supplementary material for: A Chromosome Inversion Creates a Supergene for Sex and Colour in Lake Malawi Cichlids
Source: Mol Ecol. 2025 Jun 10;34(20):e17821. doi: 10.1111/mec.17821 (PMC12530302; doi:10.1111/mec.17821)
Supplement: Supplementary file 4 — Figure S4. [file MEC-34-e17821-s006.docx]

**Supplemental Figure 4**. Transposable element content of the *L. trewavasae* inversion.
