# Supplementary material for: A Chromosome Inversion Creates a Supergene for Sex and Colour in Lake Malawi Cichlids
Source: Mol Ecol. 2025 Jun 10;34(20):e17821. doi: 10.1111/mec.17821 (PMC12530302; doi:10.1111/mec.17821)

**Supplemental Figure 4**. Whole genome plots for F_ST_ and sex patterned SNP allele frequency for populations segregating OB alleles aligned against *L. trewavasae* (LatrZW). Plots zooming in on LG5 are included for select populations.

Page 2. *Labeotropheus trewavasae*, Maison Reef, BB males vs. BB females for whole genome.

Page 3. *Labeotropheus trewavasae*, Maison Reef, BB males vs. BB females for linkage group 5.

Page 4. *Labeotropheus trewavasae*, Maison Reef, BB males vs. OB females for whole genome.

Page 5. *Labeotropheus trewavasae*, Maison Reef, BB males vs. OB females for linkage group 5.

Page 6. *Labeotropheus trewavasae*, Maison Reef, OB females vs. BB females for whole genome.

Page 7. *Metriaclima callainos*, Nkhata Bay, Blue males vs. Blue females for whole genome.

Page 8. *Metriaclima callainos*, Nkhata Bay, Blue males vs. Blue females for linkage group 5.

Page 9. *Metriaclima callainos*, Nkhata Bay, Blue males vs. White females for whole genome.

Page 10. *Metriaclima callainos*, Nkhata Bay, Blue males vs. White females for linkage group 5.

Page 11. *Metriaclima callainos*, Nkhata Bay, White females vs. Blue females for whole genome.

Page 12. *Metriaclima callainos*, Nkhata Bay, White females vs. Blue females for linkage group 5.

Page 13. *Metriaclima callainos*, Lupingu, White males vs. White females for whole genome.

Page 14. *Metriaclima callainos*, Luwino, White males vs. White females for whole genome.

Page 15. *Metriaclima* 'zebra gold', Nkhata Bay, BB males vs. OB females for whole genome.

Page 16. *Metriaclima zebra*, Nkhata Bay, BB males x BB females for whole genome.

Page 17. *Metriaclima zebra*, Nkhata Bay, BB males x Orange females for whole genome.

Page 18. *Metriaclima zebra*, Nkhata Bay, OB females x BB females for whole genome.

Page 19. *Metriaclima zebra*, Nkhata Bay, OB females x Orange females for whole genome.

Page 20. *Metriaclima zebra*, Nkhata Bay, OB females x Orange females for linkage group 5.

Page 21. *Labeotropheus fuelleborni*, Thumbi West, BB males x BB females for whole genome.

Page 22. *Labeotropheus trewavasae*, Thumbi West, BB males x BB females for whole genome.

Page 23. *Metriaclima callainos*, Luwino White males vs. Nkhata Bay Blue females for whole genome.

Page 24. *Metriaclima callainos*, Nkhata Bay Blue males vs. Luwino White females for whole genome.

Page 25. *Metriaclima callainos*, Lupingu White males vs. Nkhata Bay Blue females for whole genome.

Page 26. *Metriaclima callainos*, Nkhata Bay Blue males vs. Lupingu White females for whole genome.

Page 2. *Labeotropheus trewavasae*, Maison Reef, BB males vs. BB females for whole genome. No signal is observed because the inversion is not present in either sample.


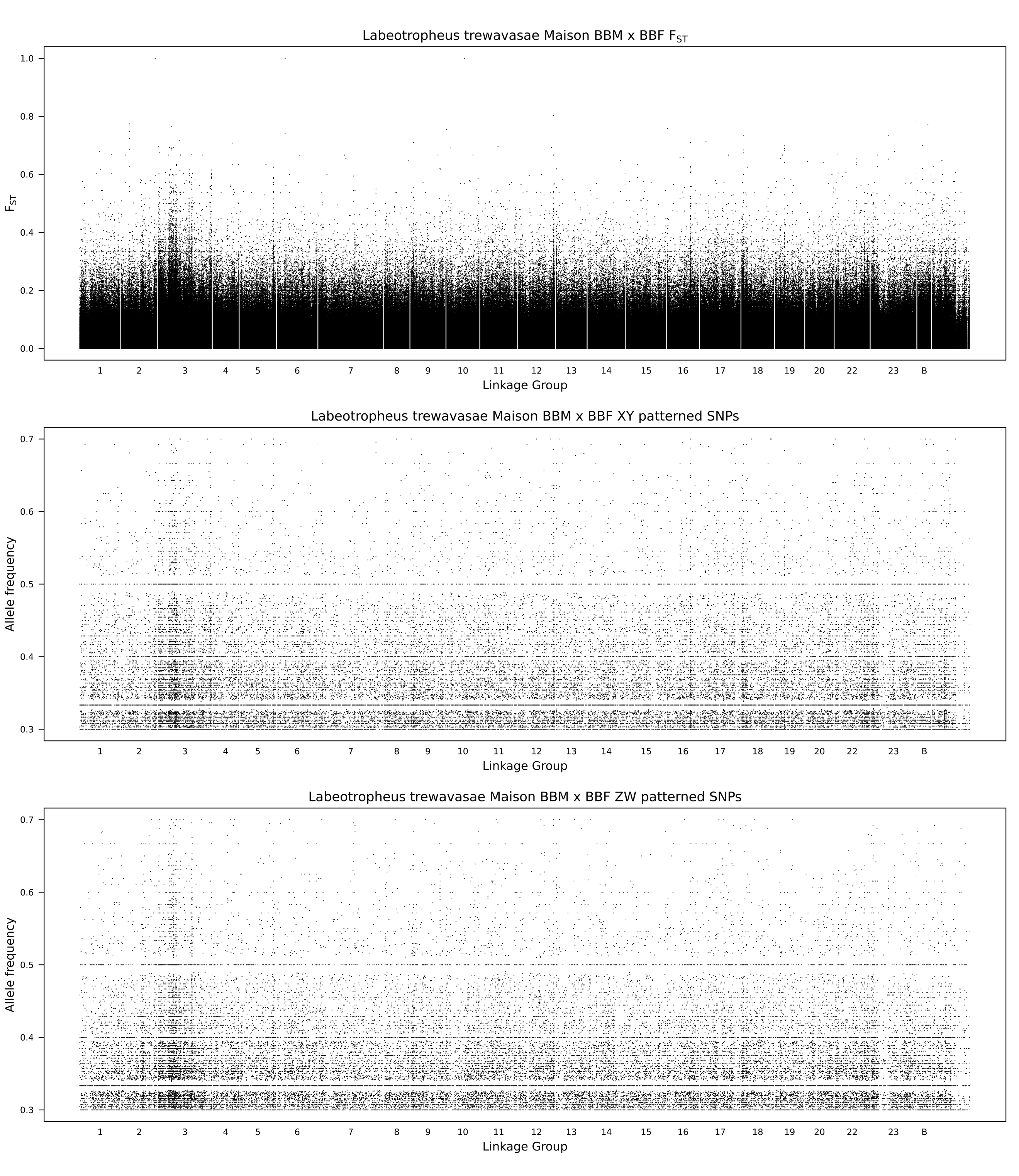


Page 3. *Labeotropheus trewavasae*, Maison Reef, BB males vs. BB females for linkage group 5. No signal is observed because the inversion is not present in either sample.


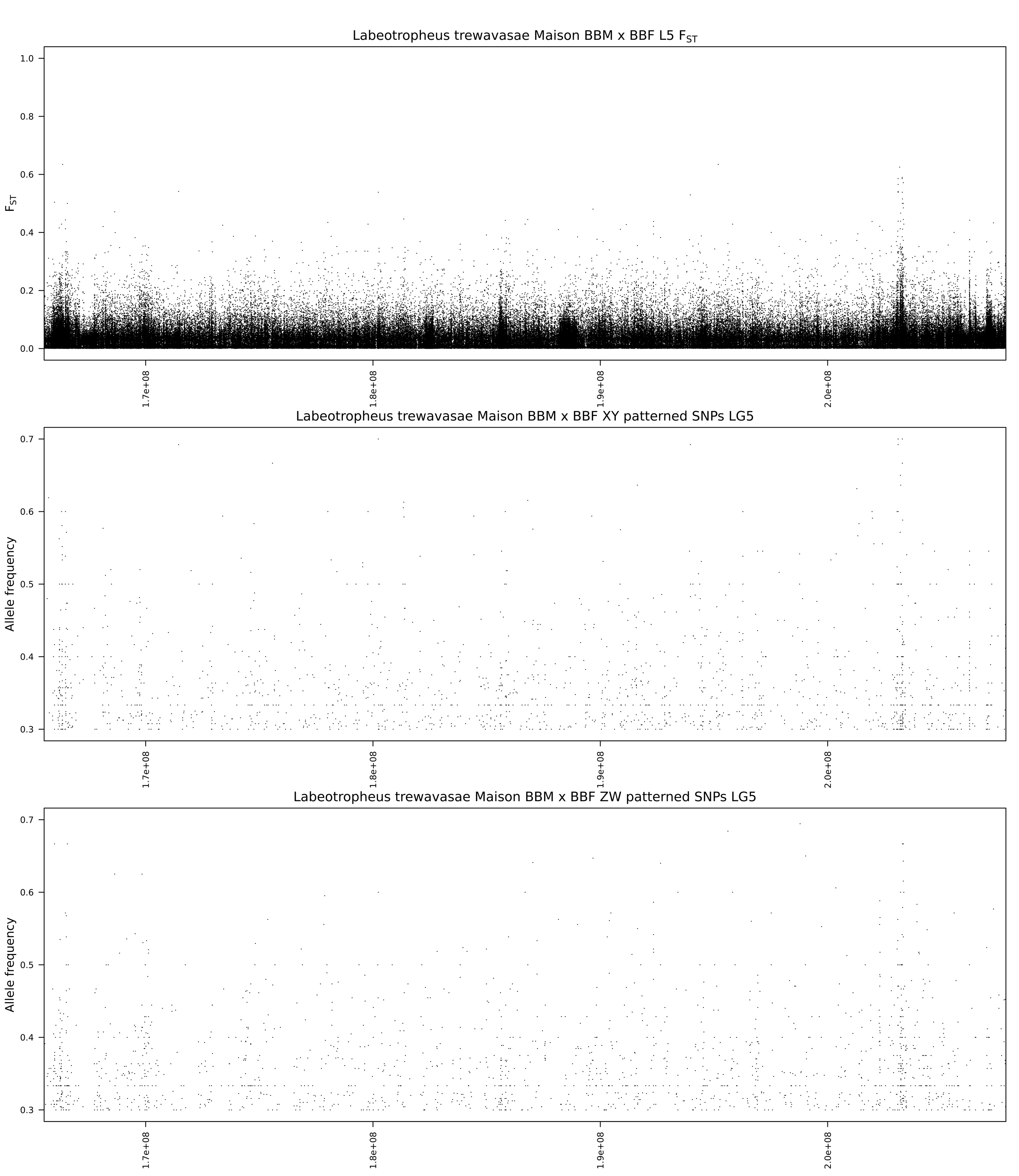


Page 4. *Labeotropheus trewavasae*, Maison Reef, BB males vs. OB females for whole genome. A strong signal is observed on linkage group 5 in the Fst and ZW-patterned SNPs because the OB females are heterozygous for the inversion.


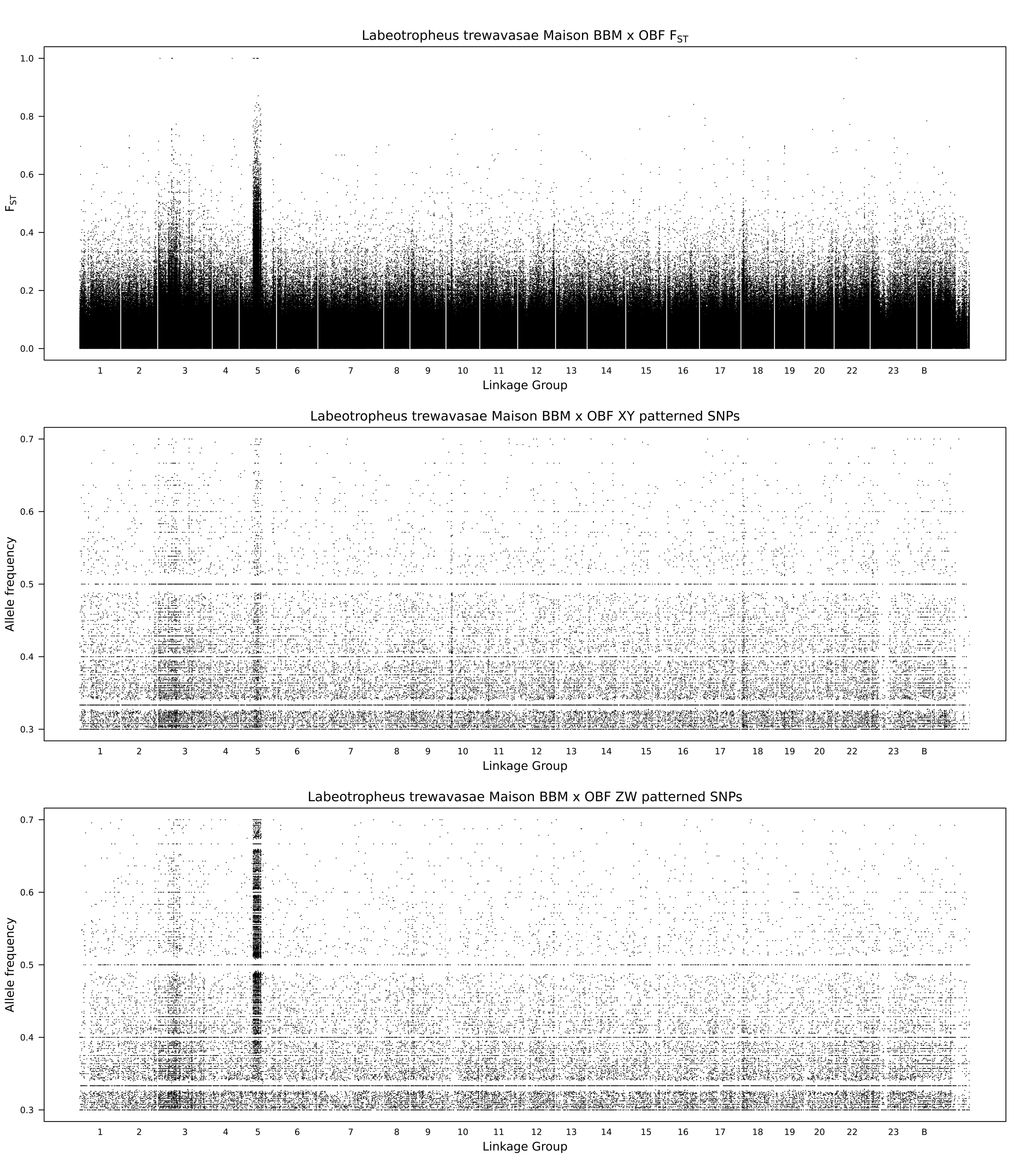


Page 5. *Labeotropheus trewavasae*, Maison Reef, BB males vs. OB females for linkage group 5. A strong signal is observed in the Fst and ZW-patterned SNPs because the OB females are heterozygous for the inversion.


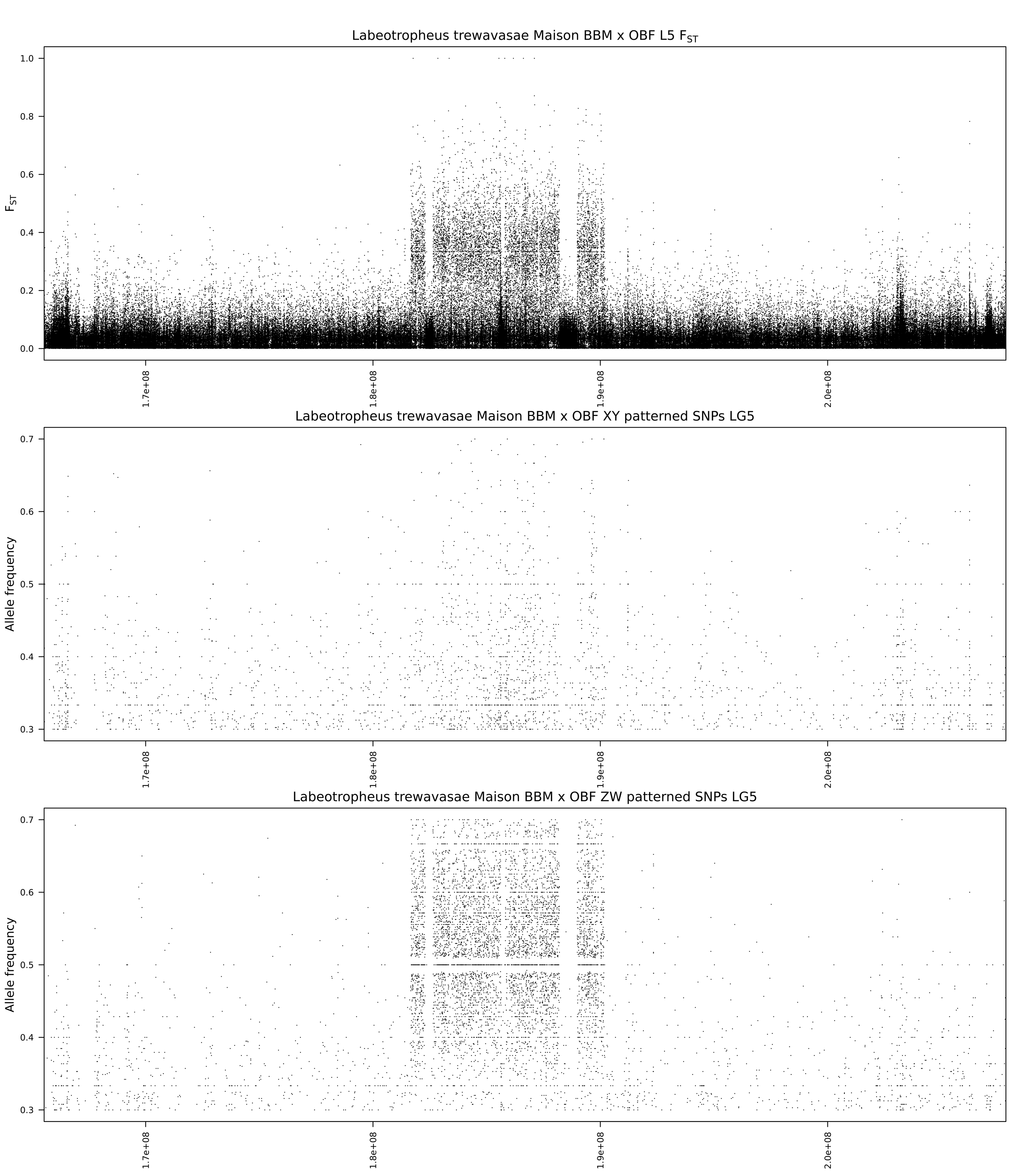


Page 6. *Labeotropheus trewavasae*, Maison Reef, OB females vs. BB females for whole genome. A strong signal is observed on linkage group 5 in the Fst and ZW-patterned SNPs because the OB females are heterozygous for the inversion, and the BB females are homozygous for the non-inverted haplotype.


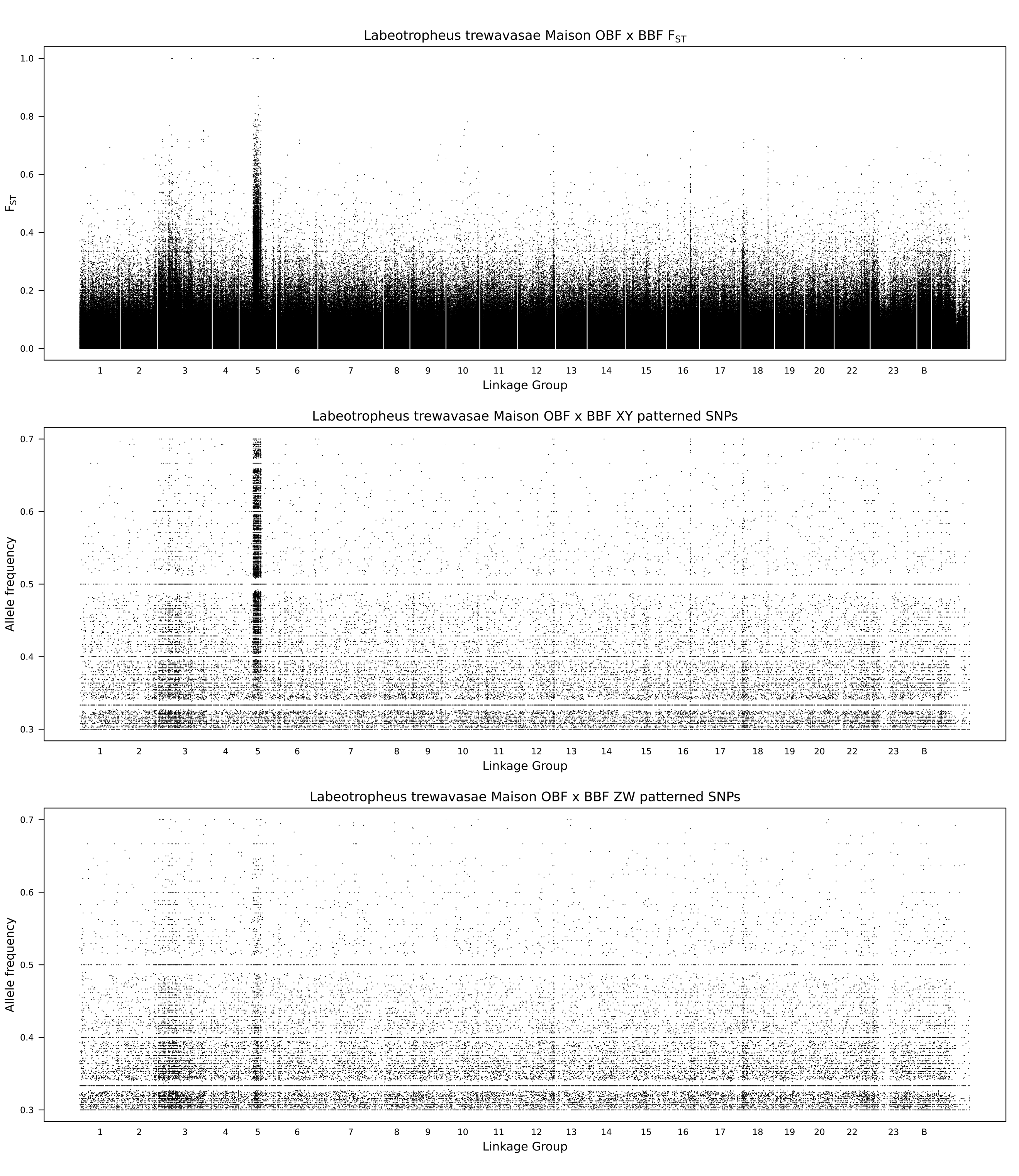


Page 7. *Metriaclima callainos*, Nkhata Bay, Blue males vs. Blue females for whole genome. No signal is observed because the Blue males and females are both homozygous for the non-inverted haplotype.


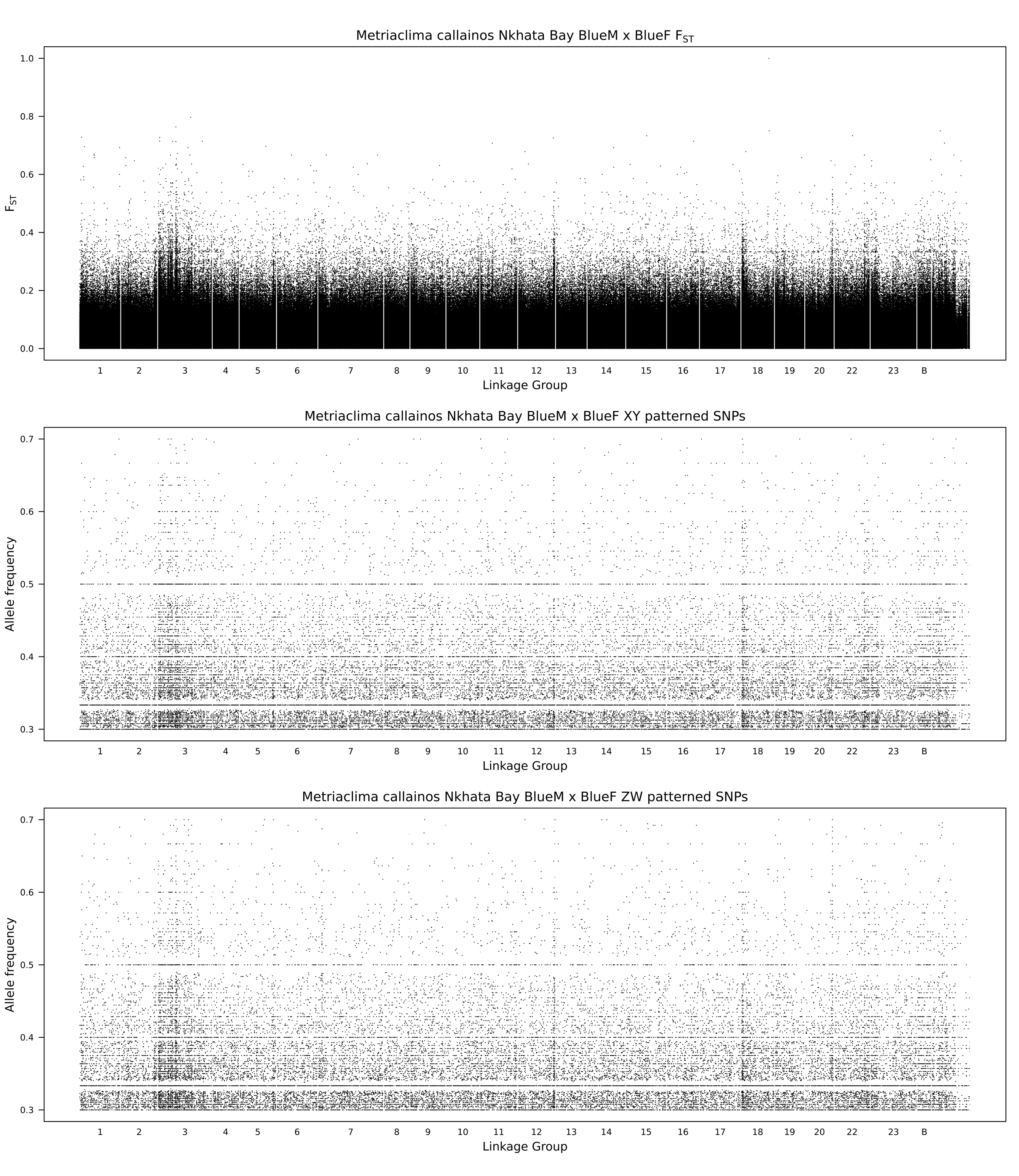


Page 8. *Metriaclima callainos*, Nkhata Bay, Blue males vs. Blue females for linkage group 5. No signal is observed because the Blue males and females are both homozygous for the non-inverted haplotype.


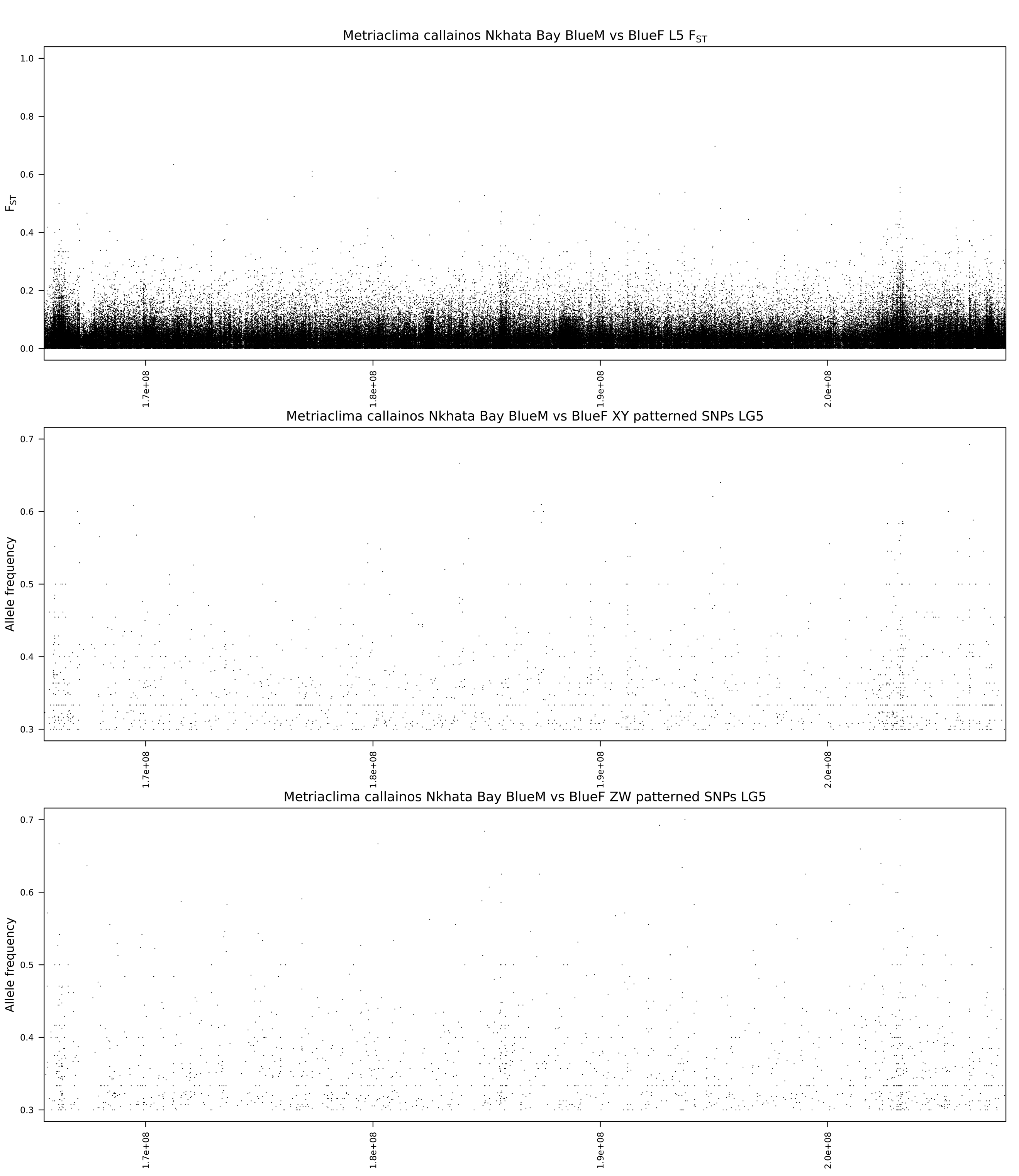


Page 9. *Metriaclima callainos*, Nkhata Bay, Blue males vs. White females for whole genome. A strong signal is observed on linkage group 5 in the Fst and ZW-patterned SNPs because the White females are heterozygous for the inversion.


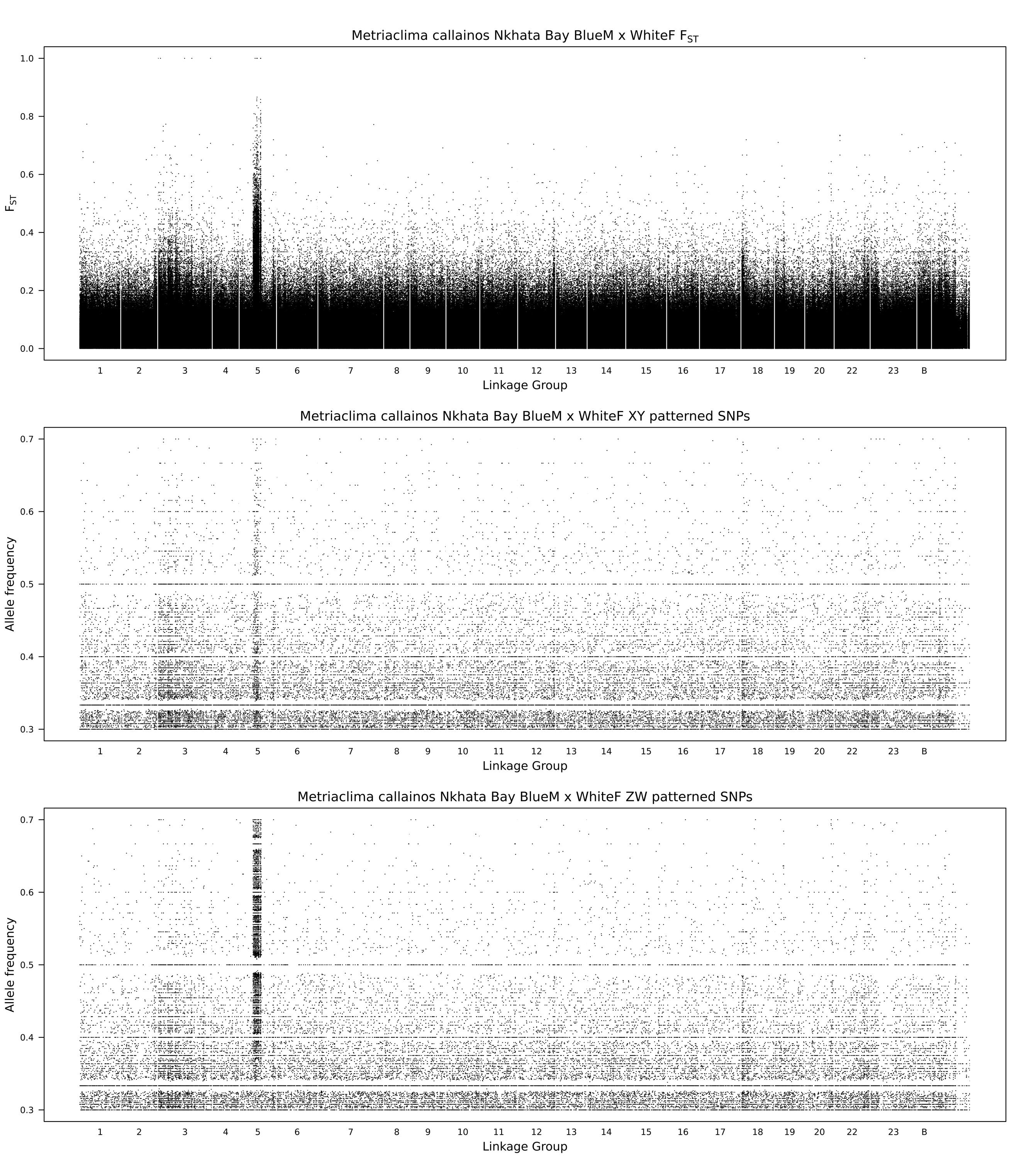


Page 10. *Metriaclima callainos*, Nkhata Bay, Blue males vs. White females for linkage group 5. A strong signal is observed in the Fst and ZW-patterned SNPs because the White females are heterozygous for the inversion.


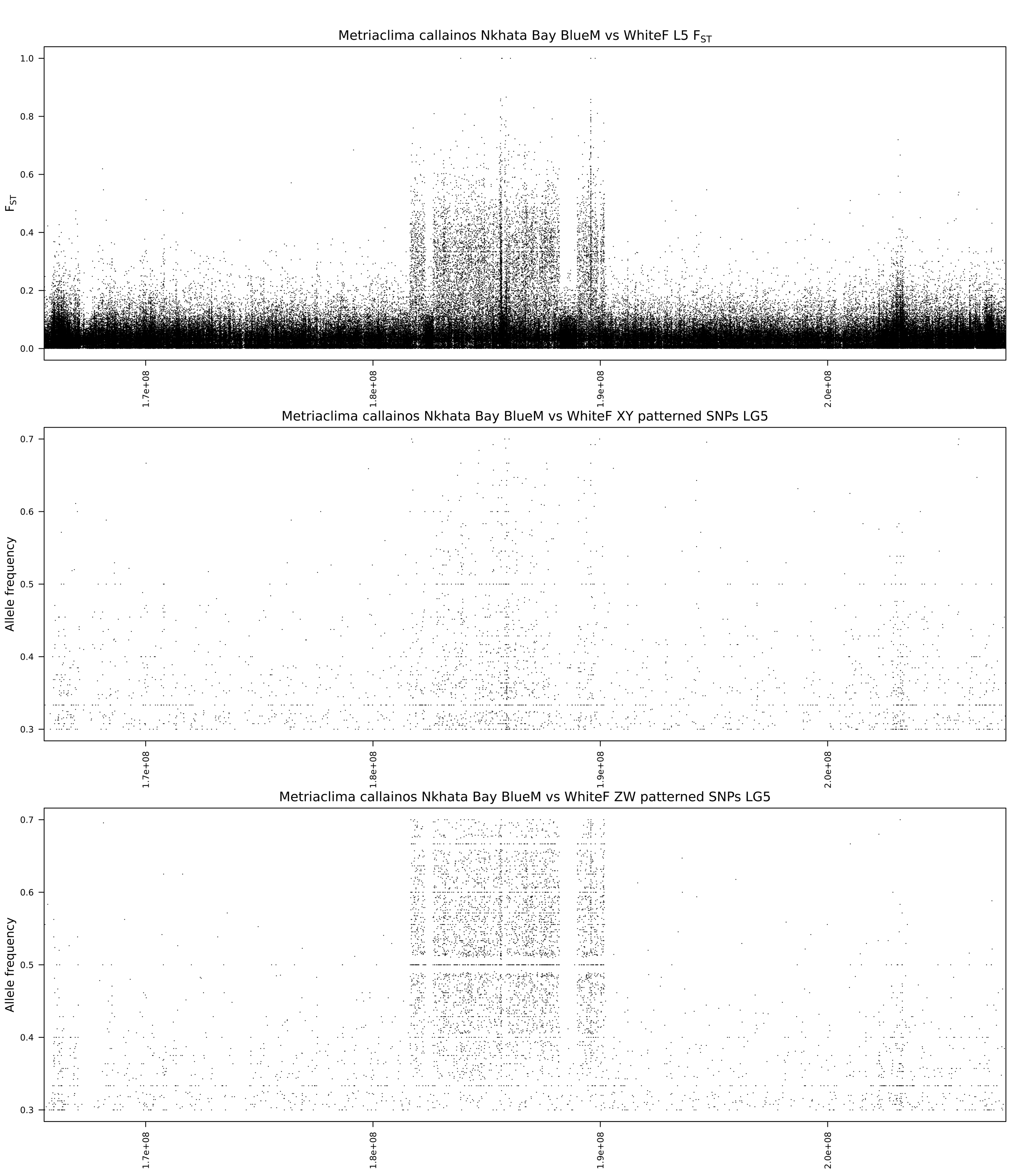


Page 11. *Metriaclima callainos*, Nkhata Bay, White females vs. Blue females for whole genome. A strong signal is observed on linkage group 5 in the Fst and ZW-patterned SNPs because the White females are heterozygous for the inversion.


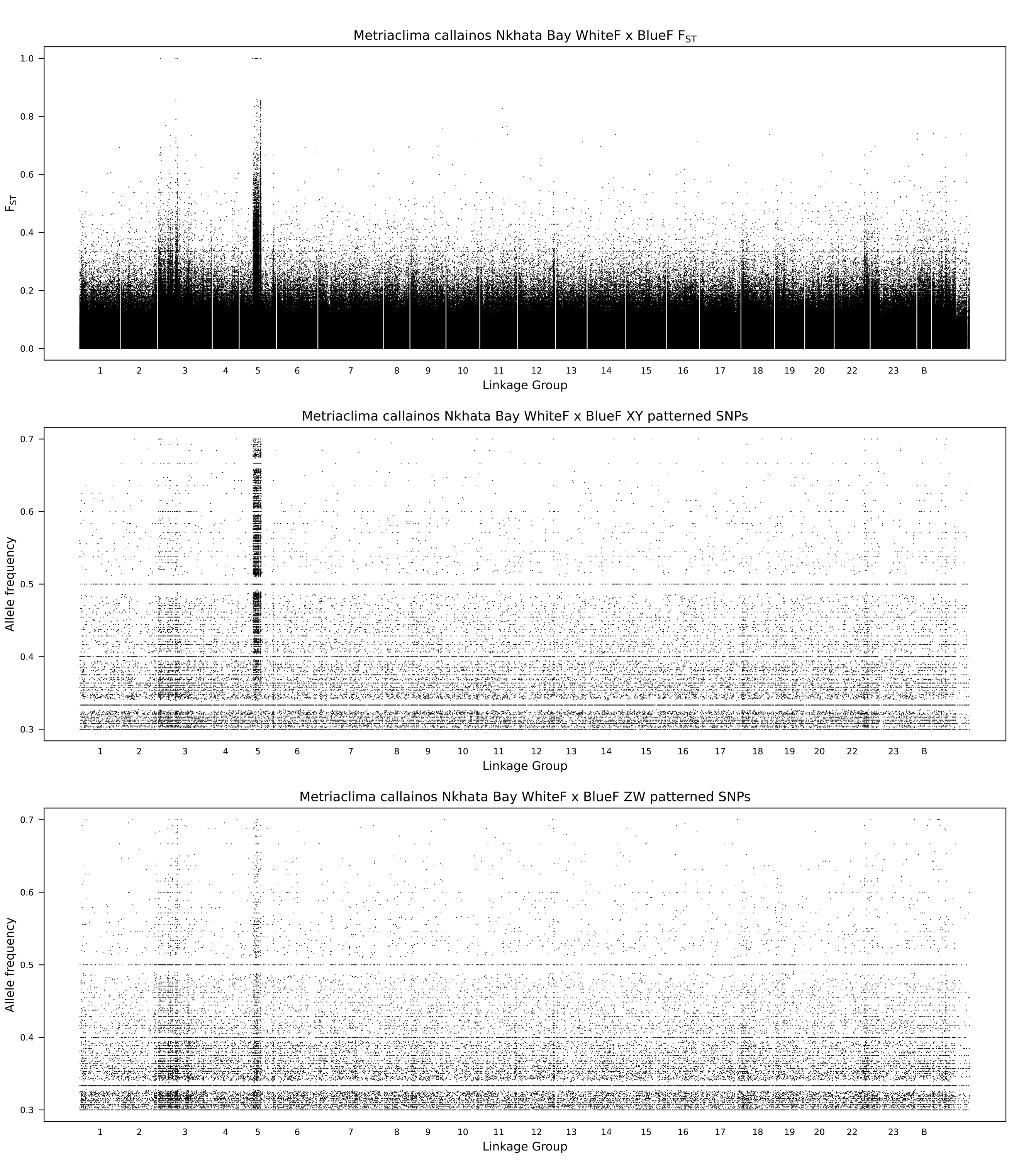


Page 12. *Metriaclima callainos*, Nkhata Bay, White females vs. Blue females for linkage group 5. A strong signal is observed in the Fst and XY-patterned SNPs because the White females are heterozygous for the inversion. (The XY-pattern is because of the order of comparison. The White females are heterozygous in this comparison).


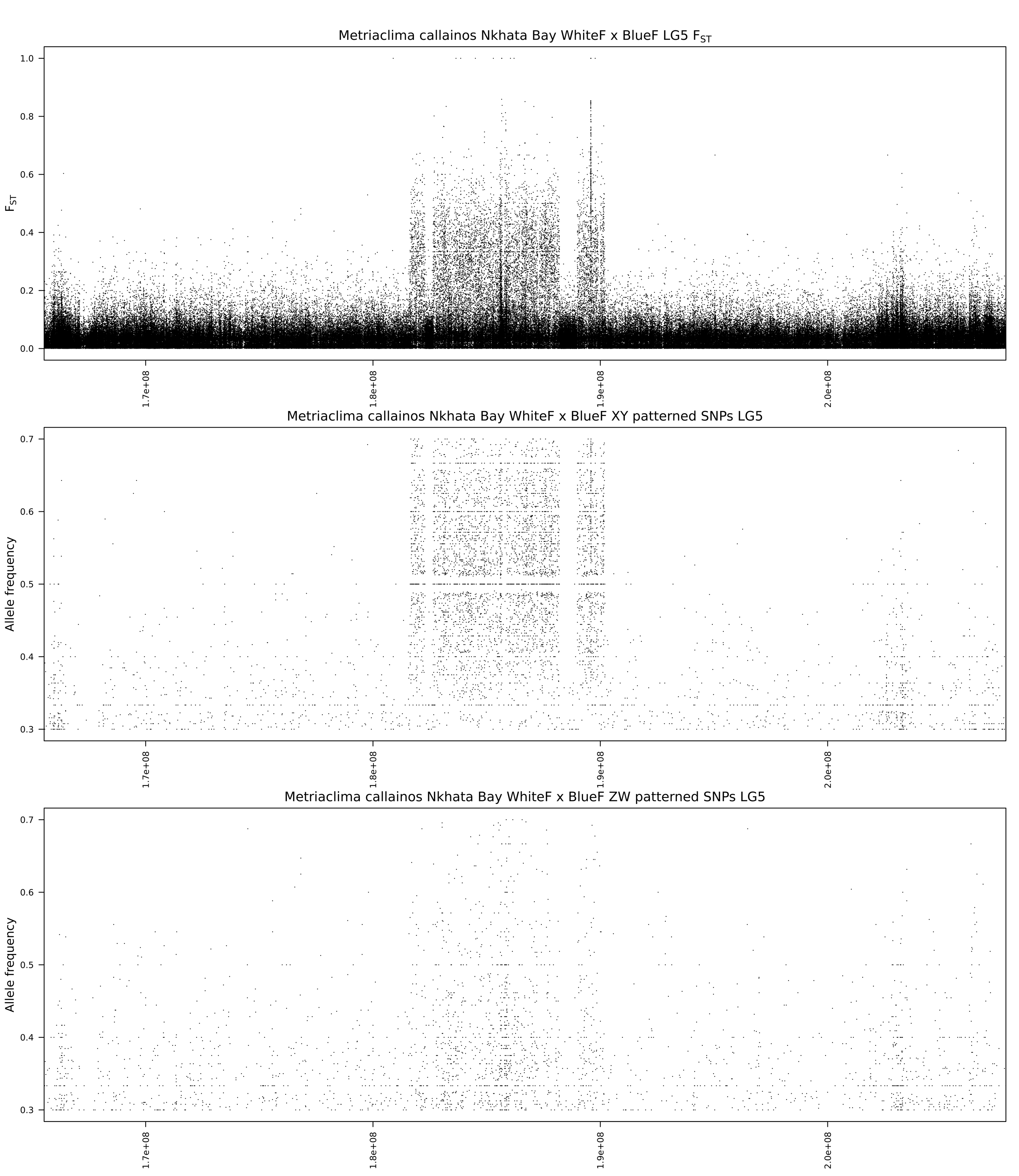


Page 13. *Metriaclima callainos*, Lupingu, White males vs. White females for whole genome. No signal is observed because the White males and White females carry the inversion at similar frequencies.


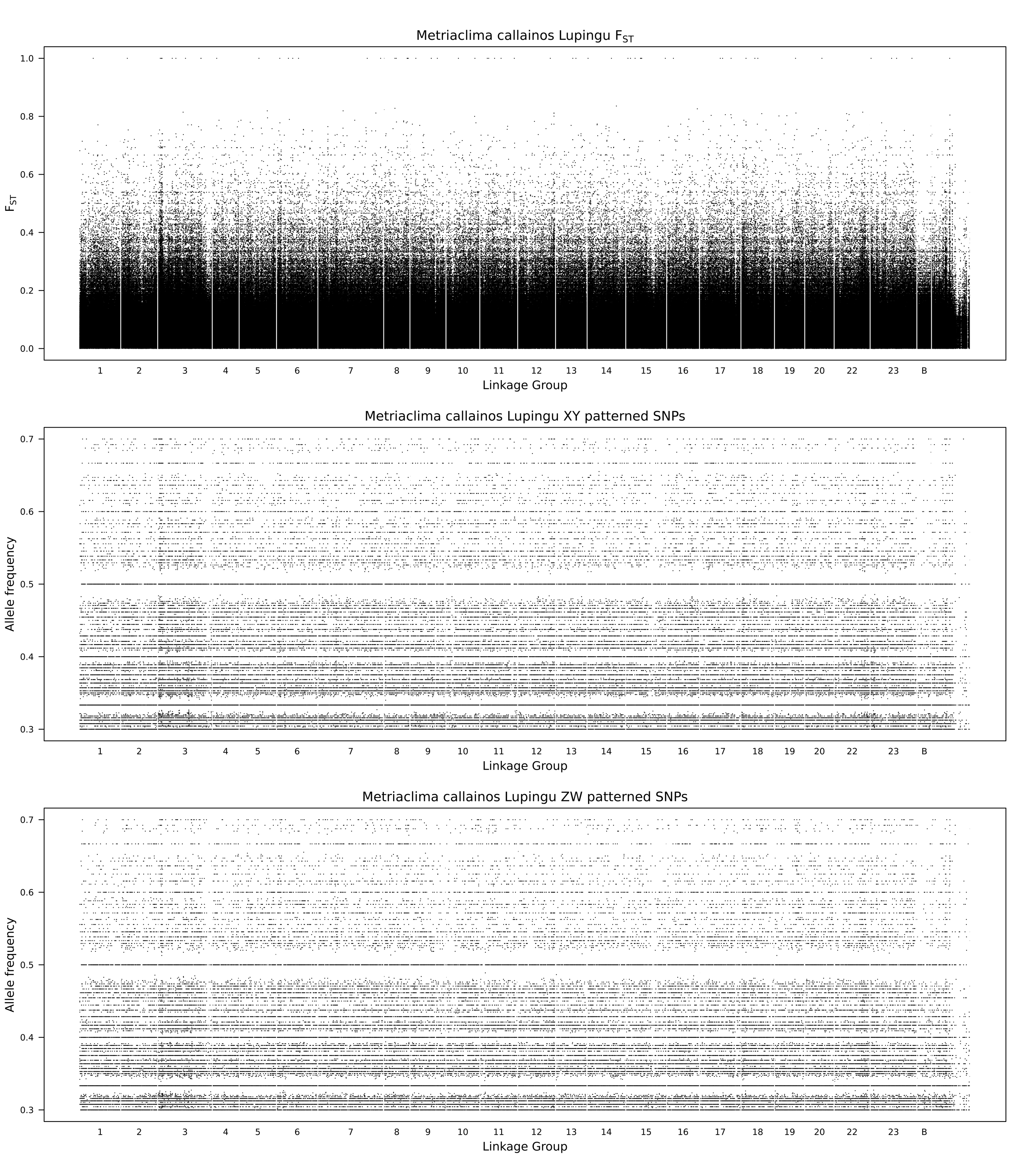


Page 14. *Metriaclima callainos*, Luwino, White males vs. White females for whole genome. No signal is observed because the White males and White females carry the inversion at similar frequencies.


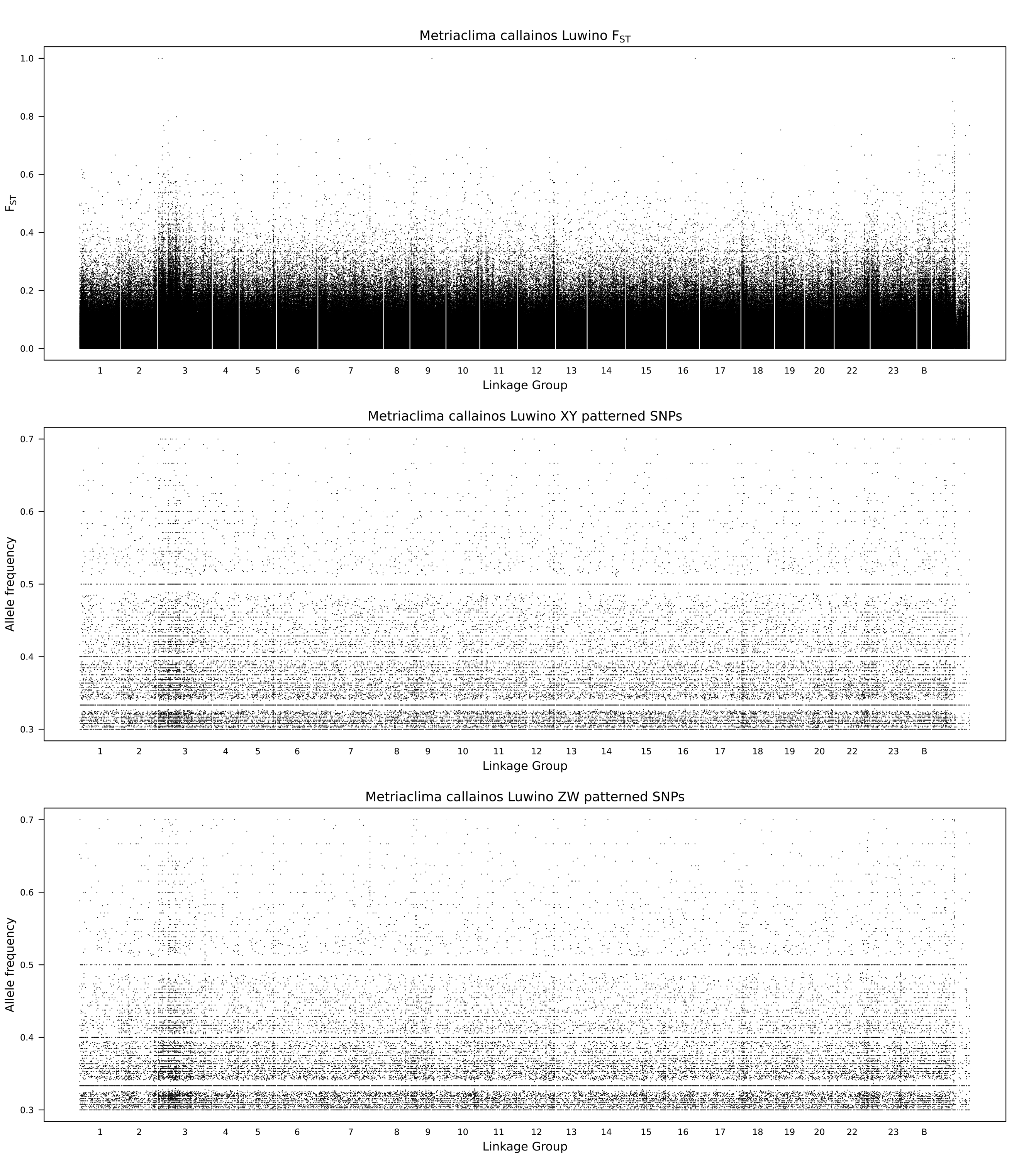


Page 15. *Metriaclima* 'zebra gold', Nkhata Bay, BB males vs. OB females for whole genome. A strong signal is observed in the Fst and ZW-patterned SNPs because the OB females are heterozygous for the inversion.


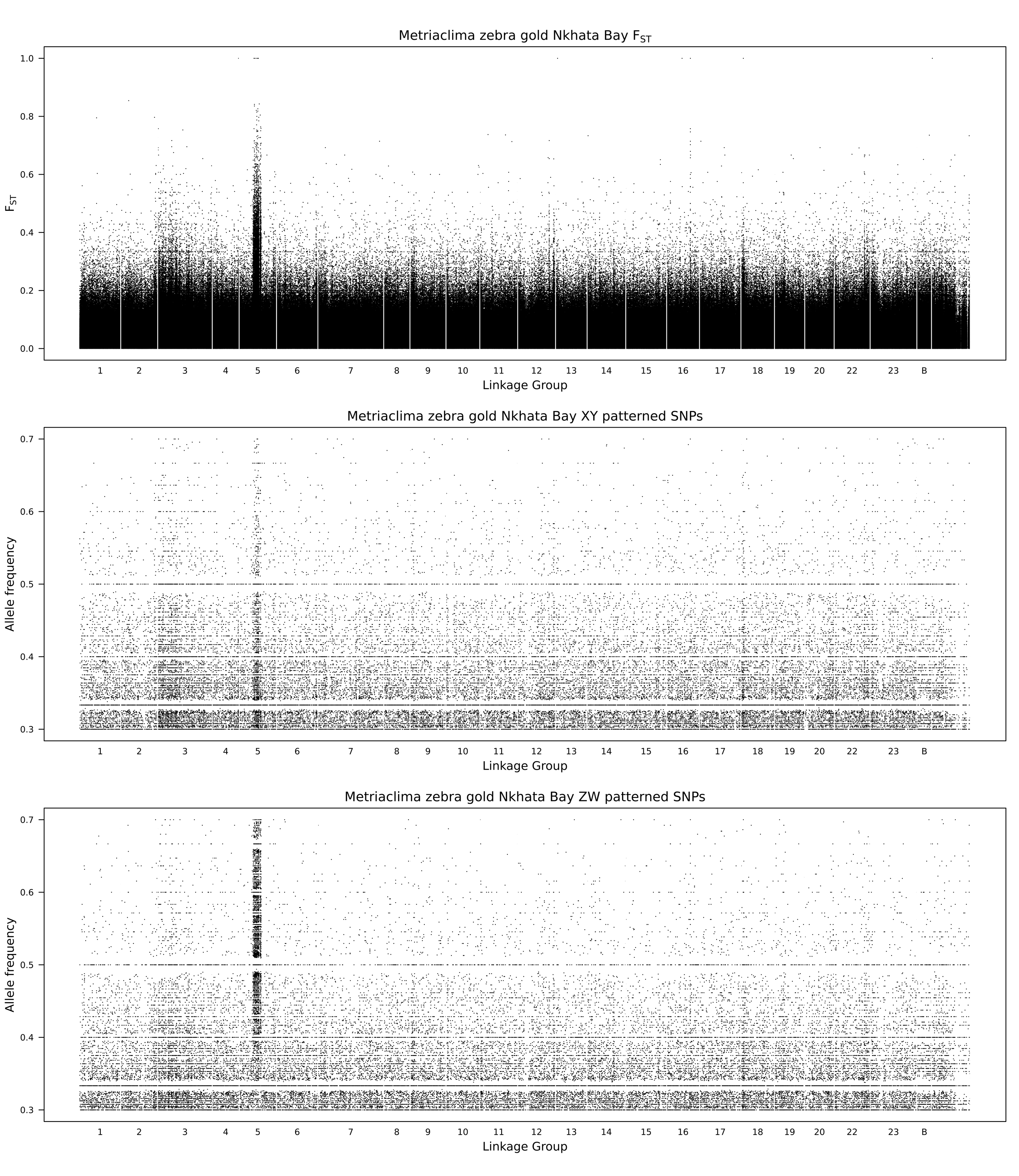


Page 16. *Metriaclima zebra*, Nkhata Bay, BB males x BB females for whole genome. No signal is observed because the inversion is not present in either sample.


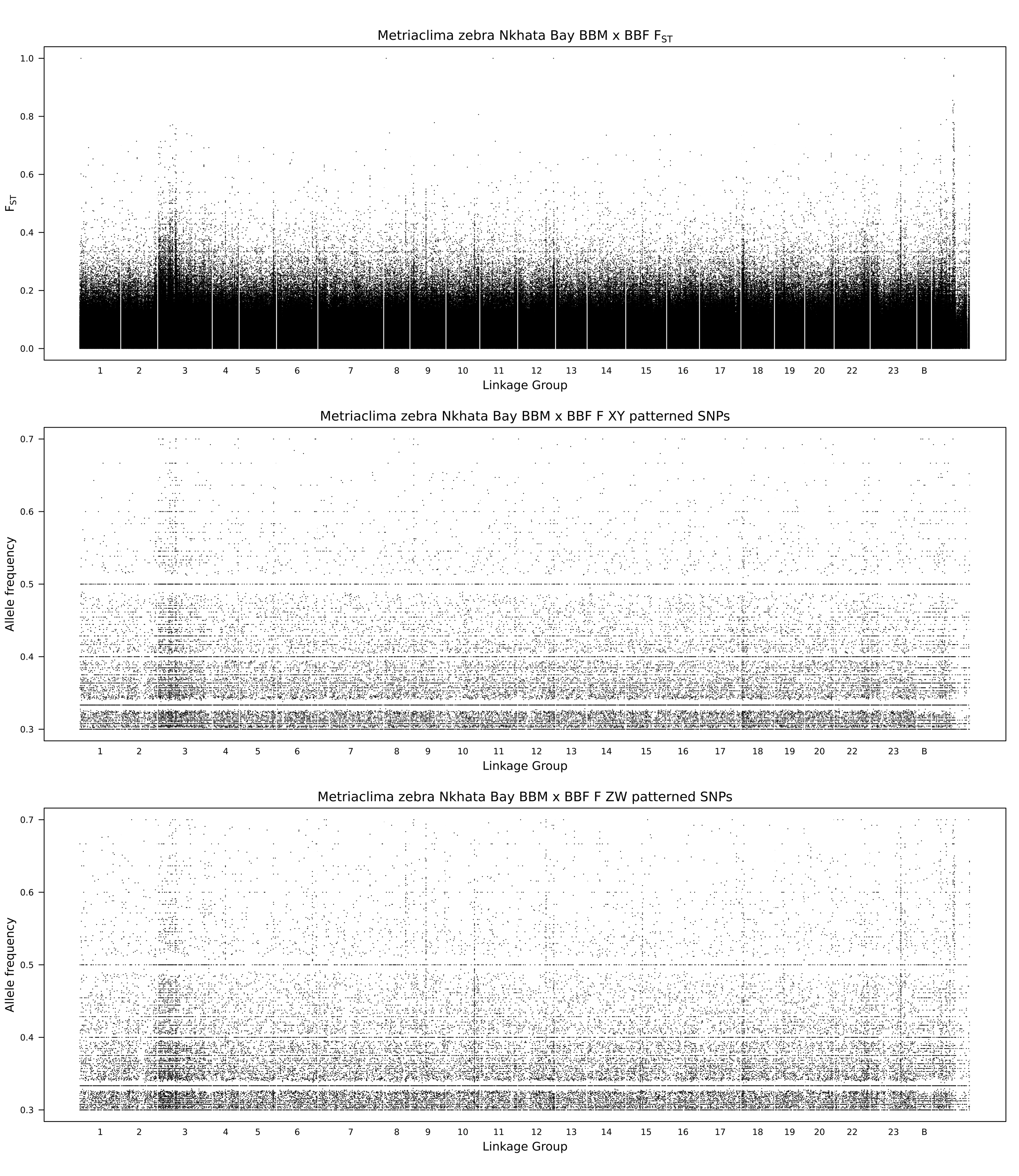


Page 17. *Metriaclima zebra*, Nkhata Bay, BB males x Orange females for whole genome. A strong signal is observed on linkage group 5 in the Fst and ZW-patterned SNPs because the Orange females are heterozygous for the inversion.


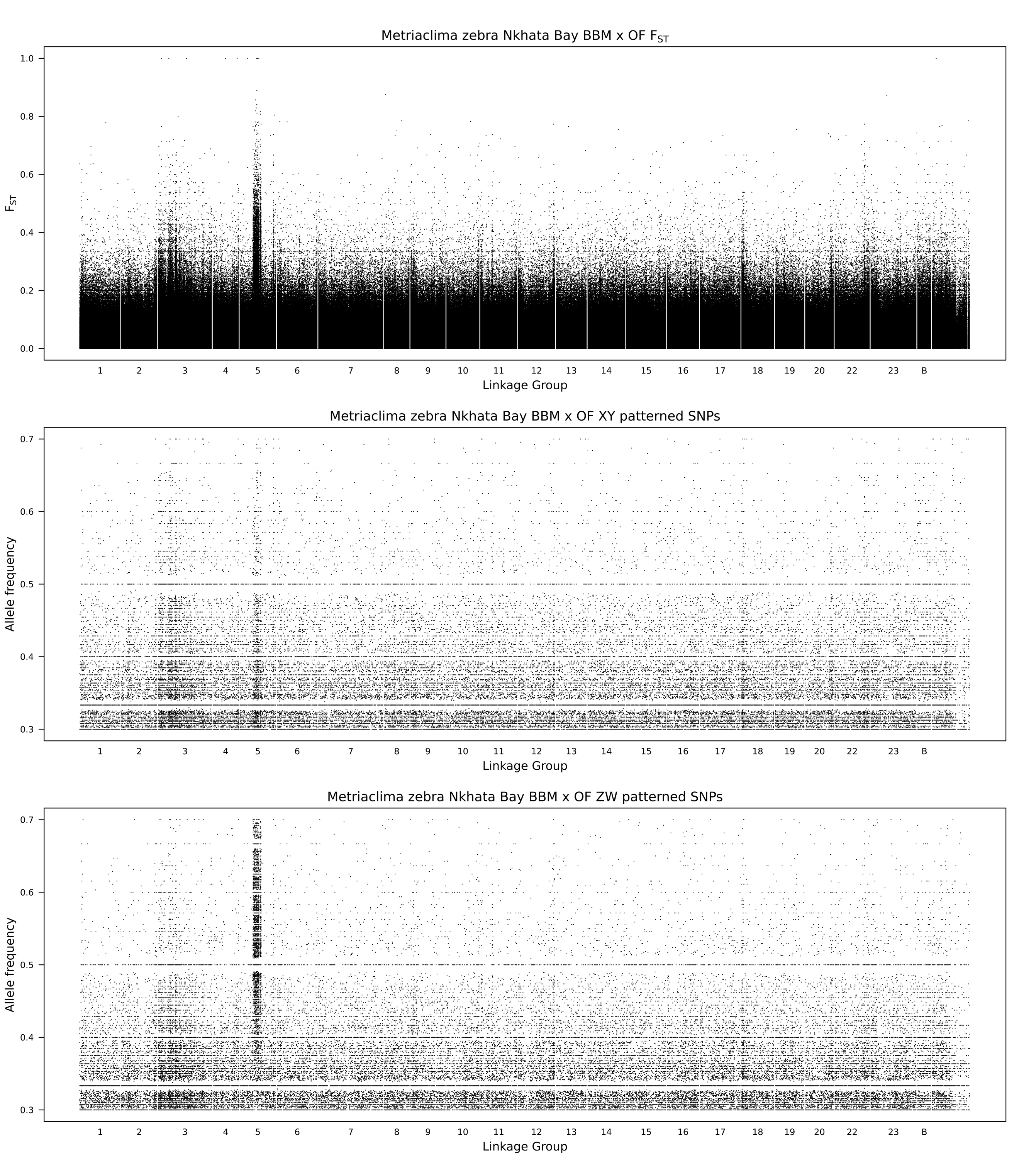


Page 18. *Metriaclima zebra*, Nkhata Bay, OB females x BB females for whole genome. A strong signal is observed on linkage group 5 in the Fst and ZW-patterned SNPs because the OB females are heterozygous for the inversion and the BB females are homozygous for the non-inverted haplotype.


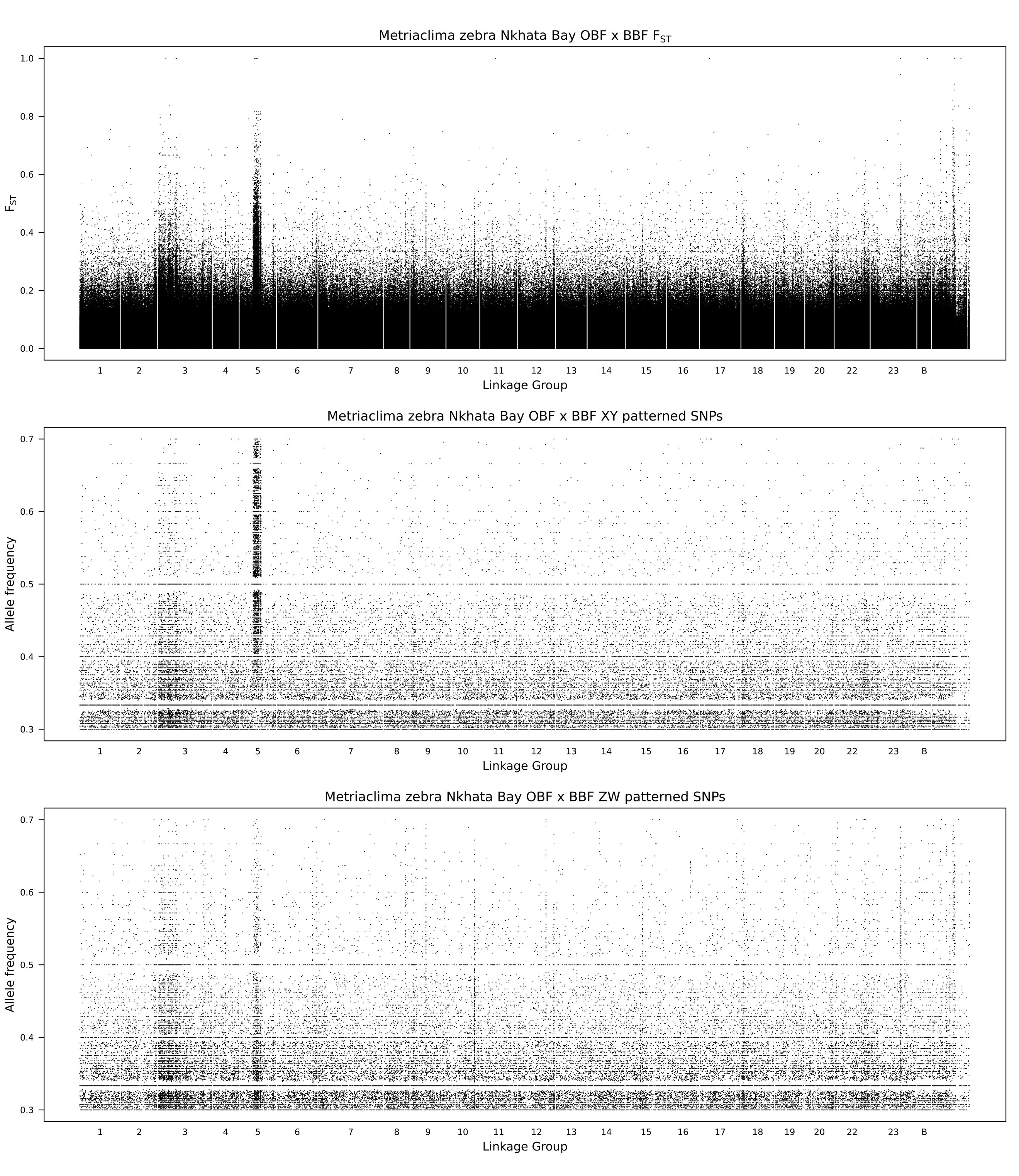


Page 19. *Metriaclima zebra*, Nkhata Bay, OB females x Orange females for whole genome. No signal is observed because OB females and Orange females are both heterozygous for the inversion.


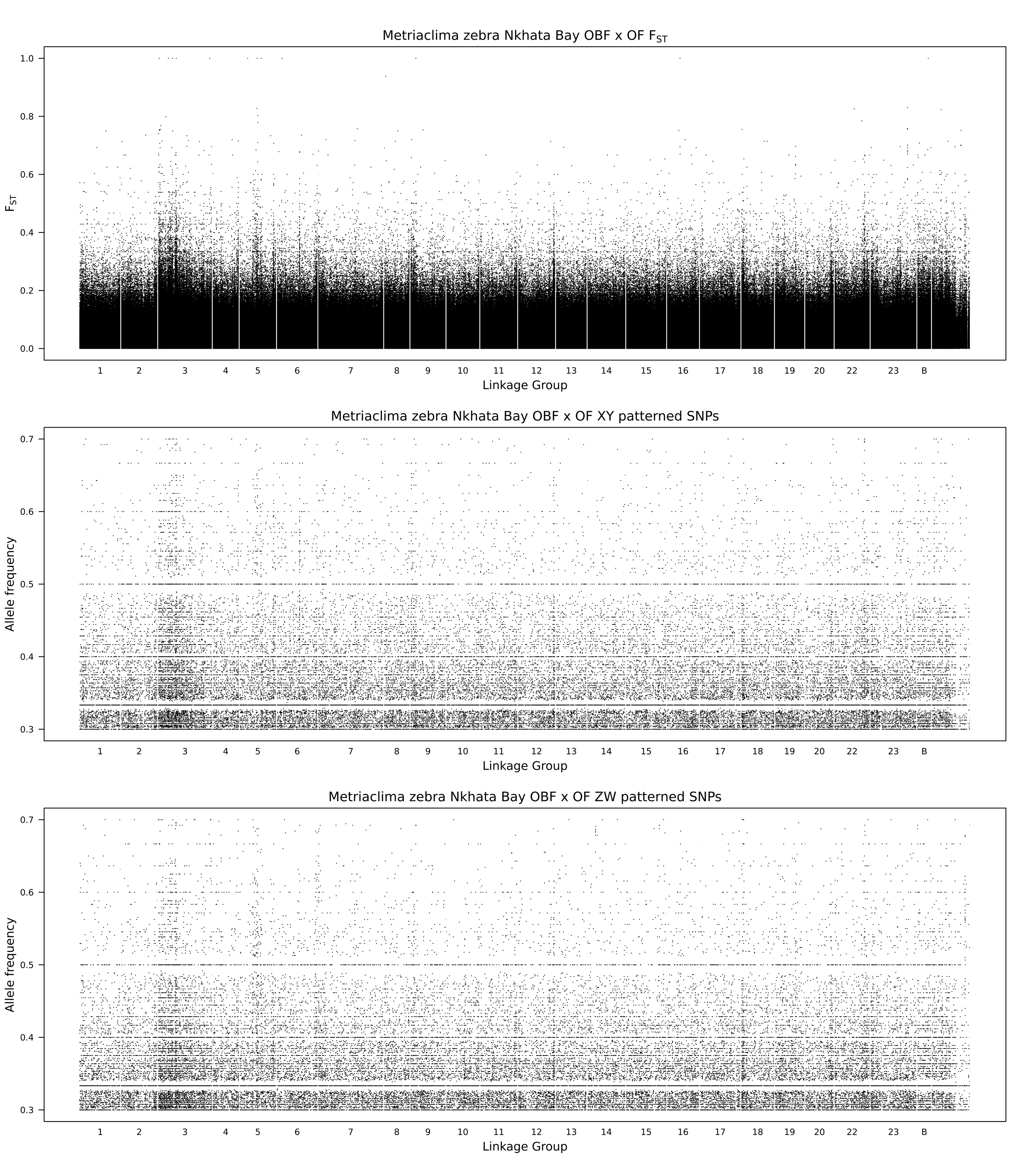


Page 20. *Metriaclima zebra*, Nkhata Bay, OB females x Orange females for linkage group 5. No signal is observed because OB females and Orange females are both heterozygous for the inversion.


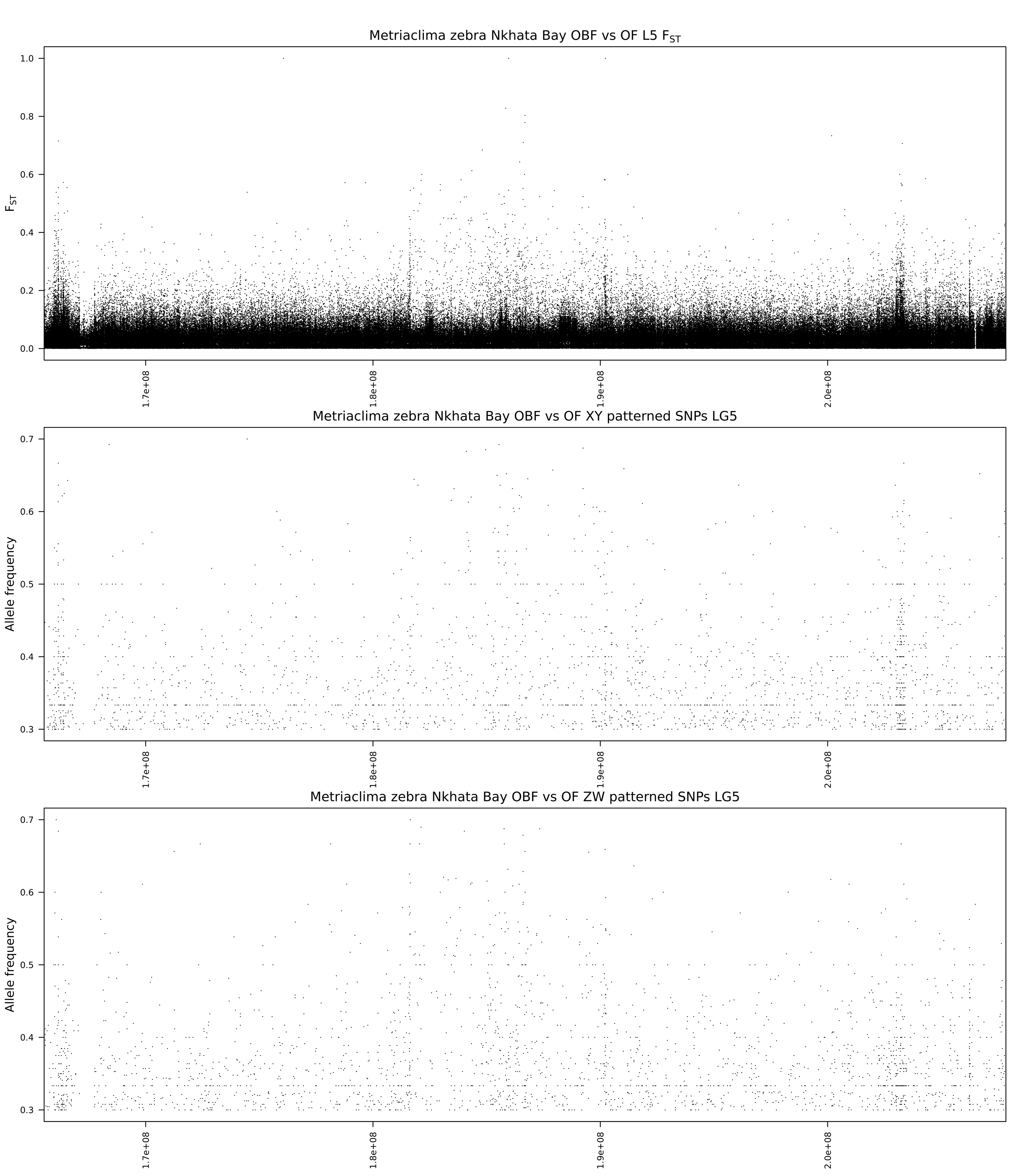


Page 21. *Labeotropheus fuelleborni*, Thumbi West, BB males x BB females for whole genome. No signal is observed because neither BB males or BB females carry the inversion.


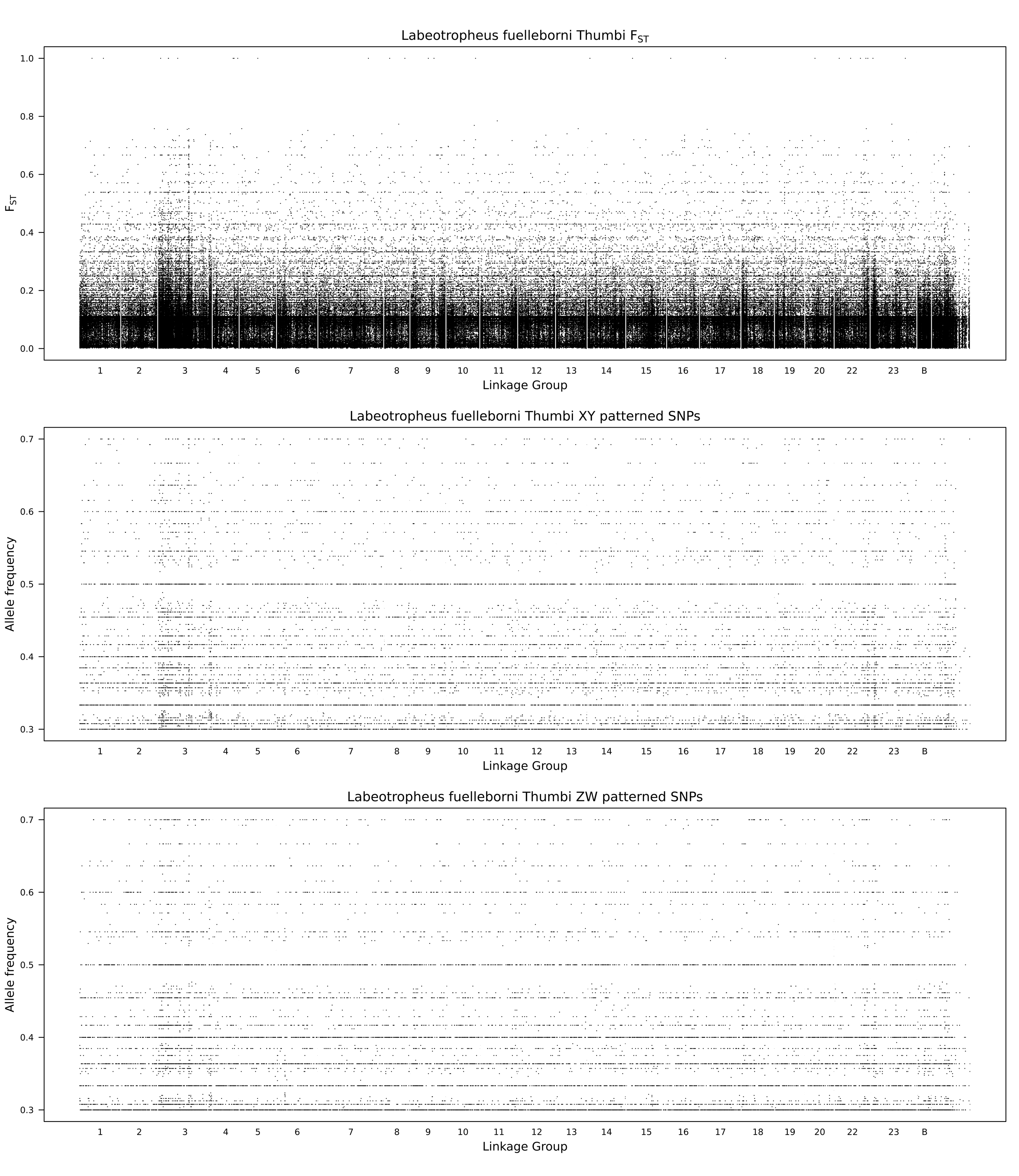


Page 22. *Labeotropheus trewavasae*, Thumbi West, BB males x BB females for whole genome. No signal is observed because neither BB males or BB females carry the inversion.


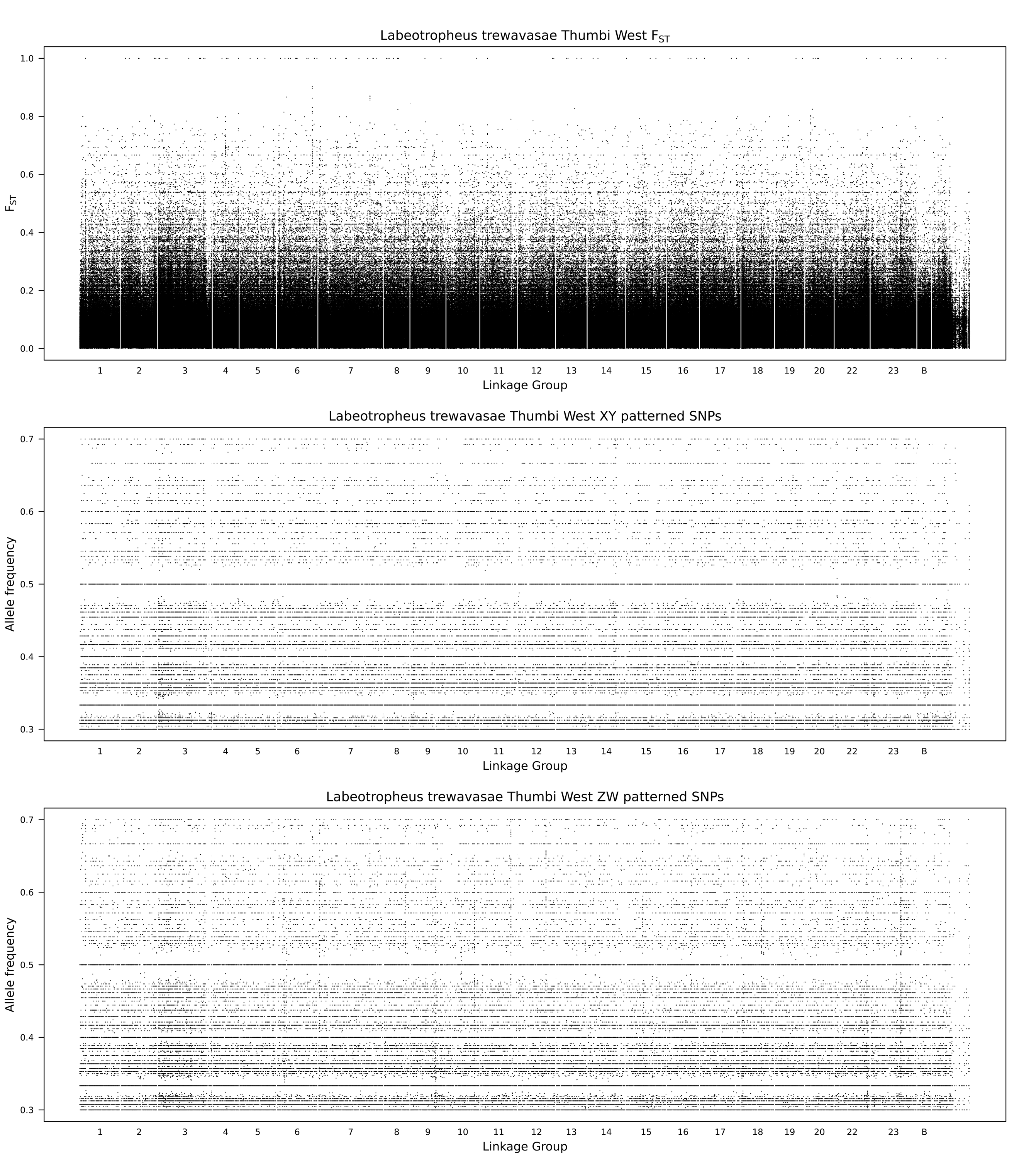


Page 23. *Metriaclima callainos*, Luwino White males vs. Nkhata Bay Blue females for whole genome. There is no evidence (XY signal due to the order of comparison) for an inversion on LG5.


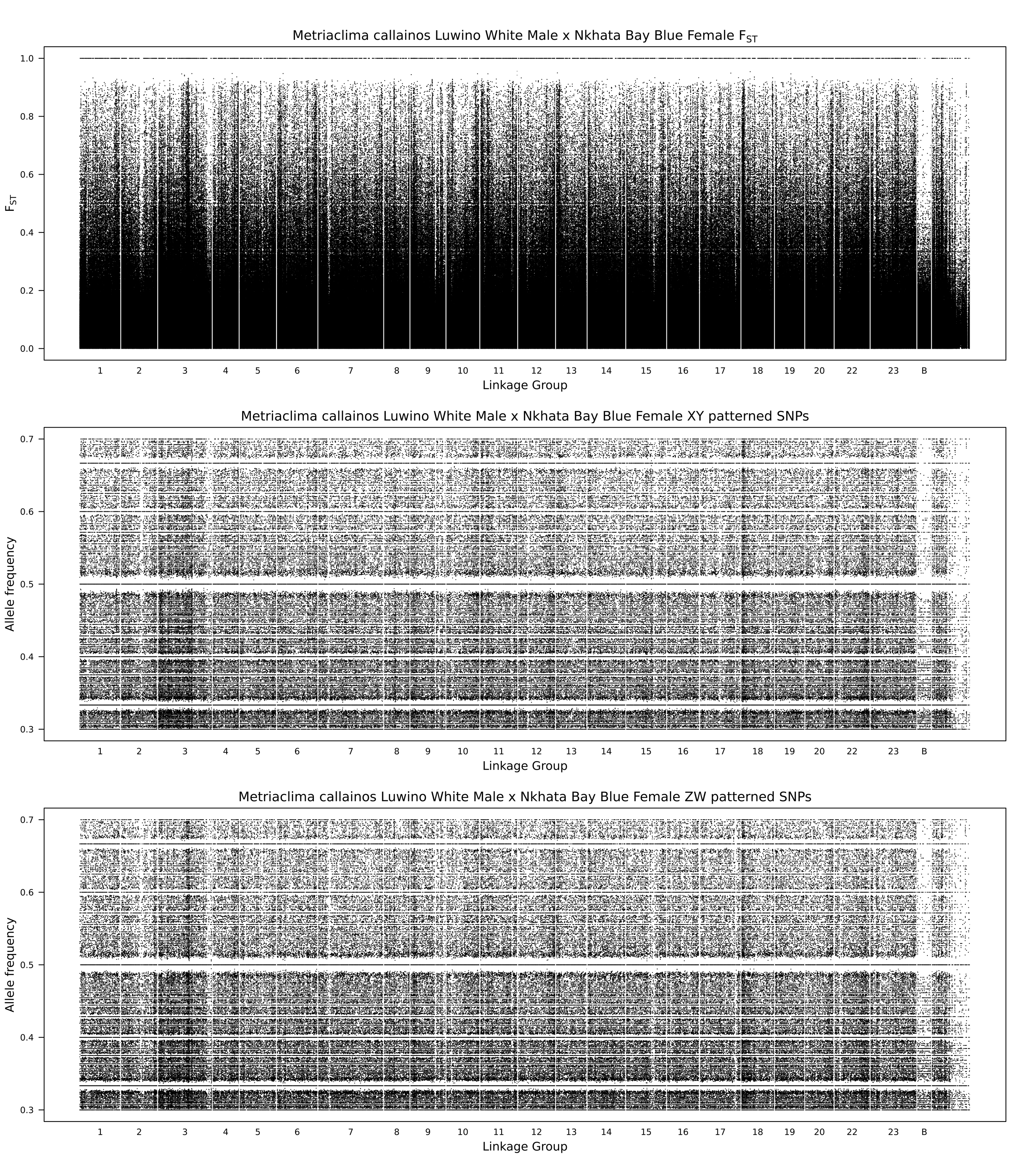


Page 24. *Metriaclima callainos*, Nkhata Bay Blue males vs. Luwino White females for whole genome. There is no evidence for a ZW signal expected if there was a sex-linked inversion on LG5.


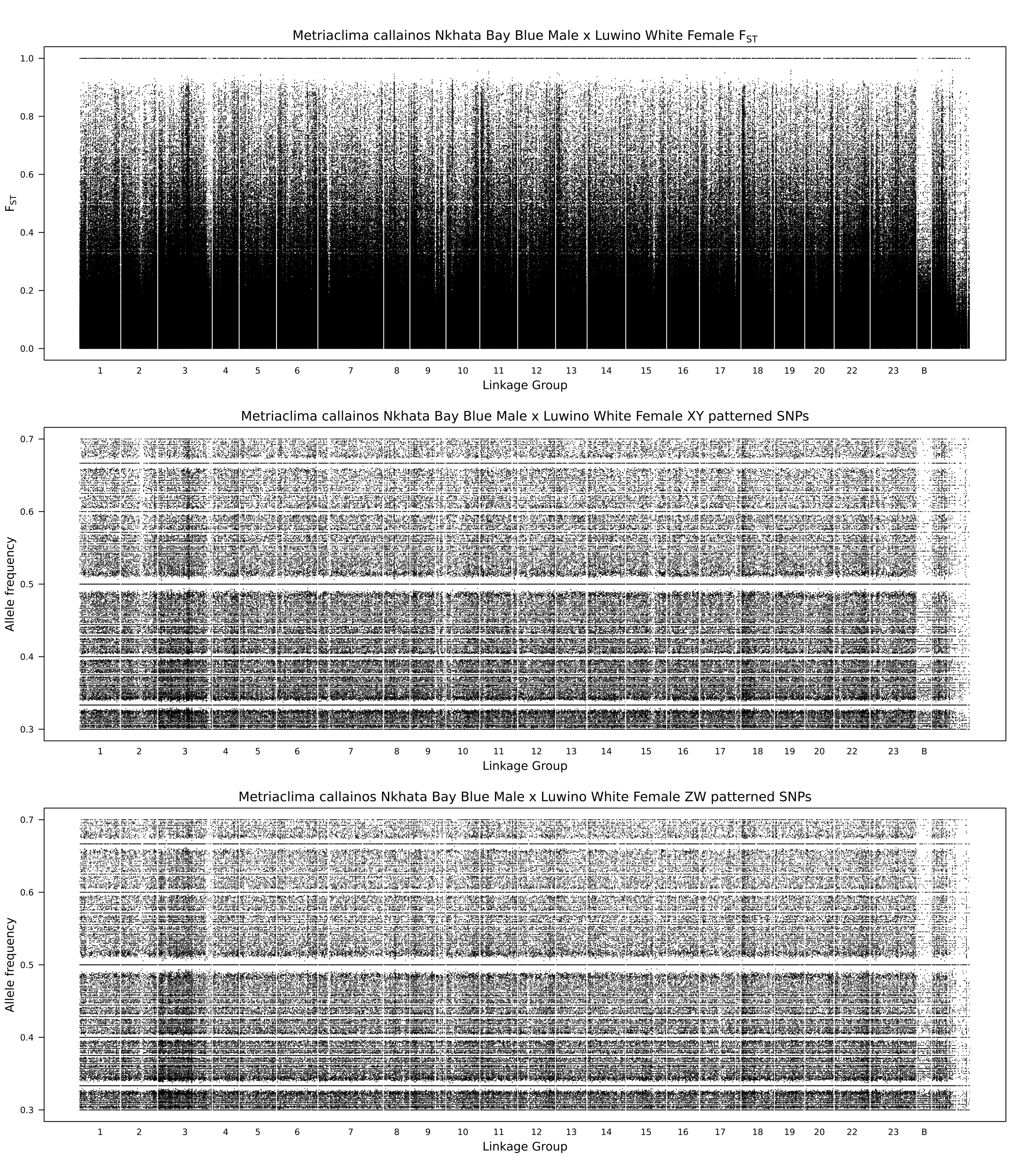


Page 25. *Metriaclima callainos*, Lupingu White males vs. Nkhata Bay Blue females for whole genome. There is no evidence (XY signal due to the order of comparison) for an inversion on LG5.


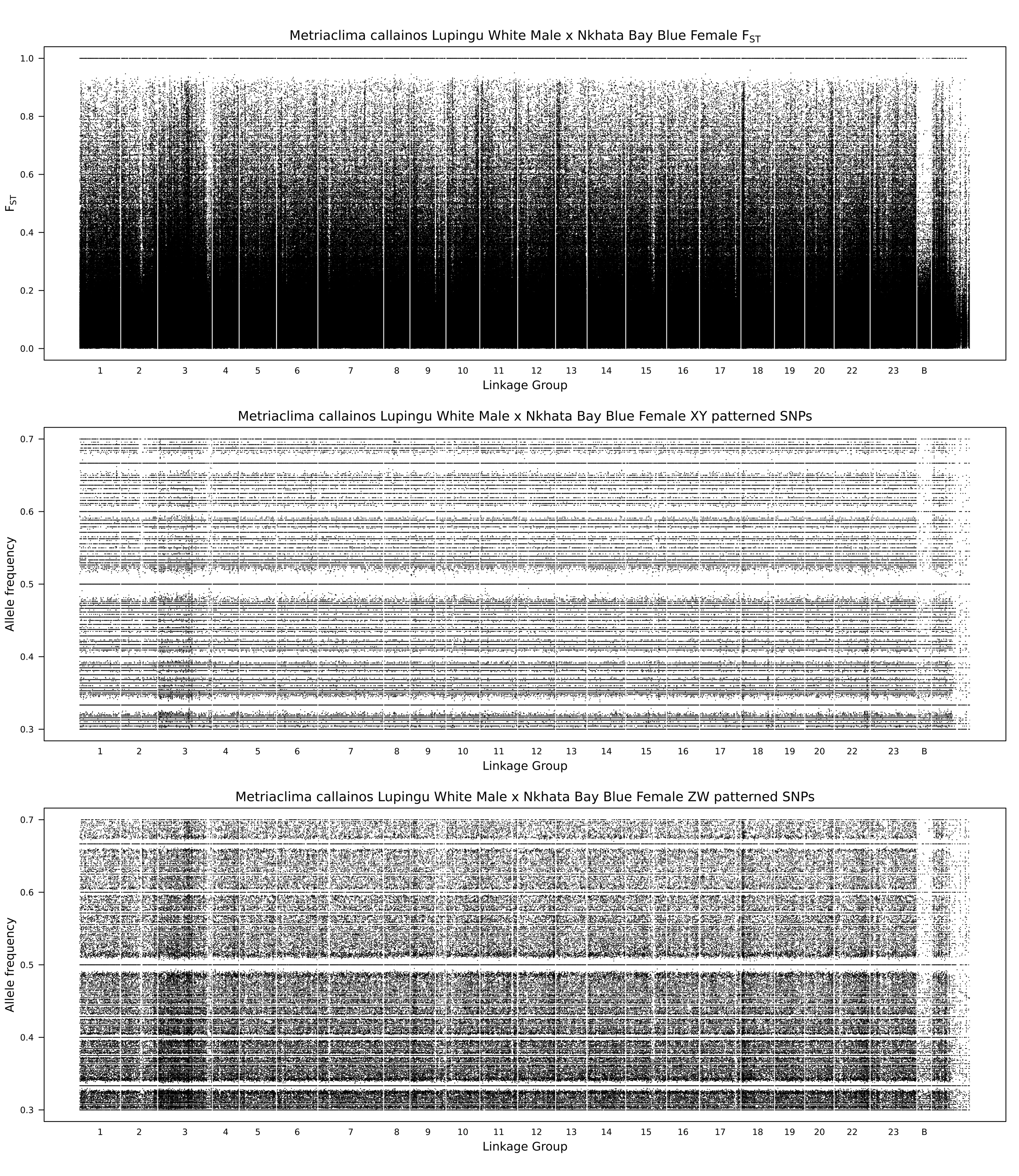


Page 26. *Metriaclima callainos*, Nkhata Bay Blue males vs. Lupingu White females for whole genome. There is no evidence for a ZW signal expected if there was a sex-linked inversion on LG5.


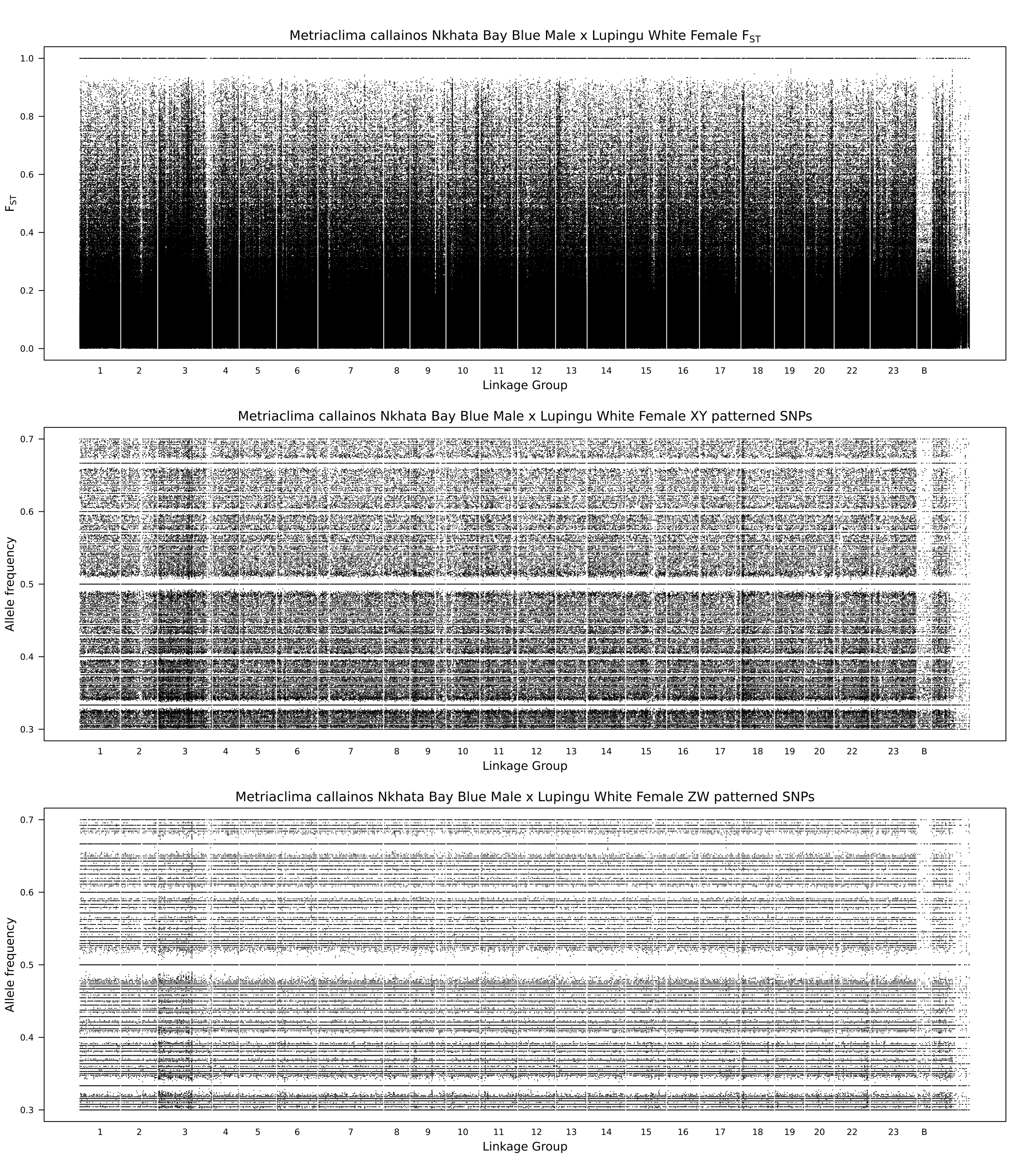

Supplement: Supplementary file 5 — Figure S5. [file MEC-34-e17821-s015.docx]
