# Supplementary material for: A Chromosome Inversion Creates a Supergene for Sex and Colour in Lake Malawi Cichlids
Source: Mol Ecol. 2025 Jun 10;34(20):e17821. doi: 10.1111/mec.17821 (PMC12530302; doi:10.1111/mec.17821)
Supplement: Supplementary file 7 — Figure S7. [file MEC-34-e17821-s009.docx]

**Supplemental Figure 7.** Frequencies of alleles at the Gata2 microsatellite in five populations segregating OB, O or W morphs. The columns represent populations of *Metriaclima zebra* (Nkhata Bay), *M.* 'zebra gold' (Nkhata Bay), *M. callainos* (Nkhata Bay), *M. callainos* (Luwino Reef) and *Labeotropheus trewavasae* (Maison Reef). The rows represent cognate morphs: Row 1 BB or B males; Row 2 BB or B females; Row 3 OB females or W males; Row 4 O or W females. Nef is the effective number of alleles (1/1-H). The most common allele in the OB morphs of *L. trewavasae* (Maison Reef) is different from that in the BB males and females.
